# Nanobio Interface Between Proteins and 2D Nanomaterials

**DOI:** 10.1021/acsami.3c04582

**Published:** 2023-07-24

**Authors:** Shounak Roy, Aastha, Kaivalya A. Deo, Kashmira Dey, Akhilesh K. Gaharwar, Amit Jaiswal

**Affiliations:** † School of Biosciences and Bioengineering, Indian Institute of Technology, Mandi, Kamand, Mandi, Himachal Pradesh 175075, India; ‡ Department of Biomedical Engineering, College of Engineering, Texas A&M University, College Station, Texas 77843, United States; § Interdisciplinary Graduate Program in Genetics and Genomics, Texas A&M University, College Station, Texas 77843, United States

**Keywords:** 2D nanomaterial, protein corona, surface energy, interaction forces, analytical tools

## Abstract

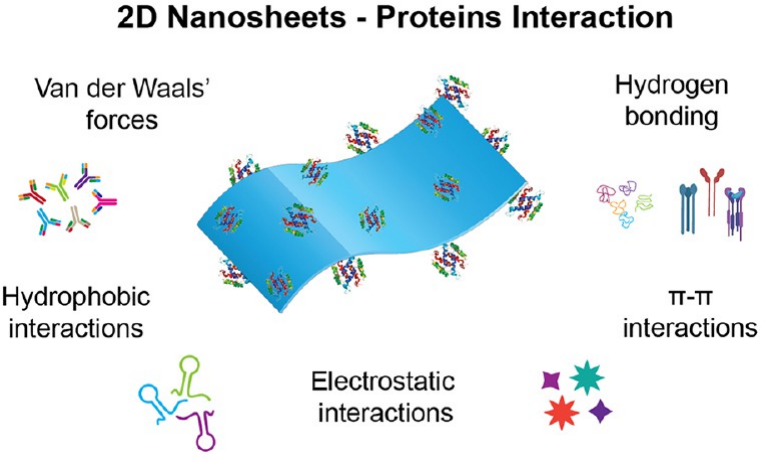

Two-dimensional (2D) nanomaterials have significantly contributed to recent advances in material sciences and nanotechnology, owing to their layered structure. Despite their potential as multifunctional theranostic agents, the biomedical translation of these materials is limited due to a lack of knowledge and control over their interaction with complex biological systems. In a biological microenvironment, the high surface energy of nanomaterials leads to diverse interactions with biological moieties such as proteins, which play a crucial role in unique physiological processes. These interactions can alter the size, surface charge, shape, and interfacial composition of the nanomaterial, ultimately affecting its biological activity and identity. This review critically discusses the possible interactions between proteins and 2D nanomaterials, along with a wide spectrum of analytical techniques that can be used to study and characterize such interplay. A better understanding of these interactions would help circumvent potential risks and provide guidance toward the safer design of 2D nanomaterials as a platform technology for various biomedical applications.

## 1 Introduction

**Figure 1:**
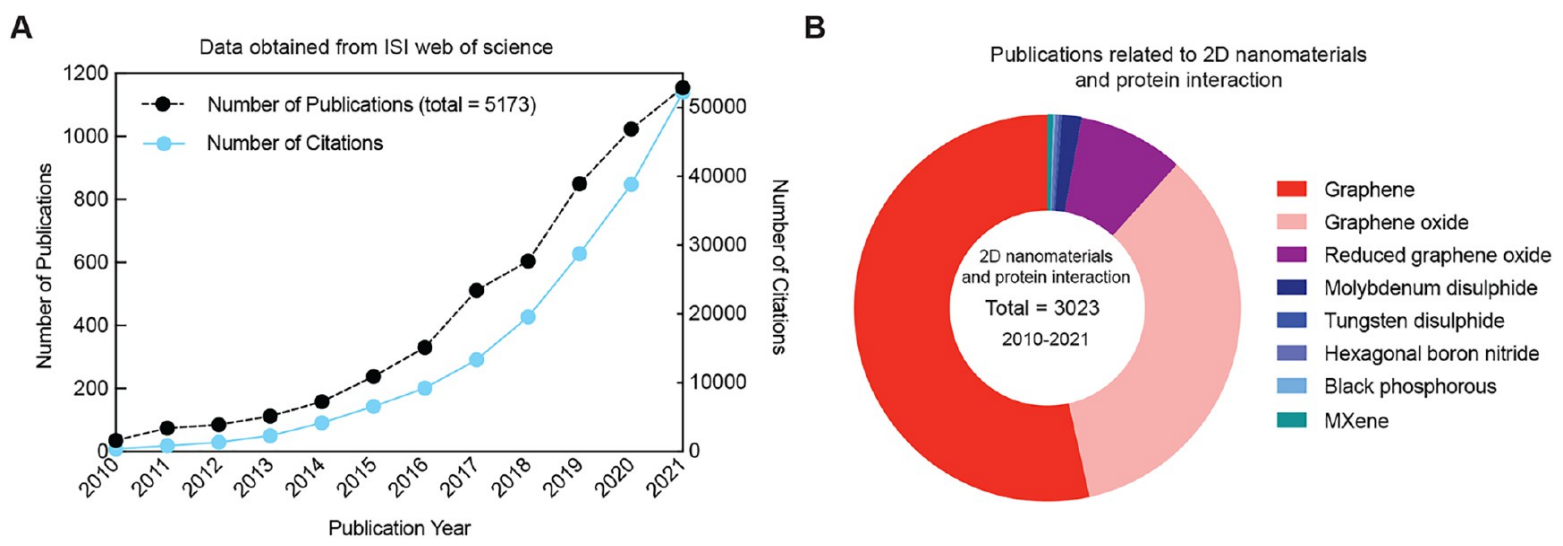
(A) ISI Web of Science survey for number of publication and number of citations using ‘*2D Nanomaterial*’ as keyword (obtained until December 2021). Exponential increase in the field of 2D Nanomaterials as evident from number of publications in past decade (2010–2021). (B) Publications related to 2D nanomaterials and protein interactions in the past decade (2010–2021). Data obtained from ISI Web of Science using “*2D Nanomaterial”* AND “*protein*” OR “*Graphene*”, “*Graphene oxide*”, “*Reduced graphene oxide*”, “*Molybdenum disulfide*”, “*Tungsten disulfide*”, “*Hexagonal boron nitride*”, “*Black phosphorous*”, “*MXene*” (obtained December 2021).

Two-dimensional (2D) nanomaterials have significantly contributed to recent advances in material sciences and nanotechnology, owing to their layered structure. The introduction of the facile graphene synthesis method by Novoselov and Geim using the scotch tape technique provided a significant boost in the understanding and utilization of 2D nanomaterials for a wide range of applications.^1^ This led to the discovery of other 2D materials such as transition metal dichalcogenides, hexagonal boron nitride, and black phosphorus. Over the past decade, the field of 2D nanomaterials has grown rapidly and exponentially with the discovery of new materials, associated properties, and a wide range of applications (Figure 1). 2D nanomaterials are essentially sheet-like, flat structures where the lateral size is usually larger than 100 nm and up to a few micrometers, but a thickness of only of a few atomic layers. These nanomaterials not only are diverse in their mechanical, optical, and chemical properties but also exhibit uniqueness in their size, shape, biocompatibility, and biodegradability. These properties make them excellent candidates for different biological applications such as drug delivery, tissue engineering, imaging, and biosensing.^2−6^ 2D nanomaterials are a few atoms thick which implies that they have a huge surface area. This attribute makes them indispensable for applications requiring the highest level of surface interactions on a small scale. They have high capacity to adsorb molecules and can even govern or enable triggered release which has led to their applications in drug delivery.^7,8^ The exceptionally thin structure of 2D nanomaterials allows them to respond to external stimulus such as light, which is being utilized in designing stimuli responsive therapies such as photothermal therapy, photodynamic therapy, etc.^9,10^

In order to harness the potential of 2D nanomaterials for biomedical applications, it is essential to gain a thorough understanding of their interactions with biomolecules found in physiological environments.^11^ Predominantly composed of various proteins, these environments are the arenas where the critical processes of life unfold. Proteins are essentially the workhorses of living systems, crucial for maintaining cellular structure, function, and regulation. When introduced into body fluids such as blood, a physiological medium, these nanomaterials first come into contact with a diverse array of proteins. Quickly, these proteins bind to the surface of the nanosheets, forming a coating around them. This phenomenon is known as “protein corona” formation. The type of protein corona formed on 2D nanomaterials are dependent on the surface chemistry, surface charge and hydrophobicity of both nanomaterials and protein. These interactions eventually dictate various biological outcome such as cellular internalization, circulation time, immune response and clearance. For example, when a 2D nanomaterials is covered by surface bound proteins, it enhances the chances of cellular uptake of the nanosheets and even activates intracellular signaling which aids in the energy-dependent cellular uptake processes.^12−15^ On the other hand, there can also be a possibility of structural changes being induced in the proteins upon interaction with 2D nanomaterials, which ultimately results in an altered regulation of the biological function of the protein. Thus, a thorough understanding of the effect of the physiological environment on the 2D nanomaterials and vice versa, as well as a deeper knowledge of the possible interactions with a nanomaterial which a protein might face, would add a much-needed boost to the effective development of 2D nanomaterials for biomedical applications.

The protein-coated 2D nanomaterials may attach to the external surface of a cell, causing localized perturbations in the cell membrane. Additionally, they can integrate themselves into the lipid bilayer of the plasma membrane or translocate across membranes to access cytoplasmic or endolysosomal compartments.^16,17^ The cellular uptake of nanosheets is size-dependent. Large nanosheets generally undergo phagocytosis or macropinocytosis, while smaller ones are mainly internalized via endocytosis, either caveolar or clathrin-mediated.^18^ These interactions can influence the cell system in varied ways, causing either beneficial or detrimental effects. Simulation studies on graphene and its derivatives have shown direct interactions with cell membranes, which can lead to cell damage and subsequent cell death.^19^ However, other studies have reported mammalian cell growth following interactions with graphene.^20,21^

Similarly, MoS_2_ nanosheets demonstrated cytotoxicity for cancer cells with minimal impact on normal cell lines, making them a potential candidate for anticancer systems.^18^ MXenes, unless coated with biomolecules like PEG, chitosan, PLGA, or collagen was found to have cytotoxic effect.^22^ It was also reported to be excreted from mice via urine and feces. However, the elucidation of MXenes interaction with cells is yet to be explored. hBN and Xenes nanosheets, like other nanosheets have promising effect in bone scaffolding and wound healing, although the exact mechanism for the same still needs to be studied. In general, all 2D nanomaterials interact with different cells and tissues in a morphology-, size-, and concentration-dependent manner. For a detailed understanding of interactions of graphene,^23^ hBN,^23^ MoS_2_,^24^ MXenes,^22^ and Xenes^25^ with biological moieties, we encourage the readers to refer to some excellent reviews available on this topic.

To evaluate the influence of 2D nanomaterials on the structure and function of proteins, a comprehensive study and discussion on such interactions is necessary. Thus, in this review we concentrate on the families of 2D nanomaterials that have considerably contributed toward biomedical research in the past decade. We will discuss about the different interactive forces responsible for protein-2D nanomaterial interactions along with the analytical tools that can be used to study such interactions. A brief overview about the different types of 2D nanomaterials, e.g., graphene, transition metal dichalcogenides (TMDCs), 2D monoelemental materials (Xenes), borophene, phosphorene, etc.,is presented. We will discuss about their structure, surface characteristics, state of hybridization, surface charges (if present) and functional groups available.^26−29^ The interactive forces like hydrophobic, electrostatic, vdW forces, etc., that may exist between proteins and 2D nanomaterials are elaborated using examples of various proteins like albumin, chymotrypsin, HRP, etc., and different 2D nanomaterials. The review also provides a detailed insight on how to study and characterize these protein-2D nanomaterials complexes and their interactions using different analytical tools and techniques like microscopy, spectroscopy, molecular dynamic simulations, etc. The interaction between 2D nanomaterials and proteins can be employed for multiple applications including biomedical applications such as for drug delivery, bioimaging, diagnostics, antibacterial, tissue engineering, etc.^30−34^ Overall, a comprehensive discussion on the different aspects of interaction between proteins and 2D nanomaterials and its surrounding concepts are elucidated in this review.

## 2 Types of 2D Nanomaterials

**Figure 2:**
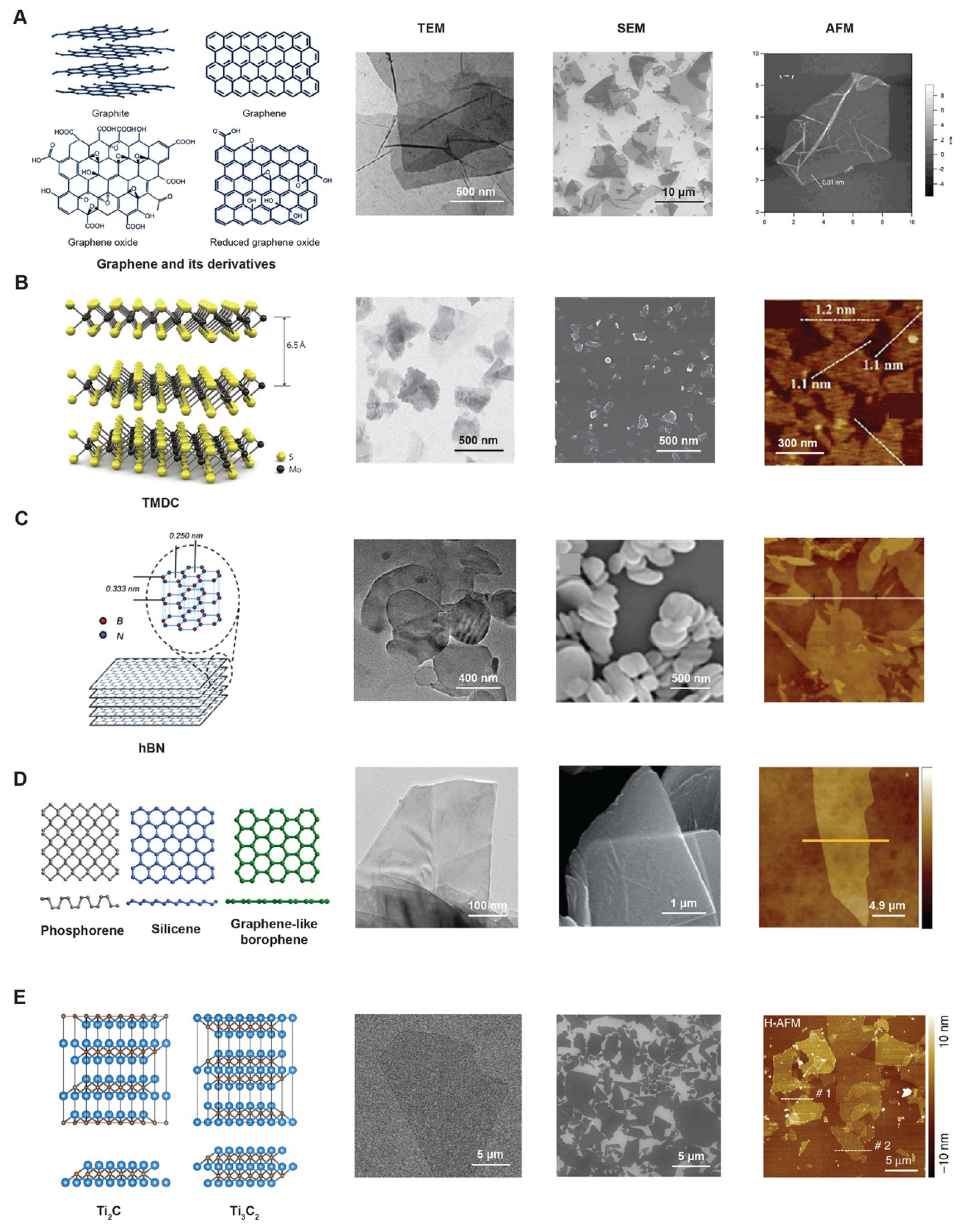
Types of 2D nanomaterials. (A) Schematic representation of the atomic framework of graphite, graphene, graphene oxide (GO), and reduced graphene oxide (RGO). Reproduced with permission from ref (51). Originally published by Dove Medical Press Ltd.^51^ Electron micrographs showing the morphology of GO nanosheets. Reproduced with permission from refs (52) and (53). Copyright 2011 Springer Nature and Copyright 2016 National Academy of Sciences. (B) Schematic representation of the atomic framework of TMDCs. Reproduced with permission from (54). Copyright 2011 Springer Nature. Electron micrographs showing the morphology of molybdenum disulfide nanosheets. Reproduced with permission from refs (31) and (55). Copyright 2019 American Chemical Society and Copyright 2019 Frontiers. (C) Schematic representation of the atomic framework of h-BN. Reproduced with permission from ref (56). Copyright 2012 Royal Society of Chemistry. Electron micrographs showing the morphology of hexagonal boron nitride nanosheets. Reproduced with permission from refs (57) and (58). Copyright 2017 Springer Nature and Copyright 2019 Elsevier. (D) Schematic representation of the atomic framework of monolayer Xenes. Reproduced with permission from ref (59). Copyright 2019 John Wiley and Sons. Electron micrographs showing the morphology of black phosphorus nanosheets. Reproduced with permission from refs (60) and (61). Copyright 2017 John Wiley and Sons and Copyright 2016 Royal Society of Chemistry. (E) Schematic representation of the atomic framework of MXenes. Reproduced with permission from ref (62). Copyright 2019 Elsevier. Electron micrographs showing the morphology of Ti_3_C_2_ nanosheets. Reproduced with permission from ref (63). Copyright 2020 Springer Nature.

Discovery of “Graphene - the first modern 2D material” in 2004, led to the era of 2D nanomaterial synthesis and application. Materials having one dimension of nanosize, resembling a large but thin sheet, are referred as 2D nanosheets/2D nanomaterials.^35^ 2D nanosized materials show novel physicochemical properties as compared to their bulk counterparts. Atomically thin crystalline 2D nanomaterials possess covalent bonding and vdW interactions as intralayer and interlayer bonding, respectively. The presence of limited atomic layers, large surface area, a surface state free nature, dangling bond-free surface and high mobility of charge carriers, are responsible for exceptional properties of 2D nanomaterials such as high thermal and electrical conductivity, higher optical resolution, improved chemical interactions and higher mechanical strength.^36−42^ These properties could be deployed for many purposes, such as in chemical/biosensors, electronic and optoelectronic devices, drug delivery systems, bioimaging, tissue engineering, photothermal catalysis, and energy storage and conversion.^30,37,40,41,43,44^ The following section provides a brief overview on the different types of 2D nanomaterials that are extensively explored in the field of biomedical research (Figure 2).

### 2.1 Graphene and Its Derivatives Like GO (GO)/Reduced GO (RGO)

Graphene is a carbon based single layered structure, packed in hexagonal (honeycomb) lattice, having a bond length of 0.142 nm between two carbon atoms (Figure 2A). Graphite was exfoliated to single-atom-layer carbon to form graphene.^1^ Since then, this great discovery has led to the exploration of various applications of graphene and its derivatives. Properties of pristine graphene include: (i) exceptionally high specific surface area of ∼2630 m^2^ g^–1^;^45^ (ii) better intrinsic carrier mobility of ∼2 × 1 0^3^ m^2^ v^–1^ s^–1^;^46^ (iii) superior mechanical strength with Young’ s modulus of ∼1. 0 Tpa;^47^ (iv) optical transmittance of ∼97.7%,^48^ and (v) very high thermal conductivity of ∼5000 Wm^–1^ K ^–1^.^49,50^ These exceptional properties make graphene stand out from other materials. Graphene behaves as a semimetal having zero band gap because its conduction and valence bands meet at the Dirac points. The unparalleled thermal conductivity of graphene can be attributed to its unique band structure that allows electrons to move at high speeds (about 1/300 the speed of light). These properties facilitate interaction with a wide range of molecules.

Chemically, graphene is sp^2^ hybridized, where each carbon forms covalent bond (σ bond) with three other carbon atoms forming a hexagonal array. This leaves one free electron (forming π-bond) per carbon atom. The presence of oxygenated functional group on the surface of GO, results in a hybrid structure of sp^2^ and sp^3^ hybridized carbon atoms.^64^ Proteins interact with sp^2^ hybridized graphene, sp^2^-sp^3^ hybridized GO and RGO and other variants through π – π electron interactions, hydrophilic or hydrophobic interactions, electrostatic and vdW interactions.^65−68^ It is utmost important to understand the concept of various interactions between graphene and proteins; to utilize them for various applications. Graphene and its derivatives have been extensively used in chemical/biosensors, as catalysts, in nanoelectronics, energy storage and nanomedicine applications such as drug and nucleic acid delivery, phototherapy and bioimaging.^43^

Surface functionalization of graphene (with epoxide, carboxyl, and hydroxyl) leads to the formation of variants like GO or RGO. The enriched surface functionalities provide GO and RGO with good aqueous solubility, unlike pristine graphene that show limited aqueous solubility.^69^ The surface functionalization widens the spectra of physical and chemical interactions. It provides plenty of reaction sites to link these 2D nanomaterials with small molecules, peptides, enzymes, proteins, polymers, bacteria, cells, nucleic acids, carbohydrates, other biomolecules, and organic/inorganic molecules through noncovalent or covalent binding.^37,70,71^ Interactions of graphene, GO, and rGO nanosheets with different proteins have been discussed further in the subsequent section of the article.

### 2.2 2D Transition Metal Dichalcogenides (TMDCs)

TMDCs are the 2D materials of type MX_2_, where M represents a transition metal atom (Mo, W, etc.) and X represents a chalcogen atom (S, Se, or Te), e.g., MoS_2_, WS_2_, WSe_2_, etc. It has a hexagonal lattice structure with 3-fold symmetry. Two layers of chalcogen sandwich a metal layer through covalent bonding, whereas weak vdW forces stacks the nanosheet layer (Figure 2B). The vdW forces is also responsible for interaction with other molecules. TMDCs are atomically thin semiconductors. After graphene, it is the most studied and researched class of 2D nanomaterial, because of its remarkable properties like direct band gap, absence of inversion center in monolayer crystal, high spin–orbit coupling, photon-conductance, etc.^72^

2D TMDCs (especially MoS_2_ and WS_2_) are known to possess great affinity toward biomolecules including proteins.^24^ TMDC interacts with proteins by virtue of hydrophobic, electrostatic interactions and through disulfide bond formation.^33^ However, some studies contradict the interaction through disulfide bond.^73^ Naturally, it lacks hydrogen bonding (hydrophilic bond), but upon introducing edge defects, it can show hydrophilic interactions too.^74^ Further, its tendency to form composites with various materials like noble metals, oxides, polymers, biomolecules, etc., makes it a promising candidate in wide variety of applications.^75^ The applications of TMDCs mostly include usage in label free biosensors, photothermal treatment, drug and gene delivery, bioimaging, transistors, photodetector, electrodes for Li-ion batteries, supercapacitors for energy storage, etc.^33,37,76−78^

### 2.3 2D Hexagonal Boron Nitride (hBN)

2D-hBN is an isomorph of graphene, having a similar hexagonal (honeycomb) lattice. The lattice has 0.145 nm spacing between alternating boron and nitrogen atoms. The presence of wide band gap in hBN renders it a white appearance which gives it an interesting name, “white graphene”.^79,80^Figure 2C shows the lattice structure details of hBN nanosheet.^81^ It has striking opto-electrical properties,^82^ mechanical strength,^83^ and chemical and thermal stability.^84,85^ It has strong oxidation resistance at high temperatures, making it a preferred coating material for metals preventing from oxidation and corrosion.^84^ hBN acts as an insulator and have high thermal conductivity, and its properties and functionalities are easily tunable^86^ following strategies like doping, hybridization, substitution, or functionalization with other materials. Implementation of hBN is perceived as reusable surface-enhanced Raman spectroscopy substrates,^85^ outstanding dielectric substrate for other 2D nanosheet and for electric field screening.^84^ hBN also possess excellent luminescence especially in the deep UV region which is helpful in bioimaging. In addition to these, hBN have also been used in detectors, photoelectric devices, and field effect transistors (FETs) and as nanofillers.^87^ Besides, studies show that noncovalent functionalization of hBN with polydopamine helped in reducing the interfacial thermal barrier and enhancing the thermal conductivity of BN-containing composites.^86,88,89^

### 2.4 2D-Xenes (Silicene, Germanene, Stanene, Borophene)

Monoelemental classes of 2D nanomaterials, comprising group III, IV, V, and VI atoms arranged in a honeycomb lattice (similar to graphene) are referred as 2D-Xenes (X = Si, Ge, Sn, B, P, Ga, Ge, and so on), for example, silicene, germanene, stanene, borophene, phosphorene, etc. Figure 2D represents different models of 2D-Xene nanosheets in side and top view. 2D-Xenes are synthesized by directly growing on a substrate, rather than through exfoliation from bulk material. They can exist as a trivial insulators, semiconductors or as semimetals.^90^ 2D-Xenes exhibit sp^2^–sp^3^ hybridization^69,91−93^ and have buckled hexagonal structures resulting in overlap of π-bonding p_*z*_ orbitals thereby giving rise to mixed sp^2^–sp^3^ hybridization.^94^ The sp^2^–sp^3^ hybridization and π–π* bonding of the 2D-Xene nanosheets allow interactions like hydrophobic interaction, vdW, etc., with other molecules including proteins, lipids, etc.

### 2.5 2D Metal Carbides and Nitrides (MXenes)

MXenes are emerging class of 2D transition metal carbides, carbo-nitrides or nitrides having the general formula M_*n*+1_X_*n*_ (*n* = 1–3). They are synthesized by exfoliating their three-dimensional (3D) MAX phases. MAX have a general formula of M_*n*+1_ AX_*n*_ (*n* = 1, 2, and 3), where M is an early d-block transition metals, A corresponds to main-group sp elements (predominantly IIIA or IVA), and X can be either or both C and N atoms, e.g., Ti_3_C_2_, Ti_2_C, etc. Figure 2E represents the side view of structure of Ti_2_C and Ti_3_C_2_. MXenes have hexagonal structure similar to graphene and are terminated with F, OH, and O based surface functional groups after exfoliation. OH/O termination is known to be most stable and creates a favorable environment for interaction with other materials. MXenes have large surface area with hydrophilic nature, allowing efficient adsorption and electrostatic interactions. The metallic conductivity of MXenes combined with the hydrophilic functional group terminated surfaces make them behave as “conductive clays”.^95^ MXenes possess high conductivity, good flexibility,^96^ and extremely high electromagnetic interference (EMI) shielding efficiency which protects the performance of electronic circuits and prevents partial or complete data loss. Potential applications of MXenes includes its usage in substitution of graphene in anode, as supercapacitors, storage devices, etc. Biological applications of MXenes include immobilization of enzymes and retaining their bioactivity and stability, as biosensor to detect various biomolecules, as antibacterial agents, for bioimaging and therapeutic applications such as phototherapy and drug delivery systems. MXene based materials provide excellent biocompatibility, stable interactions, and good aqueous solubility and are biodegradable.^97−103^

## 3 Nature of Interactions Between 2D Nanomaterials and Proteins

When a protein interacts with a nanomaterial there may be different kinds of forces involved. Hydrophobic, electrostatic, π–π stacking, vdW’s, and hydrogen bonding are some of the important noncovalent forces by virtue of which proteins may interact with nanosheets (Table 1), which can ultimately lead to adsorption of the proteins on the surface of the nanosheets. These interactions can unfold the protein so that it loses its physicochemical and structural–functional properties or can alter the structure of the nanosheet. Adsorption is particularly easier on the nanosheets than any other nanostructure because of their flat surface and planar structure which provides an increased surface area for interaction.^104−106^ Proteins consist of different amino acid residues having different hydrophobicities and nonuniform charge distributions. These charges vary with difference in their environmental conditions which in turn changes the nature of the interactive forces. Proteins consisting of charged functional groups tend to be more interactive and easily adsorb on to surfaces.^38,70^ The interactive forces between proteins and nanomaterials depend on several factors which includes surface charge, polarization, dipole moment, delocalization of π-orbitals, etc.

**Table 1:** Nature of Interactions That Occurs between Protein and 2D Nanomaterial

interaction type	amino acids involved	key features	refs
hydrophobic	Ala, Pro, Leu, Phe, Val, Ile, Gly, Met	•depend on the electron density and the geometry between the molecules.	(66,107−110)
		• proteins of high molecular weight form better interactions	
		• most common interactions between proteins and nanomaterials	
electrostatic	Lys, Arg, Glu, Asp	• depends entirely on charge of the surface functionalized groups on the nanomaterial and the charge of the amino acids	(100,111−115)
		• Zeta potential values act as indicators for binding efficiency.	
		• Interactions are dependent on the pH of the medium and the ionic strength of the buffer,k most commonly observed in enzyme-nanomaterial complexes	
π–π interactions	Phe, Trp, Tyr, His	• mostly observed between aromatic amino acids and nanomaterials.	(70,115−118)
		• occurs due to the huge delocalization of π-electrons on the surface of the nanomaterials arising due to the aromatic groups present on the surface	
		• There is a correlation between the polarizability of the aromatic ring and the strength of the interactions which indicates that with the increase in polarizability the strength of the interactions will also increase according to the trend His < Phe < Tyr < Trp.	
van der Waals (vdWs)	Lys, Arg, Glu, Asp	• short ranged in nature	(25,105,106,119−121)
		• similar to electrostatic interactions	
		• mostly observed between charged amino acids	
hydrogen bonding	Asn, Glu, His, Ser, Thr, Tyr	• takes place when there is a difference in electronegativity between two atoms	(36,122−125)
		• weak interactions	
		• type of dipole–dipole interaction	

Adsorption of the protein on the surface of the nanosheets can be absolutely spontaneous without altering any protein function else it can be detrimental to both the protein and the nanosheet.^126^ Apart from the different interactions concerning proteins and nanosheets, interactions between the solvent molecules and the nanosheets are also important to consider. Water being the most suitable solvent for biomolecules, the aqueous insolubility of the nanosheets is a huge obstacle. The solvent and nanosheets are also responsible in agonizing or antagonizing protein adsorption on nanosheet surfaces. Also, the nanomaterial surface may not be perfect. It may have defects or impurities and different atoms of the surface functionalities may interact in a different way when present in a different location. Thus, a thorough understanding of the nature of the interactive forces between a protein and a nanosheet is a necessity for any kind of biomedical applications of nanosheets.

### 3.1 Hydrophobic Interaction

These interactions are one of the most dominant interactions observed between proteins and nanomaterials. Hydrophobic interactions depend on the electron density and the geometry between the molecules.^38^ In general, graphene exhibits hydrophobic behavior due to the presence of aromatic carbon on its surface.^107^ On the other hand, GO exhibits a hydrophobic basal plane with hydrophilic edges. Although hydrophobic in nature, Xenes like phosphorene and silicenes show less hydrophobicity compared to graphene and its counterparts which was demonstrated through molecular dynamics simulations on the adsorption of HP35 on phosphorene.^127^ Elements like phosphorus, silicon, and germanium have a lower charge density than carbon which are directly proportional to the hydrophobicity of the molecules.^108^ This explains the lower hydrophobicity of Xenes in comparison to graphene. Also, phosphorene may be hydrophobic in nature but can be turned hydrophilic by oxidation.^128^ The hydrophobic nature of the nanosheets makes it insoluble in polar solvents, which is necessary in order to interact with proteins since most biological solvents are polar. Graphene also has strong dispersive forces in between the sheets.^129^ For solubilizing graphene, various noncovalent approaches have been employed. It has been observed that π-rich water-soluble polyelectrolytes can dissolve graphene. These polyelectrolytes form stable complexes with graphene with the help of π–π interactions and subsequently develop repulsive forces between the graphene-polyelectrolyte complexes which helps them to disperse.^130−132^ Recent studies have found hydrophobic interactions in graphene to be more effective than π–π interactions.^104,109^ In some cases, if the water-soluble conjugating molecule has a hydrophobic region, then it develops a strong hydrophobic interaction with graphene, which helps it dissolve. For example, strong hydrophobic interactions are observed between the graphene plates and the hydrophobic backbone of heparin, making graphene solubilize in aqueous media for further use. It has been observed that protein molecules mostly agglomerate on the hydrophobic regions and thus the presence of hydrophobic aromatic groups on the surfaces of the nanosheets enhances protein binding.^67^

### 3.2 Electrostatic Interaction

Electrostatic interactions depend entirely on the charge present on the interacting molecules. It utilizes the property of proteins to exhibit different charge at different pH conditions.^110,111^ Electrostatic interactions are useful in the assembly of nanocomposite and stabilization of nanomaterials. These interactions are mostly observed between GO and proteins. GO has a large surface area and is hydrophilic in nature due to the presence of epoxy, hydroxyl, and carbonyl functional groups which favor its interactions with other molecules.^112,113^ Graphene being hydrophobic electrostatic interactions are not that common. However, TMDCs show high conductivity, high charge density wave transitions and good biocompatibility.^100,114^ When exposed to an aqueous hydrophilic environment, stable aqueous dispersions of TMDC nanosheets can be obtained because of the presence of electrostatic forces. The main entity that governs the electrostatic forces is the surface charges which can be manipulated with the introduction of surfactants. When a surfactant is introduced in a solution containing TMDC nanosheets, they loosely bind to their surface and show characteristic positive or negative charge which not only helps in dispersing the nanosheets in the aqueous solution but also help in stabilization of the dispersion.^114^ It is to be noted that the electrostatic interactions may be complicated as they are dependent on the charge of the surface functionalized groups on the nanomaterial as well as on the interacting protein molecules. The charge status of these groups on the nanosheets change with the change in environmental factors like pH of the medium and its ionic strength. Also, the surface density of functional groups in 2D nanomaterials like GO varies with preparation procedure and storage conditions.^111^ Thus, any change in these factors would yield a completely different charge which would change the binding affinities as well as the overall nature of interactions.

### 3.3 π–π Interactions

π–π stacking exists between proteins and sp^2^ hybridized carbon nanomaterials as revealed by both computational and experimental studies. π–π interactions are generally found between proteins containing a large population of aromatic amino acids which specifically interact with nanosheets having a huge delocalized π-electrons on their surface like graphene and GO.^50,115−117^ Nanosheets like GO exhibits a very good display of π–π interactions due to its characteristic softness and flexibility because of which it can adapt its shape according to the aromatic amino acid thus aiding in stronger protein interactions.^118^ Alwarappan et al. observed the existence of strong π–π interactions between the individual hexagonal cells of the GO basal planes and glucose oxidase.^107^ When comparing with other short-ranged forces, the average π–π stacking distance is a bit higher than the vdW’s radius. It has been observed that the aromatic rings of the amino acids aligns in parallel with the plane of the substrates during π–π interactions.^119^ Graphene surfaces are mostly planar aromatic and form the best binding interactions with aromatic or amide groups in protein side chains. Quantum chemical calculations too revealed that binding of aromatic amino acids through π–π interactions are favored on a planar surfaces.^116^ Moreover, the surface of these nanomaterials does not have any form of curvature which favors these interactions.^105,119^ Recently conducted simulations reveal that proteins adsorbed on the graphene surface have lost their secondary or tertiary structure. Upon superposition of the protein structures before and after attachment to graphene shows that the main change in protein conformation was due to changes in the alpha-helices of the protein. These interactions are so strong that the aromatic amino acids lie flat on the surface of the nanomaterial. This phenomenon is responsible for deformation of the helices present in the proteins. There is a correlation between the polarizability of the aromatic ring and the strength of the interactions which indicates that with the increase in polarizability, the strength of the interactions will also increase according to the trend His < Phe < Tyr < Trp.^116^

### 3.4 van der Waals (vdW) Interactions

vdW’s interactions are a type of intermolecular interactions which are short-ranged and nonspecific in nature and mainly depend on the molecular surface area, electron charge density, and the dipole moment of the interacting molecule.^106^ These are weak forces which decrease drastically with the increase in distance between the interacting molecules.^25,120,121,133^ These forces come into being when neighboring molecules come so close to one another that they can influence each other’s surrounding electron clouds. Thus, they require a larger interacting area to establish a successful binding between a protein and nanomaterial. It has been observed that vdW forces have energy ranging from 0.5 to 1 kcal/mol and are short-range forces when compared to other molecular forces.^120^ The layered nanosheets themselves are interconnected using vdW forces as these forces play a very important role in stacking the nanosheets together. Although short-ranged in nature, vdW forces can quickly reassemble graphene into irreversible agglomerate or even assemble graphene sheet into graphite owing to its large surface area. During association of these nanosheets with different proteins, when two atoms come very close to one another, they repel each other. Consequently, this prevents any kind of imperfect fit between the molecules, in the presence of any kind of steric hindrance, as they are energetically very expensive. Thus, in case of determination of macromolecular specificity, vdW repulsive forces play a crucial role.^120^ When a protein molecule comes near the surface of a nanosheet, the charged amino acid residues like Lys, Arg, Glu, and Asp present in the protein play an important role in establishing the interactive force with the electron-rich regions of the nanosheets through vdW forces.

The vdW parameters are usually used to characterize interactions between nonpolar and π-electron rich molecules. Thus, graphene like nanosheets with a huge delocalization of π-electrons are a perfect fit for study of vdW forces. These forces also aid interactions between π-electrons and cations. It was observed through quantum mechanical studies that the vdW parameters enhances the interactions between both short-ranged cation−π interactions as well as long ranged dispersion interactions observed between graphene and ionic liquids.

In case of TMDCs like MoS_2_, both molybdenum and sulfur have huge surface charge densities which are more than carbon. Thus, vdW forces are stronger in TMDCs compared to graphene. For Xenes such as selenene, their structure seems to be composed of 0D atomic rings and 1D helical atomic chain. The Se–Se bond length is 2.4 Å within the rings and about 3.1 Å between nearby chains. Thus, vdW forces are the most dominating forces in these kinds of nanosheets.

### 3.5 Hydrogen Bonding

Hydrogen bonding is a kind of noncovalent interaction categorized under dipole–dipole interactions which is usually found between atoms with high electronegativity differences. Usually, this kind of bonding takes place in the presence of hydrogen atom linked to a very electronegative atom. The electron cloud of H atom gets decentralized due to attraction by the electronegative atom, thereby putting a partial positive charge on the hydrogen atom, which then attracts lone pair of electrons on other atoms thus forming hydrogen bonds. Amino acid residues such as Asn, Glu, His, Ser, Thr, and Tyr mostly participate in hydrogen bonding with nanosheet surfaces. The presence of these bonds have been traced in many nanosheets while interacting with proteins. Since proteins are organic molecules they have abundant −CH groups and amine groups which can easily form hydrogen bonds, for example they can interact with the Sulfur atom in the MoS_2_ nanosheets and often π-electrons to form π–S interactions.^134^ The most common hydrogen bonds found are NH---S and CH---S. They differ from the conventional OH---O hydrogen bonds, but they play a role in dispersion and electrostatic interactions in the molecular stabilization.

The hydrogen bonds play a major role in formation of the network of hexagonal BN nanosheets.^123^ The presence of hydrogen bonds in BN has been reported in the presence of surface functionalities such as those containing hydroxyl groups.

## 4 Analytical Methods for Evaluation of 2D Nanomaterial–Protein Interactions

When a 2D nanomaterial interacts with protein, several changes may occur in the protein structure and the nanosheet surface. The interactions can affect the surface chemistry, orientation, structure, and activity of both nanosheet and protein. These changes could either enhance the activity of proteins or disrupt its structure; it might be useful or detrimental. There might be strong binding, weak interaction, or the protein might not get adsorbed at all. The analysis of these changes allows us to understand the impact of interactions, so both qualitative analysis and quantification of the change is important. Various techniques are employed to get an insight of these interactions which help us to distinguish between the beneficial and nonbeneficial nanosheet–protein interactions. It also helps us to eliminate or find alternatives of the nonbeneficial interactions, know the exact location of interaction, nature of bond, possible change in protein structure, etc. In the following section, we provide a detailed discussion on the different analytical techniques that are regularly used to study the interactions of proteins with 2D materials (Table 2), by taking relevant examples from literature to explain them.^123−125^

**Table 2:** Analytical Techniques to Characterize Protein–2D Nanomaterial Interactions

	protein–nanosheet analyzed		
analytical technique	nanosheet	protein	analysis/interaction type
1. microscopic techniques			
1.1. atomic force microscopy (AFM)	GO	PEGylated albumin^135^	The thickness of the nanosheet before and after interaction with protein is measured; the change in thickness is indicative of the protein adsorption. The images give a 3D view of the surface topography for better visualization.
		BSA^136^	
		HRP and lysozyme^137^	
		antibody-IgG^138^	
		FBS^139^	
		BSA^140^	
		FBS^141^	
		BSA, Tf, IgG, and BFG^142^	
		peptide^143^	
		BSA and FBS^141^	
		BSA and FBS^144^	
	RGO	FBS^139^	
		BSA^140^	
	MoS_2_	HSA, Tf, Fg, and IgG^145^	
1.2 electron microscopy			
1.2.1 transmission electron microscopy (TEM)	GO	PEGylated albumin^135^	The electron micrograph observations talk about the morphology and topology of the nanosheet surface and protein adsorption on nanosheet surface can be visualized (TEM: only ultrathin dry samples; SEM: both dry and wet samples can be seen).
		β-lactoglobulin^146^	
		FBS^144^	
		BSA^136^	
		HRP^147^	
		plasma protein corona^148^	
	RGO	BSA and nanoparticle-BSA^140^	
		HRP^147^	
	MoS2	HSA, Tf, Fg, and IgG^145^	
	borophene and phosphorene	plasma protein corona^149^	
1.2.2 scanning electron microscopy (SEM)	GO	BSA^150^	
		Hb^151^	
		BSA^136^	
	MoS2	HSA, Tf, Fg, and IgG^145^	
	Ti_3_C_2_	tyrosinase^99^	
2. dynamic light scattering	GO	FBS^139^	analysis of mean particle size and size distribution profile; measuring the hydrodynamic size of the protein–nanosheet complex
		PEGylated albumin^135^	
		plasma protein corona^149^	
	RGO	FBS^139^	
	Ti_3_C_2_ and Ti_2_C	lysozyme^152^	
	borophene and phosphorene	plasma protein corona^149^	
3. zeta potential	GO	ubiquitin^153^	calculate the net charge on the surface of the protein–nanosheet complex and determine the presence of electrostatic interaction between them
		FBS^139^	
		plasma protein corona^149^	
	RGO	FBS^139^	
	MoS2	HSA, Tf, Fg, and IgG^145^	
	Ti_3_C_2_ and Ti_2_C	lysozyme^152^	
	borophene and phosphorene	plasma protein corona^149^	
4. spectroscopic techniques			
4.1 ultraviolet–visible spectroscopy (UV–vis spectroscopy)	GO	PEGylated albumin^135^	characterize the adsorption of protein on nanosheet surface as a function of change in spectral peak, impact of varied concentration of protein or nanosheet in complex formation, change in structure as compared to native forms, and as indicative of type of quenching occurring
		ubiquitin^153^	
		albumin, globulin, and Fg^148^	
		FBS^139^	
		BSA^136^	
		amino acids (phenylalanine, tyrosine and tryptophan), peptides (type 2 diabetes related human islet amyloid and Alzheimer’s disease related beta amyloid 1–40), and proteins (BSA and HSA)^154^	
		plasma protein corona^149^	
		bovine Hb^155^	
	RGO	FBS^139^	
	MoS_2_	anti-BSA with MOS_2_^156^	
	Ti_3_C_2_	hemoglobin^97^	
	borophene and phosphorene; black phosphorene	plasma protein^149^	
		BSA and BHb^157^	
4.2 fluorescence spectroscopy, quenching and FRET	GO	PEGylated albumin^135^	A change of characteristic fluorescence emission peak is indicative of protein adsorption on nanosheet; interaction can be measured by quenching of protein fluorescence by nanosheet and energy transfer mechanism (FRET) between the molecules.
		BSA^136^	
		albumin, globulin, and Fg^148^	
		FBS^139^	
		BSA	
		BSA^158^	
		amino acids (tryptophan and tyrosine), peptides (amyloid beta 1–40 and islet amyloid polypeptide), and proteins (BSA and HSA)^154^	
		chymotrypsin^159^	
		antibody-IgG^138^	
		bovine Hb^155^	
		trypsin^160^	
	RGO graphene	FBS^139^	
	MoS_2_ and WS_2_	BSA^158^	
	black phosphorene	BSA and BHb^157^	
4.3 infrared spectroscopy	GO	PEGylated albumin^135^	Identification and analysis of functional groups of the sample; the presence of characteristic peaks of the protein and nanosheet in the complex formed indicate their presence and the slight alteration suggests interaction between them and the resultant bond formation, while significant deviation/absence of protein peaks indicates disruption of protein structure.
		ovalbumin^161^	
	MoS_2_ (and WS_2_)	HSA, Tf, Fg, and IgG^145^	
		β-Gal-D308C, β-Glu, and HLD-A141C^162^	
	Ti_3_C_2_	tyrosinase^99^	
4.4 X-ray photoelectron spectroscopy (XPS)	GO	ubiquitin^153^	analyze the change in chemical bonds after protein adsorption on nanosheets and know the role of the hybridized state or the functional group in interaction
	RGO	BSA^140^	
	MoS_2_	HSA, Tf, fibrinogen (Fg), and IgG^145^	
4.5 circular dichroism (CD spectroscopy)	GO	PEGylated albumin^135^	Analyze the secondary structure and conformation of protein, and alteration in CD spectra is indicative of change in protein structure and denaturation.
		ubiquitin^153^	
		albumin, globulin, and Fg^148^	
		chymotrypsin^159^	
		ovalbumin^161^	
		BSA, Tf, IgG, and BFG	
		bovine Hb^155^	
	MoS_2_	HSA, Tf, fibrinogen (Fg), and IgG^145^	
	black phosphorus	BSA and BHb^157^	
5. isothermal titration calorimetry (ITC)	GO	ubiquitin^153^	quantify the interaction by measuring the disassociation constant, binding affinity; calculating the thermos-dynamic parameters.
	MoS_2_	HSA, Tf, fibrinogen (Fg), and IgG^145^	
6. molecular dynamic simulation	GO	HIV-1 integrase^163^	theoretical understanding of the protein–nanosheet interaction at the molecular level; determining the type of bonding taking place, the protein residues and nanosheet atoms involved in the interaction process.
		protein dimer^164^	
		chymotrypsin^165^	
		BSA, Tf, IgG, and BFG^142^	
		bovine Hb^155^	
		trypsin^160^	
	graphene	HP35^127^	
		insulin^166^	
	phosphorene	HP35^127^	
	BN	insulin^166^	
	MoS_2_	hybrid peptide of cecropin and melittin^167^	
	WS_2_	β-Gal-D308C, β-Glu, and HLD-A141C^77^	
		HP35^162^	

### 4.1 Microscopic Techniques

#### 4.1.1 Atomic Force Microscopy (AFM)

**Figure 3:**
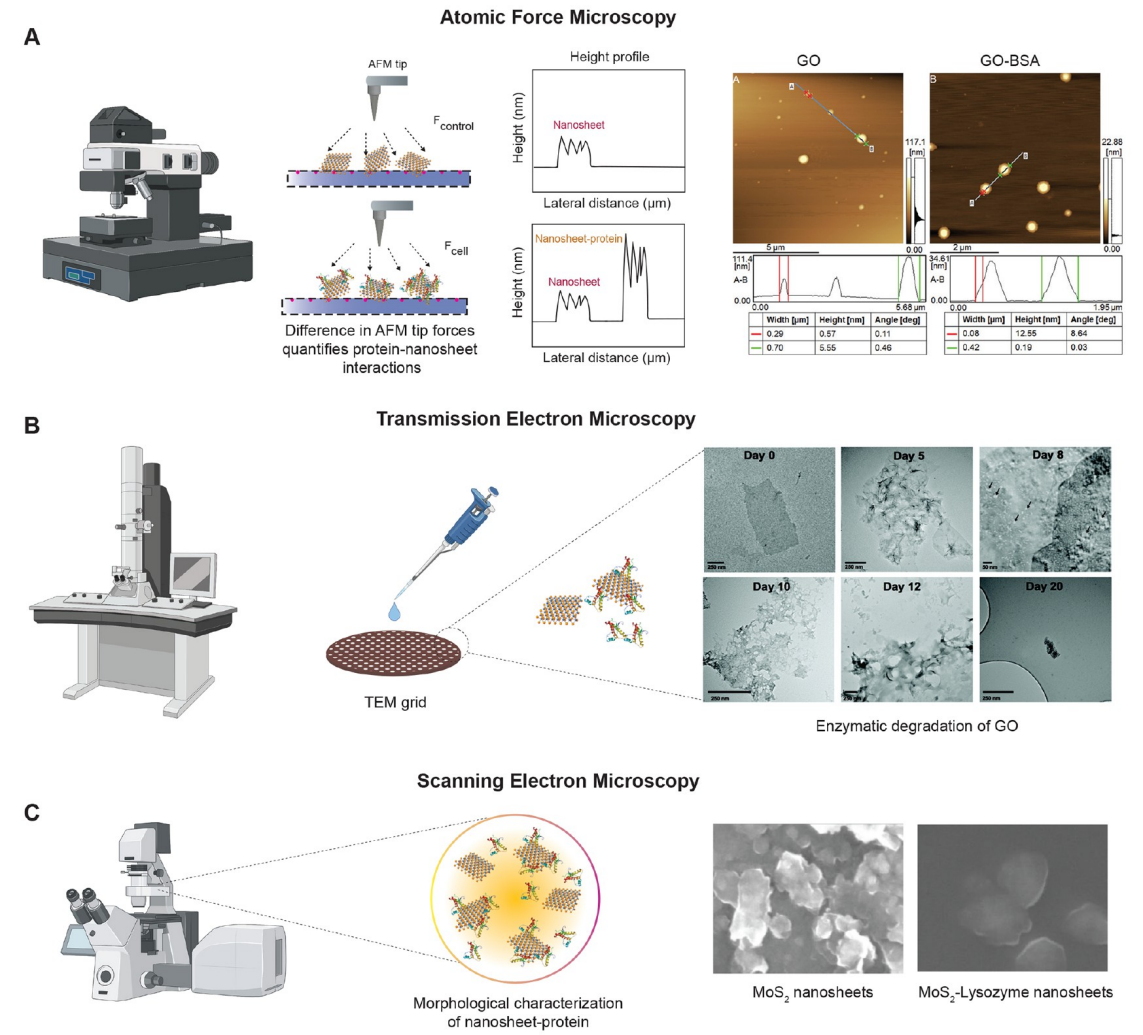
Microscopy tools to study 2D Nanomaterial–protein interactions. (A) Atomic force microscopy (AFM): Schematic showing AFM setup for probing nanosheet–protein interactions. Attachment of a biomolecule (protein) on the surface of 2D nanomaterials results in increase in the thickness of the nanosheets which can be monitored using AFM. AFM images of rGO obtained before and after incubating with FBS. Reproduced with permission from ref (139). Copyright 2015 American Chemical Society. (B) Transmission electron microscopy (TEM): TEM can be used to obtain high-resolution images of nanosheets before and after protein binding/interaction. The presence of an external system (protein) on the nanosheet surface can be revealed from the visual observation of the micrographs. TEM micrographs obtained on incubating GO with HRP and H_2_O_2_ for 0–20 days. Reproduced with permission from ref (147). Copyright 2011 American Chemical Society. (C) Scanning electron microscopy (SEM): SEM uses a focused beam of electrons that scans over the surface of a sample and creates the image of the surface of sample giving the topological information. SEM images of MoS_2_ NSs before and after lysozyme exfoliation. Reproduced with permission from ref (176). Copyright 2020 John Wiley and Sons.

AFM is a high-resolution, scanning probe microscopic technique which can provide information about surface topology of the nanomaterial/nanocomposite and local properties like surface thickness, height, friction, or magnetism based on the interaction between the sharp tip of cantilever probe and the material surface.^168,169^ In addition, it enables determination of the properties of adsorbed protein (elasticity, Young’s modulus, etc.), and the effects of interaction between protein and nanosheet (protein folding and unfolding, agglomeration, etc.). However, the depth of field of view in AFM is dependent on the cantilever shape-size and the piezoelectric probe moving it.^170−172^ AFM has been used widely to characterize the nanosheet and for verification of protein adsorption on the surface of nanosheets. The attachment of a biomolecule on the surface of 2D nanomaterials results in increase in the thickness of the nanosheet sample which can be clearly monitored using AFM (Figure 3A). The information regarding adsorption of blood plasma proteins, i.e., albumin, Fg, globulin on graphene, or the adsorption/immobilization of enzymes on various nanosheets has been obtained through AFM image analysis.^148^

Jokar et al.^135^ analyzed the surface thickness and shape of GO and PEG functionalized albumin- GO complex using AFM. The change in thickness of GO samples from thin (∼12 nm) to thick (∼50 nm) in the absence and presence of PEGylated-albumin, respectively, indicated the adsorption of PEGylated-albumin on the surface of GO. Similarly, another study Zhezhu et al.^136^ also determined the morphology of BSA and GO complex using AFM imaging. They observed increase in thickness of nanosheet after BSA adsorption. AFM was also used to analyze the enzyme immobilization potential of GO. Due to the presence of large surface area, GO showed adsorption of HRP, which was evident from the topology and thickness difference of the nanosheets as observed from AFM images.^137^ Liu et al.^140^ had studied the adsorption of BSA onto GO, RGO, and metal nanoparticle (NP)-conjugated RGOs by measuring the thickness of different samples. The AFM images showed increase in thickness from GO to NP-bound GO and BSA-GO complex to BSA-NP-GO complexes, indicating adsorption of protein on different GO-based nanosheets. Yu Chong et al.^142^ studied the interaction of GO with abundant plasma proteins, i.e., BSA, BFG, Ig, and Tf. They visualized the adsorption of plasma proteins on GO by observing the AFM images, which demonstrated a characteristic change in its thickness. Wei et al.^139^ used AFM images to examine the change in surface morphology of GO and rGO on interaction with FBS. They observed thickness change from ∼1 nm of pristine GO to ∼2 nm for FBS-bound GO monolayer. However, rGO was seen as particles instead of sheet-like structure and FBS coating around the rGO particles showed altered height-width profile. Figure 3A shows change in height of rGO from 0.57 to 12.55 nm (red label), indicating adsorption of FBS. The adsorption of peptide GAMHLPWHMGTL (a dodecamer peptide) on the planar surface of graphene via π–π interactions was demonstrated by difference in height thickness before and after adsorption using AFM by Katoch et al.^143^ In a GO-based biosensing platform, AFM was employed by Haung et al.^138^ to determine the adsorption of antibody IgG on GO surface. The AFM showed the change of thickness of sheets from 1 nm for naïve GO to 10 nm for IgG-bound GO. Duan et al.^141^ and Hu et al.^144^ did a similar study and examined the thickness of GO in FBS (serum)-free medium and in varying concentration of FBS-containing medium. The AFM images revealed the thickness of GO in FBS free media was 1.5 nm, while the thickness varied from 2.5 to 4.5 nm with increase in FBS concentration (1–10%). All these results demonstrate that increase in thickness of nanosheets on interaction with protein indicates adsorption of proteins on the nanosheet surface and forms a characteristic feature of protein–2D nanomaterial interaction which can be easily monitored using AFM analysis.

#### 4.1.2 Electron Microscopy

Electron microscopy (EM) is used to obtain high-resolution images by utilizing a beam of high accelerating electrons as source of illumination. Magnification of 10,000,000 times can be achieved with a resolution of 50 pm.^173^ It is thus widely used to obtain high-resolution images of various biological and nonbiological samples like cells, tissues, nanoparticles, etc. EM is usually used to characterize the structure, topology, morphology, and or composition of the sample. EM can also be used to visualize the adsorption of proteins on nanosheet by comparing the images of native nanosheet before and after interaction with proteins. EM is broadly classified into two main types: (1) transmission EM (TEM) and (2) scanning EM (SEM). TEM is analogous to conventional compound light microscope, and the image in TEM is formed by the transmitted electron from a thin section of sample. In SEM the image is created by the secondary electron generated from the specimen when impinged with a beam of electron. SEM and TEM are most widely used EM techniques and can be employed to reveal information about the protein and 2D nanomaterial interactions.

In TEM, the electrons are focused on an ultrathin sample and the electrons transmitted through the specimen are used to project the image. TEM is employed to obtain high-resolution images using electrons accelerated between 40–200 kV potential. However, TEM analysis needs an ultrathin dried sample.^174^ The TEM micrographs have been extensively used to obtain and compare high-resolution images of pristine nanosheet and after protein adsorption (Figure 3B). The presence of external system (protein) on the nanosheet surface can be revealed from the visual observation of the micrographs. In addition to it, TEM has been widely employed to observe interaction of protein–nanosheet complexes with cell and cell membrane. TEM micrographs revealed the difference in morphology of pristine GO nanosheet structure and that of nanosheet surface after interacting with BSA.^150^ Jokar et al.^135^ analyzed the surface of GO and PEG functionalized albumin (APC) using TEM. The resultant TEM micrograph showed that the surface modification of GO nanosheet occurred in the presence of APC; the surface changes from thin to a thicker layer and agglomeration occurs due to adsorption of APC on GO forming APC-GO complex. In addition, Kotchey et al.^147^ studied the oxidation of GO and RGO by HRP using TEM micrographs taken on different days (0–20 days) in a periodic fashion. The TEM images (Figure 3B) revealed that HRP is capable of oxidizing GO. A typical flat-sheet characteristic of GO was observed on the zeroth day, followed by some wrinkling on the fifth day and the formation of holes on the eighth day which continued to enlarge with passing days. By 20th day, small oxidized GO flakes were seen. In contrast, TEM micrograph of the RGO study showed no further oxidation on interaction with HRP, thus indicating HRP oxidizes GO but not RGO. In a similar study to understand mitigation of cytotoxicity of nanosheets using protein corona, Hu et al.^141^ observed and compared the TEM images to know the impact of pristine GO and FBS coated GO on the cell membrane integrity and found that FBS coated GO showed reduced disintegration of cell membrane as compared to pristine GO nanosheets. Han et al.^149^ investigated the internalization of borophene nanosheets (B NS) and plasma protein bound borophene nanosheet (B NS-corona) using TEM and its impact on cell surface. It was observed from TEM images that both bare B NS and B NS-corona were phagocytosed via endocytosis into the macrophages and accumulated as irregular aggregates in the lysosome dispersed in the entire cytoplasm of the cell. Thus, TEM plays a major role for visualization of ultrathin protein–nanosheet complex and their cellular interactions.

SEM uses a focused beam of electrons that scans over the surface of a sample and interacts with the electrons in the sample to produce signals in the form of back-scattered electron (BSE), secondary electrons, X-rays, and light rays (cathodoluminescence). Standard SEM usually uses BSE and secondary electrons for image formation; having resolution of 1 nm and magnification of the sample up to 500000 times. Electrons create the image of the surface of sample giving the topological information. Besides, it can be used for 3D view of the exteriors of object using photogrammetry, photometric stereo, and inverse reconstruction.^175^ SEM has been used widely to observe the presence of protein on the nanosheet surfaces (Figure 3C). Baimanov et al.^145^ visualized the images and observed the size distribution of MoS_2_ nanosheet (NSs) upon interaction with four abundant proteins of blood plasma, i.e., HSA, Tf, Fg, and IgG using SEM. The SEM data analysis revealed increase in length-width distribution profile of the NSs from 154–137 nm to 160–141 nm for NSs-HSA, 196–171 nm for NSs-Tf, 324–276 nm for NSs-Fg, and 317–236 nm for IgG, which clearly demonstrated protein corona formation. Hence, EM provides a fair idea of protein adsorption on nanosheet by allowing visualization of the nanosheet surface.

### 4.2 Scattering Techniques

#### 4.2.1 Dynamic Light Scattering (DLS)

DLS also referred to as photon correlation spectroscopy (PCS) is a scattering technique that helps in estimation of hydrodynamic size and size distribution profile of particles. DLS measurement is attributed to Brownian motion of the particles, resulting in time-dependent fluctuations in the scattering intensity.^177^ Small particles collide with each other in a solution or suspension resulting in energy transfer among particles. The energy transferred is more or less constant, further inducing particle movement. Thus, it results in faster movement of smaller particles as compared to larger ones.^178^

**Figure 4:**
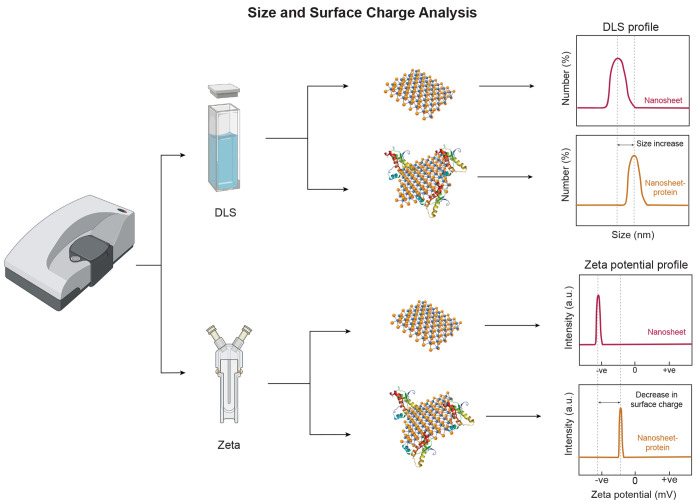
Scattering techniques to study 2D nanomaterial–protein interaction. Dynamic light scattering or DLS yields valuable information about size and size distribution profile of small particles in solution or suspensions. Comparison of DLS spectra of pristine nanosheets and protein–nanosheet complex shows an increase in size of the complex due to the adsorption of proteins on nanosheet surface. Zeta potential measures the charge present on the surface of particles. Interaction of nanosheets with proteins results in a change of the net surface charge of the protein–nanosheet complex which can be detected using zeta potential analysis.

DLS yields valuable information like size and size distribution profile of small particles in solution or suspensions. It also helps in determining the diffusion coefficient. The size distribution of the particles is represented as polydispersity index (PDI). The comparison of multiangle DLS spectra of pristine nanosheet and free proteins with that of protein–nanosheet complex is another way of studying the nanomaterial–protein interactions. As the protein gets adsorbed on the nanosheet surface, there will be an increase in size of the nanosheets, resulting in lowering of their movement speed (Figure 4). This provides information about change in mean hydrodynamic size, size distribution profile, and the diffusion coefficient.

DLS has been extensively used to measure the hydrodynamic size of nanosheets and protein–nanosheet complexes. Wei et al.^139^ observed the change in hydrodynamic size of GO and RGO after adsorption of FBS on their surface. The DLS study showed significant increase in size of the nanosheets after protein adsorption; however, the results were not in agreement with the AFM and zeta potential measurements, indicating that the increase in size is not just due to protein adsorption but also due to aggregation around the nanosheets. Rozmysłowska-Wojciechowska et al.^152^ studied the comparative surface property changes in MXenes Ti_2_C and Ti_3_C_2_ on interaction with lysozyme. Time-resolved DLS technique revealed that the concentration of lysozyme have a significant impact on the hydrodynamic size of the nanosheets. Ti_3_C_2_ displayed a maximum hydrodynamic diameter of 1059 nm at 1:3 ratio (Ti_3_C_2_/Lysozyme), while Ti_2_C displayed a maximum diameter of 648 nm at 1:2 ratio (Ti_2_C/Lysozyme). Similarly, DLS study revealed that the hydrodynamic size of borophene nanosheet (B NS) increased from 318.6 ± 2.3 nm to 393.5 ± 5.9 nm upon interaction with plasma protein indicating the adsorption of proteins on to B NS surface leading to the formation of protein corona around B NS.^149^

#### 4.2.2 Zeta Potential (ζ-Potential)

ζ-Potential is the measure of magnitude of charge present on the surface of particles. Both nanosheets and proteins have certain charge on their surfaces which help in their interaction and results in change in the zeta potential after complex formation (Figure 4). It gives information about the net surface charge on the protein–nanosheet complex which is also responsible for stability estimation of the protein–nanosheet complex.^179^ The measurement also gives an idea of dispersion, aggregation, or flocculation state and thus can be applied to study protein–nanosheet interaction.^153,180^ The higher value of zeta potential above ±60 mV indicates excellent stability, whereas a value below ±30 mV shows the tendency to aggregate.^181^ It has to be noted here that the zeta potential values are dependent on the pH and ionic strength of the dispersing medium which can affect the net charge present either on 2D nanomaterials or on proteins and can thus influence interactions between the two.

The measurements of zeta potential are widely used to check the aggregation profile of protein adsorbed nanosheets. Mostly the adsorption of proteins onto the surface of 2D nanomaterials decreases the zeta potential of the bare 2D nanomaterials (Figure 4, Table 3) thereby reducing the aqueous dispersibility of the material. Han et al.^149^ compared the interaction of plasma proteins with three nanosheets, i.e., borophene (B NS), phosphorene (BP NS), and graphene (GR NS). Zeta potential values of the nanosheets showed the presence of negative charge on all the three nanosheets; however, on interaction with plasma proteins, the NSs showed reduction in their negative value. Ti_3_C_2_ and Ti_2_C nanosheets have negative zeta potential but when equal concentration of positively charged lysozyme protein was introduced, a drastic shift to positive value was observed. This change in zeta potential value indicates that electrostatic interaction occurred between the lysozyme and MXene nanosheets. The study on the aggregation behavior of the nanosheets upon interaction with proteins is important while considering biological applications of these nanosheets as it may affect the biocompatibility and fate of these nanosheets and thereby directly influence the efficiency of the intended application.

**Table 3:** Change in Zeta Potential of 2D Nanomaterials before and after Interacting with Proteins

		zeta potential of 2D nanomaterial (mV)		
2D nanomaterial	protein	before interaction	after interaction	references
GO	FBS	–33	–15	(139)
RGO (20 min)		–14	–10	
RGO (120 min)		–15	–13	
MoS_2_	HSA	–36.8	–19.3	(145)
	Tf		–9.2	
	Fg		–7.2	
	IgG		+10.6	
GO (in water)	BSA	–48.8	–40.3	(150)
GO (in cell culture media)		–10.4	–9.98	
borophene	blood plasma proteins	–24	–12.5	(149)
graphene		–19	–8	
phosphorene		–18	–7	
GO	BSA	–33.3	–18.3	(182)
Ti_3_C_2_	lysozyme	–24	+0.5	(152)
Ti_2_C		–22	+4	

### 4.3 Spectroscopic Techniques

#### 4.3.1 Ultraviolet and Visible Light Spectroscopy (UV–Vis Spectroscopy)

The interaction between protein and 2D nanomaterial can lead to change in absorption spectra of either protein or pristine nanosheet. UV–vis absorption spectroscopy works on the principle of excitation of electrons present in the ground state to a higher energy excited state by absorbing ultraviolet or visible light. This absorption process causes the π electrons or nonbonding electrons (n-electrons) to get excited to higher antibonding molecular orbitals with the possibility of π–π*, n−π*, σ–σ*, and n−σ* transitions.^183−185^

**Figure 5:**
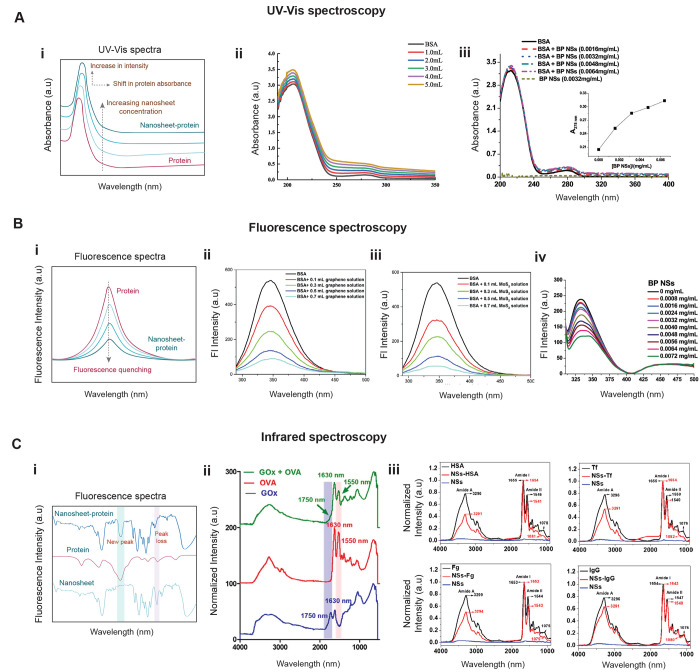
Spectroscopy tools to study 2D nanomaterial–protein interaction. (A) UV–vis spectroscopy: (i) Schematic representation showing change in UV–vis spectra of proteins upon interaction with nanosheets. Interaction of proteins with increasing concentrations of nanosheets results in a shift in protein absorbance along with an increase in absorbance intensity. (ii) Effect of increasing GO concentration on the absorbance of BSA. Reproduced with permission from ref (136). Copyright 2019 Elsevier. (iii) Effect of BP NPs on the absorbance of BSA. Reproduced with permission from ref (157). Copyright 2020 Elsevier. (B) Fluorescence spectroscopy: (i) Schematic representation showing change in fluorescence spectra of proteins upon interaction with nanosheets. Interaction of proteins with increasing concentrations of nanosheets results in fluorescence quenching of proteins in a concentration-dependent manner. Fluorescence spectra of BSA in absence and presence of different quantities of (ii) graphene and (iii) MoS_2_. Reproduced with permission from ref (158). Copyright 2017 Royal Society of Chemistry. (iv) Fluorescence quenching of BHb by BP NPs. Reproduced with permission from ref (157). Copyright 2020 Elsevier. (C) Infrared spectroscopy: (i) Schematic representation showing change in IR spectra of protein–nanosheet complex. Interaction of proteins with nanosheets can lead to formation of new bonds which can appear in IR spectra as new peaks or disappearance of an existing peak. (ii) ATR-FTIR spectra of GOx, OVA, and GOx-OVA after removal of unbound OVA. Reproduced with permission from ref (161). Copyright 2016 American Chemical Society. (iii) The FTIR spectra of HSA, Fg, Tf, and IgG before and after interaction with NSs. Reproduced with permission from ref (145). Copyright 2020 American Chemical Society.

Proteins have three types of chromophores that are relevant for UV/vis spectroscopic measurements: peptide bonds (amide bond) having intense peak at 190 nm due to the π–π* and the weaker one at 210–220 nm arising from n−π*transitions; certain amino acid side chains, mainly tryptophan (strong peak at 280 nm), phenylalanine (weak peak at 257 nm), and tyrosine (intense peak at 274 nm); and certain prosthetic groups (having peaks near ∼420, 450, and 480 nm) and coenzymes, e.g., porphyrin groups such as in heme (having transition near ∼400 nm).^184^ The interaction of proteins with nanosheets may change the spectral features in the absorption spectrum of proteins and thus these signatures can act as an indicator for the interaction between the protein and nanosheets. Zhezhu et al.^136^ utilized the technique of UV–vis spectroscopy to study the interaction of BSA with GO nanosheets. They examined the effect of increasing concentration of GO on the absorption spectra of BSA and found that with increasing concentration of GO, there was a red shift in the absorption maxima (λ_max_) of BSA thereby indicating strong interaction between BSA and GO (Figure 5A-ii). Zhang et al.^157^ observed the interaction of BSA (Figure 5A-iii) and BHb with black phosphorene nanosheet (BP NSs), where they used UV–vis spectra to study the change in protein adsorption in the presence of phosphorene as compared to free proteins. Both the proteins have characteristic peak at 212 and 278 nm, corresponding to the n−π* transition of C=O and the π–π* transitions of the aromatic residues of the proteins. An additional peak (soret band) was observed in BHb at 410 nm owing to d−π* transition between heme complex and the iron component of BHb. In the presence of BP NSs, the characteristic absorption peak 278 nm of both the proteins showed a slight increase in absorption wavelength; however, no such change was observed for the Soret band at 410 nm indicating heme group did not get affected. The change in λ_max_ implied interaction between proteins and nanosheet have occurred, and the nanosheet had enhanced the extension of the peptide chain and altered the hydrophobicity around aromatic residues of protein. Han et al.^149^ analyzed the adsorption of plasma proteins on borophene nanosheet (B NS). UV–vis spectra showed the presence of additional peaks in the protein–B NS complex in comparison to native B NS spectra indicating the interaction and adsorption of protein on the borophene nanosheet. Thus, it can be stated that either the presence of UV–vis light absorption peak corresponding to proteins in the protein–nanosheet complex or the alteration in absorption spectra of proteins on interaction with the nanosheets helps to demonstrate the interaction between the proteins and the nanosheets.

#### 4.3.2 Fluorescence Spectroscopy

Analysis of relative intensities and wavelength of emitted light through fluorescence spectroscopy can provide useful information to study the interactions between proteins and nanosheets.^186^ The change in fluorescence intensity is utilized to analyze 2D nanomaterial and protein interactions. The fluorophore could be intrinsically present, or material could be extrinsically labeled with a fluorophore. An advantage of using this technique is that protein possess intrinsic fluorophores due to the presence of certain amino acids, i.e., tryptophan, tyrosine, and phenylalanine. Either or both, the protein or the nanosheet could also be labeled with a fluorophore for better understanding of the interaction between them. Mechanisms like quenching and fluorescence resonance energy transfer (FRET) enable to understand the molecular interactions between a 2D material and a protein.^185^

Quenching is the process that leads to a decrease in fluorescence intensity of a fluorophore. There are many molecular interactions that results in quenching of the fluorophore. Proteins usually possess intrinsic fluorescence which is quenched by nanosheets on its adsorption (Figure 5B-i). Thus, the quenching of protein’s fluorescence indicates proximity/binding of protein to the nanosheet.^187^ On the other hand, FRET is based on the phenomenon of radiation-less transfer of energy from a donor (fluorophore) to a acceptor (chromophore) through long-range dipole–dipole interactions.^188^ The spectral overlap between the donor’s emission and acceptor’s absorption greatly influences the sensitivity of the process. Also, the separation distance between the donor and the acceptor which typically lies in the range of 1–10 nm affects the energy transfer process.^189^ This makes FRET an extremely useful technique to study molecular interactions.^190^

Li et al.^154^ analyzed the interaction of GO with amino acids, peptides and proteins. A strong decrement in the fluorescence intensity of tryptophan on interaction with GO (after eliminating the inner filter effect) indicated adsorption of Trp or Tyr via π–π interaction or hydrophobic interaction on GO. Quenching effect was seen to be directly proportional to the concentration of GO and independent of temperature. The effect of pH on change in fluorescence intensity was also studied to verify electrostatic interactions. The quenching efficiency was found to decrease at basic pH (pH ∼ 9) as compared to a pH of 5.6 which indicated presence of electrostatic interaction, as GO and Trp were both negatively charged. A decrease in quenching efficiency in the presence of Pluronic F127 (triblock copolymer, that shows strong hydrophobic interaction with graphene^191^ and GO)^192^ indicated the strong hydrophobic interaction of GO sheets with Trp and Try. The fluorescence of Tyr in peptides Aβ40 or hIAPP was quenched on interaction with GO, indicating presence of noncovalent interactions. Similarly, strong quenching was also seen on interaction of BSA and HSA with GO. The difference in quenching efficiency between different peptides or proteins can arise due to the variation in structural configuration. This study on the interaction of peptides and proteins with GO showed good quenching efficiency of GO and thus indicated strong interaction.

Arun et al.^158^ examined the interaction of BSA with graphene, MoS_2_ and WS_2_ in a concentration-dependent manner (0.1–0.7 mL of nanosheet). Figure 5B-ii,iii shows that the fluorescence intensity of BSA decreased with increasing concentration of 2D nanomaterials. A similar pattern was observed with all the three nanosheets. The average fluorescence lifetime (FL) of natural BSA is around 6.41 ns, which varied between the range of 6.08–5.55 ns with increasing graphene concentration, between 6.39 and 5.62 ns with increasing MoS_2_ concentration and for WS_2_, the range of 6.26–5.44 ns was observed. They also calculated energy transfer efficiency from BSA to individual nanosheets using steady state fluorescence spectra and observed the energy transfer efficiency to be in the range of 27–89% for different nanosheet at different concentrations. Similarly, the interaction of BHb^155^ and PEG-functionalized Albumin^135^ with GO resulted in quenching of fluorescence of the respective proteins. The nature of interaction was considered mainly to be vdW, hydrophobic, and electrostatic binding. Further, Zhezhu et al.^136^ and Mu et al.^150^ studied separately the interaction of BSA with GO. Both the studies showed that GO quenches the intrinsic fluorescence of BSA, indicating possible interaction between both the molecules.

Additionally, FRET based assays are an important component of 2D nanosheet based biosensors and has been widely employed for the same. Zhang et al.^193^ designed a biosensor using a dye-labeled peptide (donor) and GO (quencher) for protease monitoring. Peptide (donor) interacts with basal plane of GO via aromatic and hydrophobic residues forming π–π stacking and forms electrostatic bonds with charged and polar residues present at the edges. The interaction between both led to FRET and the fluorescence of peptide got quenched. In the presence of desired protease, dye-labeled peptide dissociated from GO nanosheet (due to hydrolysis), and thus the fluorescence was recovered. The extent of regained fluorescence intensity indicated the presence of protease and its concentration.

Wei et al.^139^ examined the interaction of FBS with GO and RGO (of varying degree) by measuring the quenching efficiency of the nanosheets. The results showed that quenching efficiency increased with increase in concentration of nanosheets, while quenching decreased with increase in reduction of GO to RGO. GO showed maximum quenching of FBS followed by RGO (20 min reduction treatment time). RGO nanosheets that were subjected to 120 min reduction time showed least quenching as compared to the other two. The overall results indicated that FBS showed the highest binding affinity with GO, followed by RGO (20 min) and least binding affinity with RGO (120 min) which is due to decrease in the hydrophilicity on increased reduction of GO/RGO.

De et al.^159^ investigated the change in secondary structure of chymotrypsin (ChT) after adsorption on GO surface using fluorescence spectroscopy. The results represented that the free ChT has a characteristic fluorescence emission peak at 334 nm, and for the denatured ChT the peak is red-shifted to 352 nm which is attributed to the hydrophobic environment or strong electrostatic interactions of Trp residues with the aqueous environment. The ChT-GO complex showed a fluorescence emission peak similar to that of natural ChT and over a time period of 24 h, both the control (free ChT) and ChT-GO showed only a slight red-shift that could be attributed to protein aging and not complexation with GO. This showed that secondary structure of protein (ChT) remained intact even after adsorption on GO nanosheet surface. Yao et al.^160^ used fluorescence spectrometry to not only analyze the interaction between protein (trypsin) and GO, but also the impact of thermal treatment (70°) of the complex and PEGylation of GO sheet on the stability of the secondary structure of protein in trypsin-GO complex. Fluorescence spectra showed that the characteristic fluorescence emission peak of trypsin at 343 nm got red-shifted to 358 nm on thermal denaturation. The fluorescence spectra of trypsin incubated with GO and GO-PEG-1 showed similar emission peak before and after thermal treatment indicating that the nanosheets can efficiently protect trypsin’s structure during thermal treatment. However, the adsorption of trypsin on a highly PEGylated GO nanosheet, i.e., GO-PEG-2.5 and GO-PEG-5, do not protect protein from denaturation at higher temperature. A red-shift in the emission spectra of trypsin was seen upon thermal treatment as compared to that at room temperature in case of high PEGylated GO.

Zhang et al.^157^ studied the interaction of Xenes nanosheet phosphorene (BP NSs) with two common bovine proteins, albumin (BSA), and BHb. They observed the quenching of the fluorescence (from Try at 295 nm) of proteins in the presence of BP NSs (Figure 5B-iv). They further revealed that BP NSs was able to quench 25.7% fluorescence of BSA and 48.5% of the BHb; this difference suggests that both the proteins have different binding affinity for the nanosheet. Hydrophobic interaction was thought to be responsible for interaction of BP NSs with BSA and BHb.

#### 4.3.3 Infrared Spectroscopy (IR Spectroscopy)

IR spectroscopy is a linear vibrational spectroscopy that measures the stretching and bending vibrations of the bonds present in molecules.^194^ The demonstration of interaction is done by examining spectral bands, which allows molecular characterization, identification, and functional group analysis of the sample. This technique can be used to analyze the interaction between 2D nanomaterials and proteins, through the identification of functional groups and matching it with the fingerprint regions. The IR spectra of the pristine nanosheet and protein are compared with the protein–nanosheet complex after interaction to identify the presence of additional peaks indicating new bond formation and absence of existing peaks indicating deletion/alteration of original bond present in the protein or nanosheet (Figure 5C-i). The FTIR technique can be used in conjugation with other (spectroscopic or microscopic) techniques to provide better understanding of the molecular interactions.

Li et al.^161^ investigated the change in secondary structure of ovalbumin on adsorption onto GO using FTIR. The FTIR spectra of OVA-GO complex, Figure 5C-ii, showed peaks of both free OVA and GO indicating interaction between both, but the absence/reduced intensity of some peaks of OVA in complex indicated denaturation of the protein structure, which was further confirmed using CD spectra. The change in secondary structure of blood plasma proteins on interaction and adsorption on the TMDC based MoS_2_ nanosheets was examined by Baimanov et al.^145^ using CD and FTIR spectroscopy. Figure 5C-iii represents a slight shift in the peak position of the protein amide A band of the blood proteins upon interaction with MoS_2_ nanosheet. This indicates interaction between C–N group of proteins and the NSs leading to stretching of the N–H peak. Further, peak shifts for C=O stretching, C–O stretching, and C–N stretching along with N–H bending were also observed. The changes in both the amide I and II bands is indicative of secondary structure and conformational change in protein arising from interactions with nanosheets. Thus, FTIR, indicates the impact of 2D nanomaterials–proteins interactions on the secondary structure of proteins.

#### 4.3.4 X-ray Photoelectron Spectroscopy (XPS)

XPS is a surface analysis technique that examines core levels of elements using soft X-ray (200–2000 eV) radiations. Every element has a characteristic binding energy associated with a particular atomic orbital. Each and every element, except hydrogen and helium (insensitive to XPS), shows a distinct set of characteristic peaks in XPS. The chemical state of the atom can affect the shape and binding energy of the peak. Thus, XPS also helps in knowing the chemical bonding. The advantages of XPS include its chemical sensitivity and its suitability for solid surface investigation.^195^ XPS helps in determining the empirical formula, elemental composition, and chemical and electronic state of the elements within the material.^196^ XPS also helps in measuring what elements are bounded to the subject matter. This property of XPS is used to characterize the interaction between protein and 2D nanomaterials. It has a high sensitivity of few nanometers and can identity and quantify elements present within 1–12 nm of the nanosheet surface. Paynter et al.^197^ showed that XPS can be used to determine the thickness and coverage of protein adsorption on nanosheet surface. It provides information that whether the protein is absorbed in patches or in continuous form in real time. In the study of MoS_2_ NSs–blood protein complexes, the characterization of nanosheet–protein complex was done using XPS by Didar et al.^145^ which showed strong shift of the Mo 3d peak from 5/2 to 3/2 indicated the adsorption of two proteins, IgG and Fg, on the MoS_2_ nanosheet. Thus, the change in peaks of XPS spectra can be used to understand the interaction between protein and 2D nanomaterials.

#### 4.3.5 Circular Dichroism (CD Spectroscopy)

**Figure 6:**
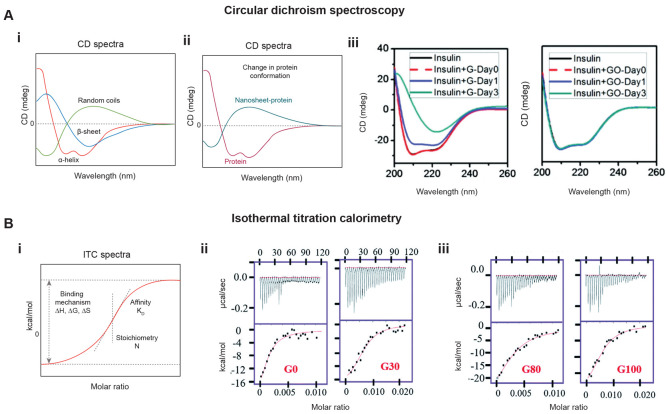
(A) Circular dichroism spectroscopy as an aid to study nanosheet–protein interactions. CD spectroscopy helps to deduce the conformation and secondary structure of proteins. Schematic representation of a CD spectra for (i) normal protein and (ii) nanosheet–protein complex showing the different secondary structures. Comparison between the two CD spectra reveals a change in the secondary structure of the protein upon interacting with the surface of nanosheet. (iii) Far-UV absorption CD spectra of insulin in PBS in the presence of G and GO. Reproduced with permission from ref (200). Copyright 2011 American Chemical Society. (B) Isothermal titration calorimetry as a tool to study nanosheet–protein interactions. (i) ITC allows thermodynamic characterization of nanosheet–protein interaction by providing information about stoichiometry, binding affinity, dissociation constant, association constant, and binding enthalpy. (ii, iii) ITC studies of human ubiquitin with varied GO samples. Reproduced with permission from ref (153). Copyright 2020 Royal Society of Chemistry.

Molecules with molecular asymmetry show differential absorption of right- and left-handed circularly polarized light. This property of differential light absorption is referred to as circular dichroism (CD), which makes it a robust analytical technique to determine the conformation and secondary structure of proteins. In a typical CD spectrum, the peptide bond present in the protein backbone gives a characteristic band in the far-UV region (178–260 nm), whereas aromatic amino acid side chains and prosthetic groups of the protein shows CD bands in the near UV (350–260 nm) and visible regions (400–700 nm). Secondary structure of the proteins, like α-helix, β-sheet, and random coil have their characteristic CD spectra in UV/vis region.^198^ CD spectra could partially help in determining the change in the structural conformation of the protein on interacting with a nanosheet. If the protein–nanosheet complex shows spectra similar to the native protein, then it could be inferred that protein structure is not disrupted; in case of altered spectra, there may be change in protein conformation which may affect the function of the protein (Figure 6A-i,ii).^140,199^

Chong et al.^142^ studied interaction of bovine fibrinogen (BFG), Ig, Tf, and BSA with graphene based nanosheets. The CD spectra of BSA and Tf showed drastic structural rearrangement from reduced α-helical to enhanced β-sheet just after 5 min of incubation with GO, however no further significant alteration was observed after 60 min. While the CD spectra of Ig and BFG showed change as a function of time, i.e., the altered spectra observed after 5 min incubation was again changed after 60 min. This changed CD spectra of proteins suggests the conformational changes in the protein structure on interaction with GO. Similarly, the interaction of spontaneously adsorbed ovalbumin (OVA) on GO nanosheet demonstrated reduction in negative band at 222 nm corresponding to α helix of OVA with increase in GO concentration, indicating denaturation of OVA upon adsorption on GO nanosheet. In another study, Lee et al.^200^ inferred that secondary structure of insulin got altered on interaction with graphene by reducing α-helix content because of strong π–π interaction, whereas due to the moderate hydrogen and electrostatic bonding the conformation of the insulin retained on GO surface irrespective of the adsorption time (Figure 6A-iii). Over time, the conformation of insulin kept denaturing on adsorption onto graphene, while GO had no deteriorating impact on insulin’s secondary structure.

### 4.4 Isothermal Titration Calorimetry (ITC)

ITC is a useful technique to study interactions between two molecules by determining the thermodynamic parameters of the interactions in solution. ITC exploits the fact that when different molecules interact, the reaction is either endothermic or exothermic. It directly measures the heat involved in the interaction, using a sensitive calorimeter. The progressive titration of the protein (ligand) into the nanosheet solution (sample cell) allows the sensitive calorimeter to take a measurement, thereby providing information regarding thermodynamic characterization like stoichiometry, binding affinity, dissociation constant, association constant, and binding enthalpy, that too without the need of reporter labels, e.g., chromophores and fluorophores.^201^ The knowledge of thermodynamic parameters helps to estimate the interaction between protein and nanosheet. The information on enthalpy (Δ*H*) and entropy (Δ*S*) can reveal the interaction involved. Positive enthalpy (Δ*H* > 0) and positive entropy (Δ*S* > 0) reflect the presence of hydrophobic interactions, whereas negative enthalpy (Δ*H* < 0) and negative entropy (Δ*S* < 0) are indicative of vdW forces or hydrogen bonding. Also, negative enthalpy (Δ*H* < 0) and positive entropy (Δ*S* > 0) reveal the presence of electrostatic force. ITC was employed by Shahid et al.^153^ to quantify the interaction of ubiquitin with GO of varying oxidation degrees. Figure 6B-ii,iii shows the results of ITC analysis of ubiquitin with GO of varying oxidation degrees. The proteins upon interacting with the surface of GO sheets underwent a fast dynamic exchange with the free proteins in solution. The results showed that the *K*_d_ values were in the micromolar range, which indicated low binding of ubiquitin onto GO nanosurface on account of weak electrostatic forces between them.

### 4.5 Molecular Dynamic Simulation

MD simulation provides theoretical insights about protein–nanosheet interactions by analyzing molecular (microscopic) level changes and understanding its impact at macromolecular level. It provides detail about dynamic changes as a function of time, and the real time analysis helps in monitoring each detail about how the protein is getting adsorbed on the nanosheet. Being a computational model, it helps in analyzing the impact of different parameters in a controlled way that might otherwise not be possible with the actual experimental setup. The results obtained from MD simulations are also validated using experimental setup: Generally, an experiment is first simulated using different nanosheets for different proteins at various physiochemical conditions, then the most favorable complex in terms of energy and stability is selected. The study can be controlled by altering/removing/adding different functional groups present on protein and nanosheet, thus enabling the understanding of the role of each of these functional groups in the interaction between protein and nanosheet. Utilizing MD simulation, we can know the protein folding and unfolding, conformational change in both systems, energy changes, type of interaction, stability of the complex, impact of internal and external factors, etc., and all these could be validated by experiment or taken into consideration while designing an experiment involving both proteins and nanosheets.^202−205^

**Figure 7:**
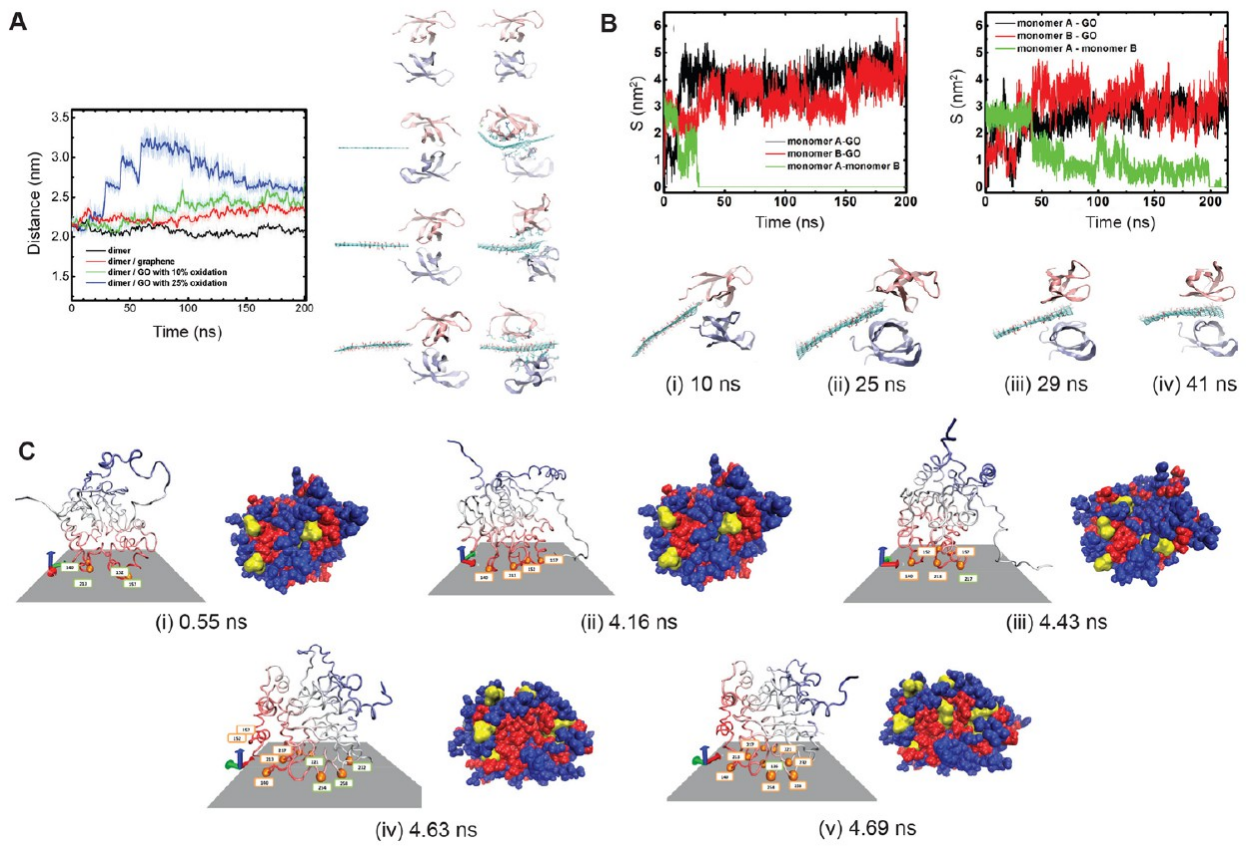
MD simulation analysis of 2D nanomaterial–protein interaction. (A) (a) Analysis of time-dependent distances between two monomeric unit of proteins in presence and absence of GO nanosheets. (b–e) Initial and the final conformation of each protein monomers in presence and absence of GO nanosheets. (B) (a) and (b) Study of time-dependent contact areas of protein–protein and protein–nanosheet (GO). (c–f) Illustration of GO inserting into the dimer. Reproduced with permission from ref (163). Copyright 2016 American Institute of Physics. (C) In each panel, the left figure shows the interaction of HLD-A141C on MoS_2_ along the simulation trajectory. Orange dots: Amino acid residues adsorbed on the surface. The right figure provides hydrophobicity surface mapping of HLD-A141C on MoS_2_ Red: hydrophobic amino acids. Blue: hydrophilic amino acids. Yellow: hydrophobic residues that are corresponding to the orange dots shown in the trajectory portraits on the left. Reproduced with permission from ref (77). Copyright 2019 American Chemical Society.

Feng et al.^163^ investigated the interaction between GO nanosheets and HIV-1 IN homodimer structure using all atom MD simulations (Figure 7A,B). The simulation was mainly performed to investigate the impact of graphene based nanosheets on protein (HIV-1) structure upon interactions. The experiments were done on a protein-alone system, a protein with graphene system, a protein with 10% oxidized GO system, and a protein with 25% oxidized GO system. Parameters analyzed with their results were as follows: (i) Dissociation of protein dimer: Nanosheets were able to dissociate the monomeric units of HIV-1 IN homodimer by integrating itself through hydrophobic interactions. Steric hindrance and attractive force between oxidized group of GO nanosheets and protein residues provided an unfavorable environment for dissociation. (ii) Degree of oxidation: The distances between monomeric units increased on moving from pristine graphene to 10% GO and further to 25% GO system. These reasons were also responsible for slower insertion of GO sheets (increasing time with higher oxidation) than pristine graphene. Another reason responsible might be higher thickness of GO nanosheets. (iii) Time-dependent interaction energy: vdW interaction between each monomeric unit and nanosheet was found to be higher than the interaction between both the monomeric units themselves. The observation determined the vital role of vdW interaction in insertion of nanosheets. (iv) Impact on monomeric structure: Time-dependent root mean-square deviations (RMSDs) were calculated. The results showed that nanosheets had no impact on the monomeric structures, and its stability was maintained. The results obtained showed that pristine graphene could have more adverse effect on protein dimer than GO, because of strong hydrophobicity and comparatively less thickness and more flexibility. In another model, Luan et al.^164^ studied the cytotoxicity caused by interference of graphene disrupting the protein–protein interaction (PPI). They measured the time-dependent contact areas, time-dependent interaction energy, and time-dependent root mean-square deviations (RMSDs) of PPI both in presence and absence of graphene. The MD results of their study showed that graphene inserted into the protein–protein dimer disrupts the PPI. Insertion of graphene can destabilize the hydrophobic interaction of the PPI and the complex of the proteins can be broken. The flatness and the strong hydrophobicity of graphene are responsible for the reduced vdW potential energy between graphene and the PPI. However, graphene is the least responsible for any energetic change and did not hamper the hydrophilic interactions. Conclusively, if graphene enters a cell, then it may disrupt the hydrophobic interaction of the PPI, which may directly or indirectly affect the functionality or mortality of the cell.

MD simulation was also used to study the interaction of GO and RGO with blood plasma proteins (BSA, BFG, Ig, and Tf) by Chong et al.^142^ The results showed that the binding affinity of the concerned proteins on both GO and RGO ranked in the order (BFG > Ig > Tf > BSA). The results revealed that the binding was mainly due to enhanced surface area of the nanosheets because of flat surface. Protein adsorption occurred through strong π–π stacking interactions between the aromatic residues of the protein and GO/RGO nanosheet surface. Xiao et al.^77^ explored the interaction of MoS_2_ and WS_2_ with β-Gal-D308C, β-Glu, and HLD-A141C as model proteins. The results showed that HLD-A141C gets adsorbed on the TMDC nanosheet without any significant conformational change while the other two proteins did not show substantial adsorption. Figure 7C shows MD simulation of interaction between HLD-A141C and MoS_2_. The results obtained were similar for both the TMDC nanosheets, i.e., MoS_2_ and WS_2_ for all the three model proteins. As all three proteins had a unique surface-exposed cysteine, difference in adsorption pattern indicates that the force responsible for interaction is majorly hydrophobic and no disulfide bond is formed between the thiol group of cysteine residue and the sulfur of TMDC. In another study, Xiao et al.^167^ studied the interaction of alpha helical peptide, a peptide of cecropin, and melittin on MoS_2_ through MD simulation. Their results showed that charged group on N terminus are required for the “standing up” position of the peptide on MoS_2_ surface. Charged group on the C terminus helps in leading the peptide to lay down on MoS_2_ which was also validated by optical microscopy and SFG spectroscopy. Yao et al.^160^ investigated the interaction of trypsin with GO through MD simulations which revealed that trypsin was adsorbed through its cationic and hydrophilic amino acids onto GO surface; the active site of trypsin gets covered by GO which remain stabilized at high temperature. Positively charged residues of trypsin forms strong electrostatic bonding, whereas the neutral residues show strong vdW interactions with GO. The active site interacts with nanosheets and thus prevents conformational denaturation of protein at higher temperatures. Thus, the analysis of protein and nanosheet interaction can be theoretically estimated using MD simulation at varied conditions.

Recently, Bisht et al.^206^ have extensively studied the interaction of MoS_2_ nanosheets with SARS-CoV-2 Spike protein, its human receptor ACE2, and the receptor–ligand complex Spike–ACE2. It was observed that MoS_2_ nanosheet binds strongly to Spike, ACE2, and the complex structure. Interestingly, along with other binding sites, MoS_2_ nanosheets bind stably with the receptor binding domain (RBD) and especially the receptor binding motif (RBM) region of the Spike protein, as well as to the Spike-interacting region (hotspot amino acid region) of the ACE2 receptor. Moreover, these nanosheets also exhibited binding to the glycosylated spike protein of SARS CoV-2 and its variants: kappa and delta. The flat surface of MoS_2_ nanosheet with sulfur atoms interacted directly with amino acids via hydrogen bonding and van der Waals interaction, whereas the Mo atoms at the edge of the sheets interacted with the amino acids to a lesser extent through electrostatic interactions. It was further observed that upon interaction, the receptor binding motif (RBM) region of RBD underwent considerable secondary structure changes, which might influence the receptor–ligand interaction. To further investigate the impact of MoS_2_ binding on the Spike–ACE2 protein complex, two systems were considered which involved the stable structure of the Spike–ACE2 complex and another system which involved a nanosheet–RBD complex bound to ACE2. The binding of MoS_2_ exhibited maximum solvent accessibility and less stability of the protein–protein complex as compared to the crystal structure. The molecular mechanics with generalized Born and surface area solvation (MM/GBSA) analysis reported that the binding of MoS_2_ also influenced the interaction energies between the key interacting amino acids of the Spike–ACE2 complex, especially when the MoS_2_ nanosheet binds to the motif region of the complex, which results in destabilization of the Spike–ACE2 complex. Finally, the dewetting analysis revealed the quick adsorption of all the protein systems on the MoS_2_ nanosheet, especially the nanosheet–RBD complex with ACE2, supporting the observation of strong and stable adherence of the nanosheet on RBD, thereby leading to weak interactions with ACE2 receptor. Thus, MD simulation analysis helped to unravel the nature of interactions between 2D MoS_2_ and SARS-CoV-2 viral proteins, along with its receptor ACE2, and also opened up a possibility to further explore the potential of this material as an antiviral nanoagent.

### 4.6 Some other Analytical Techniques

Besides the various techniques discussed above, other analytical techniques like Raman spectroscopy, XRD, etc., are also used to study the interaction of proteins with nanosheets. Some of these techniques have been mentioned below briefly.

#### 4.6.1 Raman Spectroscopy

Raman Spectroscopy is an inelastic scattering based analytical technique which enables understanding about chemical structure, crystallinity, and molecular interactions. It provides structural imprints to identify molecules and relies upon the interaction of light with the chemical bonds within a material.^207^ Kukkar et al.^156^ designed a MoS_2_ based biosensor for detection of BSA, where they employed Raman Spectra to analyze the presence of BSA on MoS_2_ surface. The presence of additional peaks, besides E_2g_^1^ and A_g_^1^, at 1600 and 2800 cm^–1^ (originating due to tyrosine ring stretching and aliphatic and amide vibrations respectively) confirmed the presence of BSA. Zhezhu et al.^136^ used Raman spectra to understand BSA adsorption on GO surface. They found that intensity of Raman spectra of free BSA decreased on binding with GO nanosheet, thus confirming interaction between BSA and GO. Lu et al.^146^ decorated RGO with β-lactoglobulin and studied GO, RGO, and BLG-RGO Raman spectra to distinguish the ordered and disordered structure of graphene. In order to determine the defects and the sp^2^ domain size, the intensity ratio of D and G band (*I*_D_/*I*_G_) was quantified and was found to be 1.37 for RGO while that for BLG-RGO it was 1.29. The decreased value of (*I*_D_/*I*_G_) ratio of BLG-RGO than RGO is indicative of evolution of sp^3^ to sp^2^ structure of RGO. Thus, Raman spectroscopy can also be a useful tool for chemical analysis and to identify molecular interactions between a nanosheet and a protein.

#### 4.6.2 X-ray Diffraction (XRD)

XRD is an analytical technique that is used to reveal structural information, mainly crystal structure and chemical composition. It is based on the fact that every crystal has a distinct 3D diffraction pattern, and it takes into account the constructive interference of monochromatic X-rays of the incident beam and a crystalline sample. Thus, this technique can be used to characterize the interaction between protein and nanosheet. Shicun et al.^208^ used XRD patterns to analyze the interaction between soya protein isolate (SPI) and poly dopamine functionalized GO (PDG). They observed that the pristine SPI showed two peaks at around 2θ = 9.2° and 20.1° representing the α helix and β sheet conformations of the protein. On adsorption of SPI to PDG, the α helix peak shifted to lower angle indicating denaturation in the α helix structure of the protein, thus exhibiting reduced degree of crystallinity and showing strong cross-linking between SPI and PDG.

## 5 2D Nanomaterial–Protein Interactions: Case Studies

### 5.1 Proteins Interacting with Graphene-Based 2D Nanomaterials

#### 5.1.1 Chymotrypsin

**Figure 8:**
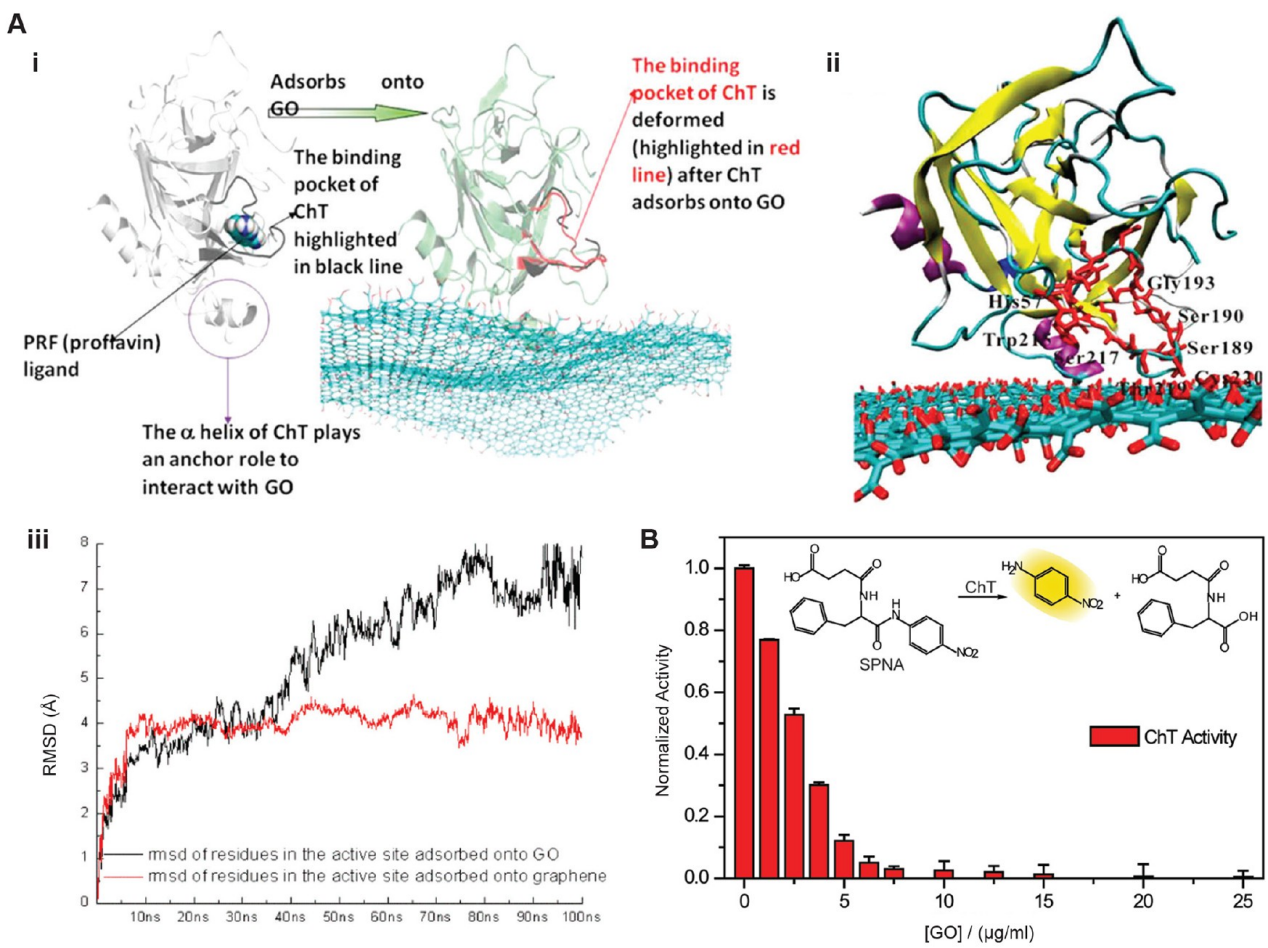
Activity of chymotrypsin with change in concentration of GO. (A) (i) Adsorption of ChT onto GO deforms its active site. (ii) Snapshot of the active site of ChT. (iii) Average rmsd of the active site of ChT during its interaction with GO and graphene. Reproduced with permission from ref (165). Copyright 2014 American Chemical Society. (B) Analysis of ChT activity with increase in GO concentration with SPNA as a substrate. Reproduced with permission from ref (159). Copyright 2011 American Chemical Society.

Sun et al.^165^ explored the interaction of the enzyme chymotrypsin with carbon based nanosheets, i.e., graphene and GO using MD simulation. Chymotrypsin is a serine protease which is synthesized in the pancreas in an inactive form, chymotrypsinogen. This inactive form gets converted into the active enzyme chymotrypsin in the small intestine by another enzyme trypsin. The active chymotrypsin acts as a digestive enzyme and cleaves the C-terminal amino acid residues of peptides. Sun et al. found that the position and conformation of the S1 pocket which determines the efficiency and specificity of the enzyme is important for enzymatic activity. MD simulations revealed that ChT gets adsorbed on graphene and GO with different curvature and contact areas. The most important role is played by the hydrophobic residues of the protein, viz., isoleucine, valine, proline, and alanine, during interaction with graphene. The aromatic amino residues are responsible for the π–π stacking and CH−π interactions with graphene surface. During such interactions, the S1 pocket of the enzymes, however, remains far away from the graphene surface, allowing only weaker interaction and thus the enzymatic activity remain intact. In contrary, while interacting with GO, the main region of enzyme interacting is the α helix domain which forms the anchoring point after being brought closer to the GO surface by the cationic residues. The hydrophilic amino acid residues especially lysine and arginine interact with the oxidized region of GO. The epoxide or carbonyl groups present on GO surface forms strong hydrogen bonds with these hydrophilic residues. Subsequently, the active site of ChT also gets adsorbed on the GO surface leading to large deformation of the binding site of the substrate. Figure 8A-i shows the active site of the enzyme interacts with GO and prevents its interaction with the substrate. The main amino acids responsible for its catalytic activity (histidine, aspartic acid, and serine) are directly adsorbed onto the GO surface (Figure 8A-ii). Further, a ring of positively charged amino acids are formed around the active site which interacts with anionic groups of GO leading to deformation of the active site and inhibiting its enzymatic activity. The average RMSD of the residues in the active sites of the enzyme (Figure 8A-iii) clearly depicted that enzyme activity was significantly affected by immobilization on GO than on graphene. These results corroborated the experimental results (Figure 8B) which showed that the ChT enzymatic activity gets inhibited in the presence of GO in a concentration-dependent manner.

#### 5.1.2 HRP and Lysozymes

Zhang et al.^137^ investigated the use of GO nanosheets as substrates for immobilization of enzymes such as horse radish peroxidase and lysozymes. They observed that the presence of numerous oxygen containing functional groups on the surface of GO nanosheets were sufficient to immobilize the enzymes on the nanosheet surfaces without any surface modification or coupling agents. While studying the nature of such interactions which made the enzyme immobilization possible, they found out the role of electrostatic interactions in formation of noncovalent linkages between the positively charged amino acid residues of the enzymes and the negatively charged carboxyl groups, among the many oxygen containing surface functionalities present on the surface of GO. It was further noted that the pH of the medium played a very important role in the interaction between the enzymes and the nanosheets, since the charge of the amino acid residues changes with change in pH. HRP enzyme, having its isoelectric point at pH 7.2, exhibits a net positive charge below this pH and a net negative charge above it. Similarly, lysozyme exhibits a net positive charge below pH 10.3 and net negative charge above it. Therefore, considering the fact that GO is negatively charged in aqueous solution in the entire pH range 4–11, it will form strong electrostatic interactions with HRP in an acidic pH range, while it will repel HRP at basic pH. For lysozyme too, strong interaction was observed with GO at pH lower than 10.3. Both HRP and lysozyme showed high loading onto the surface of GO at pH 7.0. The high enzyme loadings revealed the exceptional potential of GO to act as a solid substrate for enzyme immobilization. Apart from electrostatic interactions, the involvement of hydrogen bonding between the negatively charged oxygen containing surface functionalities of GO and basic residues of the enzyme was also observed. However, it was also noted that the electrostatic immobilization of the enzymes onto the GO surface resulted in a decrease in the enzymatic activity of the immobilized enzymes in comparison to free enzyme. The authors believed that such a decrease in biological activity of the enzymes was due to conformational changes induced in the enzyme structure by its binding to GO. On the other hand, Zhang et al.^209^ demonstrated that in addition to electrostatic interactions and hydrogen bonding, hydrophobic interactions also contribute significantly toward immobilization of enzymes on GO. Experiments with chemically reduced GO (CRGO), having a comparatively lesser amount of oxygen containing functional groups than GO and hence a lesser possibility of electrostatic interactions, showed remarkable higher loading of enzymes such as HRP and oxalate oxidase onto its surface. This was mainly due to the hydrophobic interaction between the enzymes and the CRGO. Also, the stability and enzymatic activity of CRGO–enzyme conjugates was higher than GO–enzyme.

#### 5.1.3 Heparin

Graphene is hydrophobic in nature. Strong dispersive forces exist in between the graphene plates which make it insoluble in an aqueous environment. Studies conducted by Lee et al.^65^ showed that highly stable aqueous dispersion of chemically reduced graphene can be produced through noncovalent interactions with heparin. Physiological stabilization of graphene can be done through noncovalent methods where various π-rich water-soluble polyelectrolytes successfully produce stable graphene dispersion using π–π interactions with graphene nanosheets and polyelectrolytes. Heparin is rich in sulfonate, a group which gives it a very high negative surface charge density, and also it has a relatively hydrophobic cellulose backbone. Thus, the interaction between heparin and graphene nanosheets mostly take place through hydrophobic interactions between graphene nanosheets and the heparin backbone, and the presence of negative charge on the graphene–heparin conjugate stabilizes them in aqueous medium through charge repulsion. The formed graphene–heparin conjugate was also found to retain its biological activity and demonstrated ∼30-fold increase in anticoagulant activity in comparison to GO, thereby validating the fact that the heparin molecules after attaching to the surface of graphene nanosheets retained their biological activity.

#### 5.1.4 Hemoglobin

Interactions of hemoglobin (Hb), a blood protein, with carbon nanosheets can shed light on how such nanosheets are expected to behave under *in vivo* conditions and also provide more information related to biocompatibility, bioavailability and toxicity of such nanosheets. Studies carried out by Wang et al. on the interactions of GO (GO) with BHb have revealed some key information on how the structural and functional properties of Hb gets altered through binding interactions with GO.^155^ It was observed that binding of Hb on the surface of GO nanosheets had serious consequences on the structural integrity of the bounded Hb molecule. CD spectroscopy revealed that the binding interactions caused a decrease in α helix content of Hb along with an increase in random coil and beta sheets. This indicates that the secondary structure of the Hb molecule suffered major damages. The spectra also showed an unfolding of the peptide strand taking place as a result of interaction with GO, which was further supported by UV–vis spectroscopic studies. The UV–vis spectroscopic results further revealed that in addition to reduction in the α helix content of the Hb molecule and loosening of the skeleton of the molecule, interactions of GO with Hb also caused exposure of the heme group and aromatic amino acid residues of Hb to the aqueous environment, thereby clearly ascertaining the event of protein unfolding taking place. Temperature-dependent studies demonstrated the effect of GO on the thermal stability of Hb which showed that in the presence of GO, the Hb molecule was more prone toward thermal denaturation. GO was also found to inhibit nonenzymatic glycosylation of Hb. Computational modeling studies showed that two binding modes, i.e., insert binding mode and surface binding mode existed for the interaction of BHb with GO. In both the binding modes, hydrophobic interaction was the main driving force. However, other forces like hydrogen bonding, electrostatic bonding, and π–π stacking also contributed toward the adsorption of Hb onto GO surface.

#### 5.1.5 Plasma Proteins

Interaction of graphene-based nanosheets with plasma proteins is of great importance for various biological and biomedical applications, like bioimaging, drug delivery, diagnosis, etc. Kenry et al.^148^ observed the interaction of abundant plasma proteins like albumin, globulin, and Fg with GO of different lateral size. The interaction of some abundant plasma proteins in discussed below, where we will see how different proteins have different adsorption behavior with nanosheets.

##### 5.1.5.1 Albumin

Ding et al.^210^ conducted conformational studies, on the various types of interactions of human serum albumin (HSA) using differently functionalized GO such as GO, GO–COOH, GO–polyethylenimine (PEI) and GO–CS. They found that nanosheets readily interact with HSA and the binding affinity was found to be highest for GO and lowest for GO–PEI. The interacting forces between HSA and the nanosheets may include hydrophobic interactions, electrostatic interactions, hydrogen bonding and π–π stacking interactions. Hydrogen bonds are formed when positively charged amino acids residues like arginine and lysine interact with the negatively charged surface functionalities of GO such as the epoxy groups on the GO surface. GO–COOH interacts with HSA using hydrogen bonds as the dominant driving force between the carboxyl group and the positively charged amino acids since the carboxyl groups block the epoxy groups. A positive change in entropy was detected in ITC during the study of interactions with GO–PEI and GO–CS which indicates the role of hydrophobic interactions in binding with HSA. The results also indicate that the binding mechanism starts off with initial hydrophobic interactions which exposes the inner structure of HSA to the GO nanosheets. HSA shows the strongest hydrophobicity when it is at its isoelectric point. Zeta potential studies confirm the presence of strong electrostatic forces when HSA and GO interact, the interaction being strongest at a pH between 4.0–9.0. Efficient fluorescence quenching was also observed at a lower pH. Increase in quenching indicates more interactions between HSA and GO. At a lower pH, HSA is positively charged and hence a stronger interaction with the negatively charged GO surface is exhibited which is responsible for the quenching process.

In another study conducted by Kenry et al.,^148^ the effect of size of GO nanosheets on its interaction with plasma proteins like albumin was extensively studied. They found that albumin exhibited highly consistent adsorption behavior at lower concentrations. Increase of adsorption of the albumin was observed with increase in size of the nanosheets. Thus, GO having the largest lateral size showed lowest absorption of free albumin and the GO nanosheet having the smallest size showed the maximum absorbance for free albumin. Furthermore, fluorescence studies revealed that albumin produced the largest red-shift as compared to other blood proteins, irrespective of the size of the nanosheets. In the presence of GO nanosheets with a smaller lateral size distribution, albumin showed an exponential decrease of its fluorescence intensity ratio. This confirmed the presence of both dynamic and static quenching. Besides quenching, the interaction of albumin with GO also resulted in conformational change of the albumin structure.

##### 5.1.5.2 Fibrinogen (Fg)

On studying the adsorption of fibrinogen (Fg) with different sizes of GO, the outcomes observed were reverse in comparison to that obtained for albumin.^148^ At lower concentrations of Fg, adsorption process is independent of the lateral size of the nanosheets. At a higher concentration, all the nanosheets exhibit maximum adsorption of Fg. But when exposed to the nanosheet with a larger lateral size distribution, there was lesser adsorption. This lower adsorption can be due to saturation of the loading capacity of the nanosheet surface. The lateral size-dependent adsorption of Fg started at a specific concentration of 10 mg/mL. After reaching this specific concentration, the adsorption of Fg increases with decrease in lateral size. The red-shift of Fg molecule in fluorescence emission studies was unique because it showed an increase in the red-shift with increase in lateral size. The characteristic red-shift trends in Fg may indicate an increase in the polarity and hydrophobicity of the local environment of the emission active elements. In the presence of GO nanosheets with large surface area, Fg showed a linear trend in its fluorescence intensity ratio, indicating dynamic quenching. However, the comparison of fluorescence quenching studies of Fg under the influence of GO sheets with different lateral size distribution showed an exponential trend with the decrease in the lateral size of the nanosheets, thereby also indicating the presence of static quenching. Thus, the quenching observed is a result of both static and dynamic quenching. Similar to albumin, conformational change was also observed in case of Fg on interaction with GO.

##### 5.1.5.3 Globulin

Unlike albumin and Fg, globulin did not show any significant trend of adsorption with respect to size of GO nanosheet.^148^ The GO nanosheets of different lateral size distributions showed maximum globulin adsorption at low concentration of the protein molecule. However, at higher globulin concentrations, some amount of free globulin absorbance was detected. Highest adsorption of the molecule occurred in nanosheets with the highest lateral size distribution and vice versa. The red-shift in fluorescence spectra of globulin was more or less similar to that of albumin, regardless of lateral size distribution of nanosheets. The fluorescence intensity ratio of globulin also had an exponential trend similar to albumin. Thus, globulin also exhibited both static and dynamic quenching.

### 5.2 Proteins Interacting with TMDCs

#### 5.2.1 HP35

**Figure 9:**
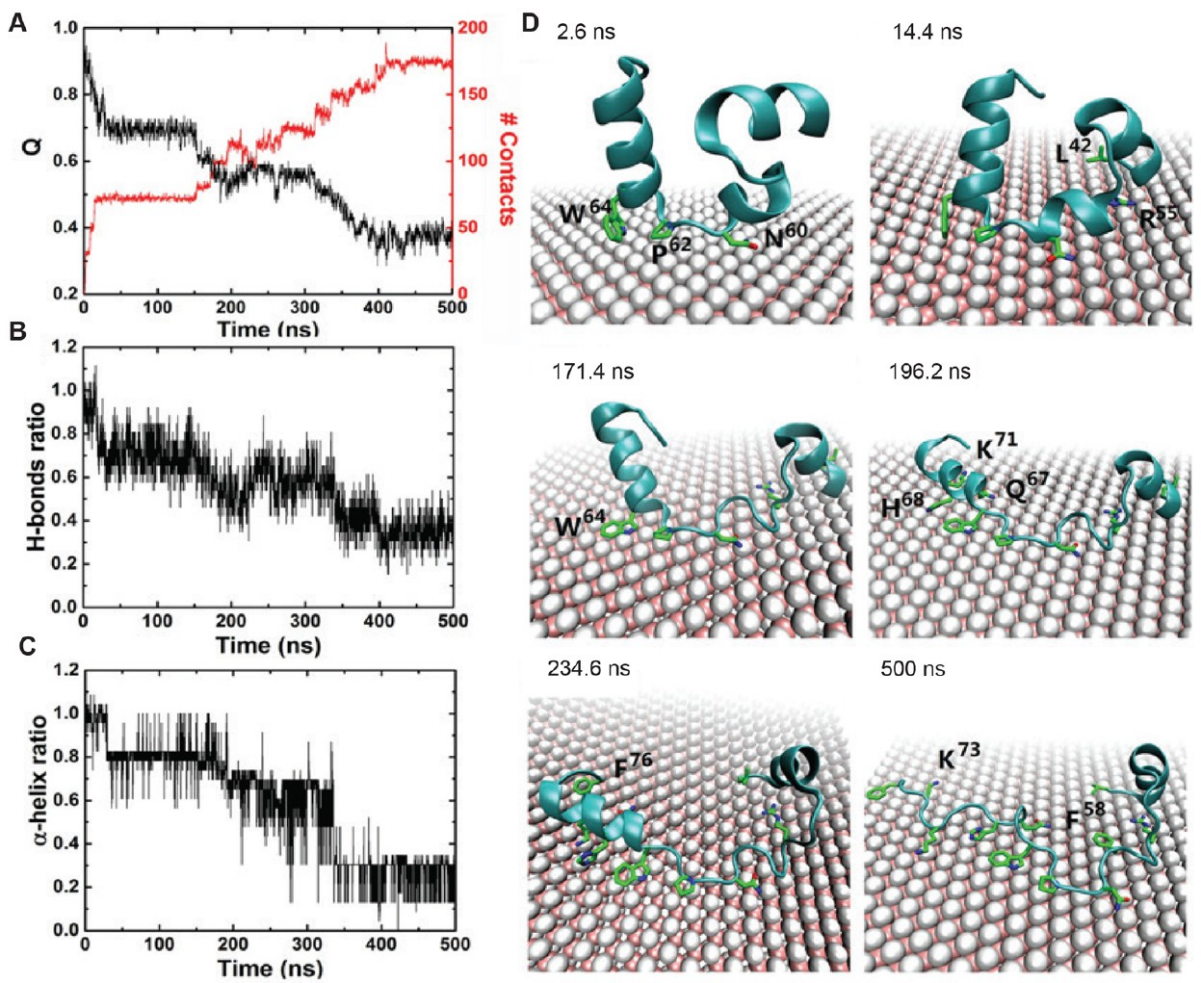
Structural dynamics of HP35 on MoS_2_ surface. Reproduced with permission from ref (162). Copyright 2016 Springer Nature. (A) Time profile of native contact Q of HP35 (black) and heavy atom contact number between HP35 side chains and MoS_2_ (red). (B, C) Hydrogen bond and α helix ratios of HP35 adsorbed onto MoS_2_, as a function of time. (D) Representation of simulation trajectory of important intermediate structures of HP35.

To gain a deeper understanding about the biocompatibility of TMDCs such as MoS_2_, Gu et al.^162^ studied the interactions between the villin headpiece (HP35) protein and MoS_2_ nanosheet using all-atom molecular dynamics simulations. The simulations showed that interactions between HP35 protein and MoS_2_ nanosheets severely affected the conformation of HP35 native folds, leading to its denaturation. Most of the alpha helical structure of HP35 faced a significant damage due to strong dispersive interactions with MoS_2_ surface. HP35 loses its native contact with the nanomaterial rather quickly due to the degradation of its initial contact areas, which are the residues from the second and third alpha helices in the structure of HP35 (Figure 9). The initial contact was made by the amino acid residues present in the second α helix, which turned out to have high proportion of hydrophobic aliphatic and aromatic residues, indicating that hydrophobic interaction was the main driving force in the initial adsorption/anchorage process. The second helix contains the highest proportion of hydrophobic residues which is why it is the first to be adsorbed. The interaction between the initial amino acid residues induced contacts of neighboring residues such as Leu and Arg to settle down on the nanosheet surface, thereby anchoring the helix onto the surface of the nanosheets. This in turn led to some N-terminal residues to come in contact with the nanosheets as well. The residue Trp-64 required further stabilizing; thus, a large conformational change occurred in the indole side chain which induced adsorption toward the C-terminal region. This triggered the third α helix to settle down on the nanosheet’s surface. The system reached its energy minimum when the Phe-76 in the C-terminal settled on the nanosheet. After that, a few more changes continued taking place until the minimum was reached. Hydrophobic interactions during initial adsorption may be the driving force for protein–nanomaterial binding, but protein also favors interaction with nanomaterials through electrostatic and dispersive vdW energies. vdW’s forces were found to be dominant in the adsorption kinetics of HP35. It was observed that most of the residues such as Arg-55, Phe-58, Trp-64, Lys-70, Lys-71, and Phe-76, which made up the second and third helix of the protein and contributed toward the adsorption of the protein on the nanosheet surface had vdW’s energies less than −20 kcal/mol, thereby showing the key contribution of vdW’s forces in mediating this interaction. In addition to this, the presence of basic residues such as Arg-55, Lys-70, and Lys-71 in this same interacting group also sheds light on the possible involvement of polar interactions too during the binding process. Arg-55 was found to play a crucial role in tethering the protein to the surface of the nanosheets. It was observed that this residue was the one holding on to the second helix while it was being denatured. Thus, the interaction between the model HP35 protein and MoS_2_ nanosheets provides a peek into the molecular origin of potential nanotoxicity of this material and further establishes the need for developing better surface functionalizing chemistries to modulate the behavior of these nanomaterials with biomacromolecules such as proteins, lipids and DNA.

#### 5.2.2 Plasma Proteins

Interaction of plasma proteins with TMDCs nanosheets is important for understanding the mechanism of action and immune response generated by nanosheet *in vivo*. Baimanov et al.^145^ investigated the interaction of MoS_2_ nanosheets (NSs) with blood plasma proteins and studied their cellular uptake. The dissociation constants obtained from the four proteins adsorbed on MoS_2_ nanosheet represented the different binding affinities of these proteins. ITC spectra and ITC thermogram of HSA, IgG, and Tf showed much higher binding affinities toward MoS_2_ as compared to Fg. The relation between thermodynamic parameters (entropy and enthalpy) and the interaction type were utilized to study the type of interaction of MoS_2_ with HSA, IgG, Fg, and Tf. The negative values of Δ*H* and Δ*S* revealed that the predominant interactions responsible for interaction of MoS_2_ with HSA, IgG, Fg, and Tf were vdW’s force and hydrogen binding. TEM micrographs were utilized to reveal the presence of plasma proteins on the MoS_2_ nanosheet surface and observe the cellular uptake of the protein adsorbed nanosheets in a periodic manner. The internalization of NSs–protein complexes in lysosome of the cells enhanced with time, and different NSs–protein complexes showed different uptake. Both bare NS and NS-corona were phagocytosed via endocytosis into the macrophages and accumulated as irregular aggregates in the lysosome dispersed in the entire cytoplasm of the cell. Understanding the fate of NS–protein complex is important for various applications like drug delivery, photothermal treatment, etc. Interaction of various blood plasma proteins with TMDCs is discussed in below.

##### 5.2.2.1 Albumin

Albumin is one of the most important plasma proteins; its interaction with MoS_2_ nanosheets is because of transfer of electrons. Baimanov et al.^145^ in their study found that MoS_2_ nanosheets can adsorb large amount of plasma proteins, with maximum adsorption of HSA. HSA showed higher binding affinity and low dissociation for MoS_2_ nanosheets. The interaction of HSA with nanosheet resulted in decrease in the α helix content and an increment of the random coil content in comparison to free HSA, which suggests that NS induced denaturation and unfolding in HSA. Further, it is found that HSA on adsorption on the MoS_2_ surface acts like electron donors. This was validated from the fluorescence quenching studies, conducted by Zhang et al.^134^ that showed when the concentration of MoS_2_ nanosheets in the solution was high, the intrinsic fluorescence of HSA was quenched by 60%.The intrinsic fluorescence of HSA is due to presence of aromatic amino acid residues like tryptophan and tyrosine. The transfer of electrons from aromatic tryptophan amino acid of HSA neutralizes the negative charge on the surface of MoS_2_ nanosheets, and this electron transfer was the main reason for the quenching of fluorescence observed. This kind of an arrangement can be very useful when detecting the presence or absence of foreign molecules or ions. The fluorescence intensity or lifetimes are affected by the addition of the probe molecules and thus the BSA-TMDC composites can act as efficient fluorescent turn ON/OFF sensors.

##### 5.2.2.2 Transferrin (Tf)

Another plasma protein, transferrin (Tf),^145^ showed good binding affinity toward MoS_2_ nanosheets and was adsorbed in moderately good amount. The interaction of Tf with MoS_2_ nanosheets caused some conformational change of Tf, with decreased α helix content and enhanced random coils. It was also found that Tf coated NS showed less immune response and successfully camouflaged NS, which makes it a promising candidate for in vivo biological applications like drug delivery.

##### 5.2.2.3 Fibrinogen

Fg was the least adsorbed protein on the MoS_2_ nanosheets in comparison to albumin, Tf, and IgG.^145^ It was observed that it has the highest dissociation rate and the weakest affinity toward the nanosheet. Though, protein quantification assays showed that it has the most mass adsorbed onto the surface of the nanosheets, but the binding affinity of the protein molecules was significantly low. A significant alteration in Fg structure was observed on adsorption with decrease in α helix content from along with an increase in the β sheet content. Hydrogen and vdW noncovalent bonding is thought to be responsible for Fg adsorption on MoS_2_ nanosheets.

##### 5.2.2.4 IgG

Immunoglobulin (IgG) adsorbs on to the surface of the MoS_2_ nanosheets by virtue of vdW forces and hydrogen bonding.^145^ IgG showed similar binding affinity and dissociation constant as that of albumin and Tf. As it possesses largest amount of beta sheets compared to the other proteins investigated, it did not undergo any significant change of conformation, even after adsorption on the nanosheets. This example suggests that the shape of the protein and the β sheet content of the protein may play an important role in adsorption of the protein on the nanosheets.

### 5.3 Proteins Interacting with Xenes

#### 5.3.1 HP35

**Figure 10:**
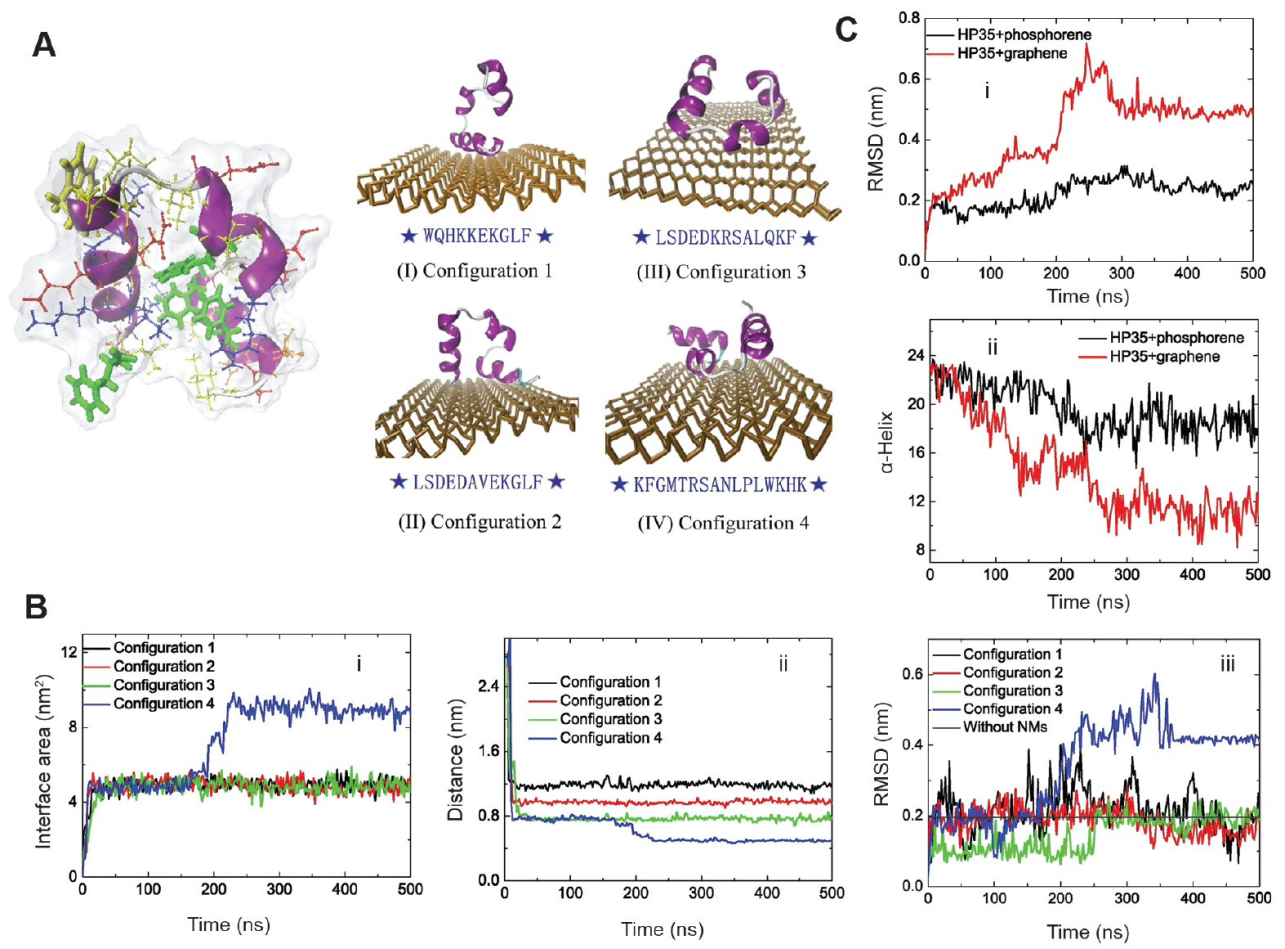
MD simulation study of the interactions between HP35 and Phosphorene nanosheets. (A) Native structure of HP35. Simulated representation of contact residues of HP35 along with their contact configurations adsorbed onto phosphorene. (B) For the four configurations, the interface area (i), mean distance between HP35 and phosphorene (ii), and the RMSD of HP35 adsorbed onto phosphorene (iii), was studied as a function of time. (C) Averaged RMSD (i) and number of residues in the a-helix structure (ii) of the four contact configurations of HP35 adsorbed onto the graphene and phosphorene as a function of time. Reproduced with permission from ref (127). Copyright 2015 Elsevier.

Zhang et al.^127^ investigated the interaction between villin headpiece of HP35 protein with phosphorene and graphene using MD simulations. HP35 has five aromatic sites, i.e., F06, F10, F17, W23, and F35; out of which W23 and F35 were involved in interaction with phosphorene and F10, W23, and F35 were actively involved in interaction with graphene. It was revealed that phosphorene caused less disruption to the HP35 structure than graphene (Figure 10). In order to check the damages caused to the secondary structures of HP35, the number of residues present in the alpha helical structures of HP35 adsorbed onto phosphorene were analyzed. The authors report that only a part of the third α helix got converted to 3_10_-helix and that the remaining part lost the α helix content, while the other two helices retained their α helix structure, indicating that severe damage was not done. Investigations to understand the reason for the less disruptive nature of phosphorene revealed that the surface morphology of the nanosheet and type of interactive forces between the nanosheet and the protein played key roles in determining the outcome of this interaction. It was observed that the presence of a puckered “valley” morphology of phosphorene resulted in weaker interactions with HP35, which significantly reduced the disruption of the native HP35 conformation. This finding further shed light on the strong protein disruptive nature of graphene, which possesses a flat surface and thus interacts more strongly with protein/peptide residues. The results showed that π–π stacking and hydrophobic interactions were responsible for absorption of HP35 on nanosheets surface. Also, the dispersive (vdW) forces between phosphorene and HP35 were found to be very weak. This is again a direct consequence of modification of nanosheet surface morphology through “puckering” which attenuated the dispersive interactions between the two. Overall, the nondisruptive nature of phosphorene toward proteins makes this 2D material more biocompatible and less toxic as compared to graphene and has the potential to emerge as a better alternative to graphene for future biomedical applications.

#### 5.3.2 Hemoglobin

Understanding the behavior of nanosheets with blood proteins is an important aspect of evaluating its toxicity and biocompatibility. Interaction of black phosphorus nanosheets with hemoglobin protein has revealed the effect of this type of nanosheet on the structure and function of hemoglobin.^157^ The intrinsic fluorescence of hemoglobin was found to get quenched greatly upon binding with BP nanosheets, thereby showing that the nanosheet interacted directly with the protein. Forces such as vdW, hydrophobic, and electron transfer between the BP nanosheet surface and hemoglobin contributed toward the binding of hemoglobin with BP nanosheets. As a result of this binding interaction, the BP nanosheets caused extension of peptide chain and altered hydrophobicity around the aromatic amino acid residues of hemoglobin. However, BP nanosheets did not cause any conformational distortion to the heme moiety. CD spectroscopic studies revealed that the protein did not lose its native secondary structure and retained its structure in the main α-helix form. However, binding of hemoglobin to BP nanosheets altered the tertiary structure of the protein, as a result of which bilirubin binding to hemoglobin was compromised.

### 5.4 Proteins Interacting with MXenes

#### 5.4.1 Tyrosinase

Wu et al.^99^ studied immobilization of the enzyme tyrosinase on graphene-like MXene-Ti_2_C_3_ nanosheets and utilized this 2D material–protein interaction to develop an ultrasensitive electrochemical biosensor for detection of phenol. The large surface area of the nanosheets coupled with the presence of OH groups on the surface helped in attachment of the protein molecules on the surface of the nanosheets. The 2D nanomaterials were not only efficient in entrapping the enzyme but also retained their bioactivities, stability, and facilitated transport of enzyme substrate and products. FTIR studies of the secondary structure of the immobilized tyrosinase enzyme showed two vibrational bands of amide I and II. The amide I band arises from stretching of the carbonyl functional group of the peptide linkage in the protein backbone. The amide II band is due the combination of N–H and C–N stretching. A slight shift in the amide I band indicated the binding interactions between the enzyme and the nanosheets. Electrostatic interactions and hydrogen bonding between the enzyme and OH-terminated nanosheets were found to be the main forces acting between them. The excellent metallic conductivity of the MXene nanosheets coupled with its biocompatibility and good aqueous stability prompted the researchers to explore it as a matrix for immobilizing tyrosinase for fabrication of a biosensor. The MXene-based tyrosinase biosensor was highly efficient and sensitive in detecting very low concentrations of phenol in a short period of time. The biosensor could sense the analyte from 0.05 to 15.5 μmol L^–1^.It demonstrated a detection limit as low as 12 nmol L^–1^ and a sensitivity of 414.4 mA M^–1^. The biosensing approach was also found to be reproducible, stable, and able to detect phenol in real water samples.

#### 5.4.2 Horse Radish Peroxidase (HRP)

The excellent electrical conductivity of MXene nanosheets was also exploited by Bao-kai et al.^211^ for fabricating an electrochemical biosensor for detection of H_2_O_2_. MXene was used as the ideal substrate for immobilization of the enzyme HRP, a heme-containing enzyme which is widely used for catalyzing the oxidation of different kinds of substrates. The presence of vertical junction structure in MXene nanosheets improved HRP immobilization and showed better charge transfer properties. FTIR spectra of MXenes did not demonstrate any peak, but on the other hand HRP exhibited peaks at 2961, 1647, 1541, and 1080 cm^–1^. The presence of all the major bands corresponding to HRP in the HRP-immobilized MXene sample established that no major damage was incurred on the protein. The MXene-HRP biosensor showed good electrochemical behaviors and electrocatalytic activity toward reduction of H_2_O_2_. The biosensor demonstrated good analytical performance over a linear range from 5 μmol·L^–1^ to 1.650 mmol·L^–1^ and a low detection limit of 0.74 μmol·L^–1^. The biosensing approach was also found to be reproducible, stable, and useful for detection of trace level of H_2_O_2_ in both solid and liquid food products.

### 5.5 Protein Interacting with hBN

Interaction of insulin, an important metabolic hormone, with hexagonal boron nitride (BNNS) along with graphene monoxide (GMONS) and silicon carbide (SiCNS) nanosheets was studied by Atabay et al.^166^ They studied various parameters and analyzed the interaction of insulin with nanosheets. It was observed that with decrease in distance between insulin and nanosheet, there was enhancement in the interaction energy. The RMSD values were taken to study the stability of insulin on nanosheet surfaces. The results indicated that the structure of insulin was retained upon adsorption on the nanosheet surface. It was observed that insulin got adsorbed and immobilized through C-termini residues of chains A and B on BNNS, while the N-termini residues of chains A and B and C-termini residues of chain B were involved in interaction with GMONS. It can be said that role of vdW interaction was higher than the electrostatic interaction in total interaction energy. However, in BNNS, electrostatic bonding contributed more as compared to other nanosheets, owing to more polarity of BNNS. Solvent-accessible surface area (SASA) analysis was done to study the conformational study of insulin in different solvents, i.e., with water and after adsorption on nanosheets. It was inferred that the Phe–Phe binding, π–π stacking, and Cys–Cys sulfur bridge were important for adsorption and immobilization of insulin on BNNS and GMONS.

## 6 2D Nanomaterial–Protein Interactions: Conformational Changes and Denaturation

The adsorption of proteins onto 2D nanomaterials can have significant implications for disease development, primarily through the conformational changes in secondary structure or denaturation. Among the diseases extensively studied in relation to nanosheets is Alzheimer’s disease (AD). A critical step in AD involves the polymerization of amyloid-β peptide (Aβ) into amyloid fibrils with a β-sheet structure. The interaction between 2D nanomaterials and the monomeric peptide Aβ plays a crucial role in either promoting or inhibiting the proliferation of AD. Yang et al. investigated the interaction of graphene and GO with Aβ peptide and amyloid fibrils.^212^ The findings of their research demonstrated that graphene was able to dissolve preformed amyloid fibrils and inhibit the polymerization of the peptide. This effect was attributed to the sp^2^ carbon of graphene, which attracted peptides from the amyloid fibrils, resulting in a strong dispersion. Furthermore, the interaction was enhanced by π–π stacking between the aromatic residues of the peptide and graphene. The primary molecular mechanisms responsible for inhibiting AD included the penetration, insertion, and extraction of monomers from the fibrils. The findings obtained from AFM and thioflavin fluorescence assays demonstrated that both graphene and graphene oxide (GO) effectively disintegrated and cleared amyloid fibrils through their interaction with the monomer unit. Molecular dynamic studies were conducted, revealing that the interaction energy between the fibril and graphene decreased over time, while the contact area between the peptide and graphene increased. These observations suggest that graphene holds potential as an inhibitor of AD. The simulation results align with the experimental findings, indicating their potential usefulness in predicting the impact of 2D nanomaterial interactions with proteins in disease progression. Similarly, Li et al. demonstrated application of WS_2_ for inhibition of AD.^213^ The absorption of Aβ40 monomer onto WS_2_ nanosheets is facilitated by van der Waals and electrostatic interactions, effectively preventing its aggregation. Additionally, when near-infrared (NIR) light is applied to preformed Aβ aggregates in the presence of WS_2_, they dissociate due to the photothermal capability of WS_2_. The impact of WS_2_ was evaluated using AFM, revealing the presence of 1 μm sized fibrils in the absence of WS_2_, while no fibrils or aggregates were observed in its presence. CD spectra analysis indicated that the random coil structure of the monomer was retained in the presence of WS_2_, whereas in the absence of WS_2_, an increased β-sheet structure characteristic of fibrils was observed. ATR-FTIR spectroscopy further supported these findings, as the absence of a nanosheet showed the presence of amyloid fibrils, while a broader band between 1640 and 1645 cm^–1^ indicated a disordered conformation of the monomeric peptide in the presence of WS_2_. Thus, WS_2_ can also serve as an inhibitor of Alzheimer’s disease (AD) by interacting with the monomeric peptide.

Further, Yan et al. comprehensively studied the impact of graphene nanosheet interaction with spike protein of SARS-CoV-2 using molecular dynamics.^214^ The investigation indicated that in case of both wild type (WT) and omicron variant, graphene gets inserted in the pocket composed of N-terminal domain (NTD) and receptor binding site (RBD) of the spike protein in closed state, leading to their unavailability to bind angiotensin-converting enzyme 2 (ACE2) and thus inhibiting chances of infection. However, in open state spike protein, graphene fails to bind with RBD and allows RBD to interact with ACE-2, thus promoting infection which was found to be more detrimental in the case of omicron variant. Bisht et al. employed atomistic simulation and molecular docking to investigate the interaction between MoS_2_ nanosheet and RBD of spike protein of SARS-CoV-2.^206^ Their study revealed that MoS_2_ strongly binds to RBD via hydrogen, van der Waals and electrostatic bond, leading to conformation change of spike protein. This in turn leads to destabilizing of RBD-ACE2 interactions, thereby making it a potential antiviral candidate for battling COVID-19.

Wu et al. analyzed the impact of MoS_2_ nanosheet interaction with human islet amyloid peptide (hIAPP).^215^ Fibrillation of hIAPP is related to degenerative type 2 diabetes. Their experimental and modeling results showed that MoS_2_ nanosheet strongly interacts with monomer, dimer, and fibrils of hIAPP via van der Waals interactions. The binding is strong enough to retain the amyloid fibril structure in its own form, and also promoted fibrillation of peptide. The results suggested that MoS_2_ nanosheets can lead to proliferation of amyloid fibril-based disease like type 2 diabetes on interacting with hIAPP and other amyloid fibrils. Based on the above-mentioned examples, it can be inferred that the interaction between nanosheets and proteins can exhibit both inhibitory and proliferative effects on disease outcomes, depending on the nature of the interaction. The analytical techniques discussed in this review, such as microscopy and spectroscopic techniques, provide valuable means to study these interactions. Additionally, theoretical investigations employing molecular dynamics simulations can be utilized to predict and analyze the influence of nanosheets on proteins in the context of disease progression.

## 7 2D Nanomaterial for Protein Identification and Sensing

In the preceding discussions, we explored the intricate ways in which 2D nanomaterials interact with proteins. These interactions, which can be thoroughly examined and deciphered using various analytical tools, hold significant promise for applications in protein identification and sensing. Wu et al. developed an electrochemical immunosensor for detection of two biomarkers of cervical cancer, i.e., carcinoembryonic antigen (CEA) and squamous cell carcinoma antigen (SCCA), using tetraethylene pentaamine modified rGO.^216^ The modified rGO sheets were used to immobilize primary antibody (Ab_1_), whereas two secondary antibodies (Ab_2_) and different redox probes (neutral red and thionine) were loaded onto Au@mesoporous carbon CMK-3 nanoparticles for fabricating the immunosensor. The presence of respective biomarkers resulted in a peak current change of the redox probes which was detected as a signal. The immunosensor had a wide linear range, low detection limit, good reproducibility, and stability. A highly sensitive, label-free biosensing platform was developed by Singh et al. for detecting the cancer biomarker carcinoembryonic antigen (CEA) using graphene on a Cu substrate as the sensor platform and electrochemical impedance spectroscopy (EIS) as the sensing technique.^217^ The biosensor showed excellent sensitivity and specificity toward CEA with a linear response in the physiological range of 1.0–25.0 ng/mL, the sensitivity of 563.4 Ω ng^–1^ mL cm^–2^ and the limit of detection (LOD) of 0.23 ng/mL. Jin et al. also developed a graphene based electrochemical biosensor for CEA detection comprising of HRP, magnetic beads and AuNP that achieved a limit of detection of 5 ng/mL.^218^ An ultrasensitive electrochemical immunosensor was developed by Wang et al. to detect lung cancer biomarkers by employing reversible addition–fragmentation chain-transfer (RAFT) polymerization as a tool for signal amplification.^219^ A GO coated GCE electrode was used as the sensor base onto which primary antibody (Ab_1_) against the lung cancer biomarker CYFRA21–1 was immobilized. The secondary antibody against CYFRA21–1 conjugated with a chain transfer agent and used as the detection probe. In the presence of the biomarker, a sandwich-like immunocomplex was generated connected to many monomers via RAFT polymerization. It was observed that presence of GO and RAFT polymerization together contributed in improving the analytical performance of the biosensor.

Cao designed a nanocomplex consisting of graphene, MoS_2_, AuNP, chitosan, and HRP which were assembled on a glassy carbon electrode (GCE) for detection of DNA.^220^ The biosensor could detect DNA with a high selectivity and at concentrations as low as 2.2 × 10^–15^ M. Haung et al. prepared an electrochemical sensor based on cysteine assisted graphene–MoS_2_ composite.^221^ The sensor could detect various biomolecules including ascorbic acid, L-tryptophan, dopamine, and acetaminophen, with a low detection limit of 2.0 × 10^–8^ M (for acetaminophen). This sensor is a perfect example of 2D material-based sensors for protein detection. Wu et al. synthesized reduced MoS_2_ that can selectively interact with dopamine in a mixture of ascorbic acid, uric acid, and dopamine owing to the electrostatic interaction between positively charged dopamine and negatively charged GCE-APTES-rMoS_2_.^222^ Dopamine detection has also been done by Su et al., where they used gold nanoparticles decorated MoS_2_ as biosensor.^223^ Zhao et al. demonstrated a “turn-on” fluorescence biosensor based on fluorescently labeled protein aptamers and MoS_2_ nanosheets for the rapid and precise detection of carcinoembryonic antigen (CEA) protein.^224^ The CEA aptamer probe can be adsorbed on the surface of nearby MoS_2_ nanosheets via the van der Waals force, leading to energy transfer by FRET and quenching of the aptamer probe’s fluorescence signal. Due to the aptamer probe’s capacity to dissociate from MoS_2_ nanosheets with binding-induced conformation change, the fluorescence signal was recovered while CEA protein was still present. The remarkable quenching efficiency of the MoS_2_ nanosheets and their ability to differentiate between aptamers and aptamer/protein enabled the rapid and highly sensitive detection of the CEA protein biomarker. Kong et al. also designed an aptamer functionalized MoS_2_ based biosensor for early detection of PSA with significantly low value of 0.2 ng/mL as detection limit.^225^ Another study by Wang et al. employed the drain current of MoS_2_ nanosheets for detection of PSA, with a picomolar detection limit.^226^

In addition to graphene-based materials and TMDCs, the newly emerging MXene family of 2D nanomaterials have also been explored for designing biosensors. Wu et al. designed a SPR biosensor that incorporates an N-Ti_3_C_2_-MXene nanosheet-modified sensing platform as well as a signal enhancer made of N-Ti_3_C_2_-MXene, hollow gold nanoparticles (HGNPs), and staphylococcal protein A (SPA) complexes.^227^ A hydrophilic, biocompatible nanoplatform consisting of ultrathin Ti_3_C_2_-MXene nanosheets functionalized with aminosilane was used to covalently immobilize the monoclonal anti-CEA capture antibody (Ab1). The polyclonal anti-CEA detection antibody (Ab2) was immobilized on N-Ti_3_C_2_-MXene/HGNPs nanohybrids. The binding of CEA and subsequent formation of the sandwiched Ab2-conjugated SPA/HGNPs/N-Ti_3_C_2_-MXene nanocomplex on the SPR chip resulted in the generation of a response signal. The constructed N-Ti_3_C_2_-MXene-based SPR biosensor demonstrated a linear detection range of 0.001–1000 pM for CEA with a detection limit of 0.15 fM.

Xenes, such as black phosphorus or phosphorene, have also been exploited for designing biosensors. Gold nanoparticle-decorated phosphorene demonstrated excellent 4-nitrophenol (4-NP)-reduction catalytic activity, providing a colorimetric signal output from yellow 4-NP to colorless 4-aminophenol (4-AP), enabling the detection of the carcinoembryonic antigen biomarker as a highly selective and sensitive colorimetric method in the clinical samples obtained from breast and colon cancer patients.^228^

## 8 Environmental Impact of 2D Nanomaterials

2D nanomaterials have extensive applications across various industries and research fields. However, these nanomaterials often have a tendency to aggregate and form nontoxic bulk counterparts over time when released into the environment. The entry of these materials into the environment can occur through air, water, or soil. The environmental impact of graphene derivatives is contingent upon their behavior and transformations within the surrounding media. While graphene derivatives can persist in the air for extended periods, factors such as sunlight or strong forces can induce aggregation, reduction, or structural degradation of the nanosheets.^229,230^ In soil, graphene can be decomposed by soil microbiota, for instance, a study reported oxidation of graphene and mitigation of carbon content of rGO on interaction with *Phanerochaetes chrysosporium*.^231^ It has the ability to fix heavy metals in soil like Cr with surface complexation and electrostatic interaction, besides the redox and biological decomposition occurring in the soil. Cumulative effect of all these factors on graphene nanosheets is also seen in aquatic environment, where sunlight, pollutants, microorganisms, and minerals can interact. These interactions can lead to removal of toxic substances by conversion to less toxic forms or accumulation of heavy metals by adsorption or transformation in nanosheets into colloids, aggerates, other chemical forms. Wang et al. and Cao et al. in separate studies found that graphene nanosheet enhanced arsenite (As) accumulation and thus its toxicity by increasing its bioavailability.^232,233^ The accumulated As altered the metabolism of the aquatic organism like zebrafish. The impact of pollutants like zinc and cadmium in the presence of GO was studied on the freshwater fish *Geophagus iporangensis*, and an alteration in the metabolic rate and route of the fish was observed.^234^ Graphene and its derivatives can also mitigate to higher food levels, thus more research is required in analyzing the fate of nanosheets in the environment.

Similarly, TMDCs are also environmentally less detrimental owing to their chemical stability in ambient environment. The absence of dangling bonds in the terminating chalcogen atoms makes them more stable. TMDCs are not prone to oxidation in normal environmental conditions unless extreme conditions like heat or strong oxidation are applied.^235^ However, Wang et al. showed that in aqueous media they are prone to oxidation which results in release of soluble molybdenum and sulfur species.^236^ This is also accompanied by release of protons which contributes toward further destabilization of the nanosheets leading to degradation. The degradation of MoS_2_ was also found to be pH-dependent, with higher oxidation kinetics reaching at higher pH.^236^ Chemically exfoliated MoS_2_ nanosheets showed rapid oxidation in environmental media with a half-life of 30 days, whereas ultrasonically exfoliated nanosheets showed slow oxidation. Experiments carried out in biological media demonstrated that MoS_2_ nanosheets did not show long-term persistence in living systems and oxic natural waters. MoS_2_ leads to the formation of molybdenum oxide (MoO_3_) at high temperature of 340 °C, with oxidation starting at defect sites.^237^ In the case of WSe_2_, the oxidation initiates from edges and grows inward at comparable temperature.^238^ The oxidation rate is temperature-dependent, with less oxidation occurring at low temperatures, having negligible impact at 200 °C.

MXenes have the potential to remove heavy metals from the aquatic environment by adsorption.^239^ It may enter the environment via agricultural applications, packaging, cosmetics, and biomedicines. Bury et al. studied the role of MXenes in bioremediation, by removal of various gaseous and organic pollutants.^240,241^ To date, studies have indicated the toxicity of MXenes toward bacteria and cancer cells, while no adverse effects on normal human cells have been reported. As a relatively new class of 2D nanomaterials, there is limited research on the long-term environmental effects of MXenes. However, available literature suggests that MXenes could potentially impact aquatic organisms, and in the case of terrestrial organisms, the respiratory and digestive tracts appear to be more susceptible to these effects.^242^ It should be noted that comprehensive investigations on the environmental fate of hBN and Xenes are currently lacking in the existing scientific literature. In summary, it can be concluded that 2D nanomaterials undergo changes in their structure, morphology, and surface properties when exposed to the environment. The long-term behavior of these nanomaterials in the environment is influenced by various factors, including physicochemical parameters such as pH, temperature, surface functionalization, and exposure to sunlight. Additionally, natural environmental factors such as chemicals, pollutants, and organisms also play a role in determining whether the ultimate outcome is detrimental or safe.

## 9 Conclusion

The emergence of 2D nanomaterials has been a boon to the field of nanotechnology. Once thought to be unstable in the free form, these nanosheets are now finding applications in wide spectra of fields including optics, electronics, chemical, biotechnology and biomedical. Different types of 2D nanomaterials are available now, like carbon-based graphene, GO (GO), and reduced graphene based (RGO); TMDCs like MoS_2_ and WS_2_; Xenes, MXenes, and hexagonal boron nitride. Out of these, carbon based and TMDCs are most explored nanosheets in the field of biomedical research. The hexagonal structure, large surface area, sp^2^ and sp^3^ hybridization, and single-atom-thin sheet favors the binding of proteins to these nanosheets. Albumin (BSA and HSA) tend to adsorb on both graphene-based and TMDC nanosheets, without much impact on its secondary structure. Other plasma proteins like Fg, globulin, etc., get adsorbed with varying tendency on the nanosheets. Enzymatic activity of trypsin and chymotrypsin can be inhibited by adsorption on graphene and GO, besides graphene prevents thermal denaturation of trypsin. After studying interaction of different proteins with 2D nanomaterials, we can say that majority of the proteins get adsorbed on nanosheets through a combination of different forces such as π–π stacking, hydrophobic bonding, hydrophilic bonding, electrostatic interactions, and vdW forces. Some other forces can also be involved, like disulfide bonds in the case of interaction with TMDCs and functionalization of nanosheets with molecules like PEG, and can favor covalent bonding (functionalized nanosheets not discussed).

Analysis of protein adsorption on nanosheet and characterization of the nature of interaction can be done through various analytical techniques: for example, microscopic techniques like AFM, SEM, and TEM can be explored to image the protein bound nanosheets; hydrodynamic sizes and surface potential can be measured using DLS and zeta potential; and concentration-dependent measurement can be done through various spectroscopic techniques like UV–vis and fluorescence spectroscopy. Proteins have intrinsic fluorescence due to the presence of tryptophan, tyrosine, and phenylalanine residues that gets quenched on adsorption onto nanosheet, and energy transfer from protein to nanosheet or vice versa takes place on adsorption. These quenching and FRET approaches can be utilized using fluorescence spectroscopy to give information about the protein–nanosheet interaction. As the protein adsorbs on nanosheet, there are chances of conformational changes and protein denaturation. Any change in secondary structure of proteins can be estimated using FT-IR and CD spectroscopy. The interactions at buried surfaces can be examined using SFG spectra; chemical and elemental analysis can be done using XPS, EDX, XRD, and Raman spectroscopy. Important thermodynamic parameters of the interactions like binding association, dissociation constants, and enthalpies can be measured using ITC techniques. Further study of the interactions at atomic level or the ones not possible experimentally can be theoretically studied using modeling. MD simulation can be used to study theoretical model of proteins and nanosheets to obtain important information like nature of interaction, time-dependent contact area, time-dependent energy change, and many other parameters.

With the help of these techniques, we can understand the impact of adsorption on protein activity, which further helps to determine the compatibility of these nanosheets. Despite many advantages, there are few shortcomings especially when biological applications are considered. As the protein gets adsorbed on the nanosheet, its binding site may get affected, secondary structure may be altered, and activity can be changed. This may cause toxicity when introduced in the living system. Besides this, there is a lack of standardized quality evaluation system of 2D nanomaterials established by an official organization or committee, which can ensure reproduction of results in different laboratories. Further, the lack of long-term *in vivo* studies regarding the fate of nanosheets in biosystem is itself a big limitation.^243,244^ Some nanosheets may induce cytotoxicity, which may get mitigated or enhanced by the adsorption of proteins. There has been a tremendous debate on the impact of protein bound nanosheet on toxicity, with some research showing that proteins help in mitigating the toxicological effect that would have been there when nascent nanosheets are used, while others establishing that protein adsorption enhance the toxicity or generate stronger immune response against the nanosheets. Future studies should take into consideration the effect of 2D material size, composition and surface properties on different biological entities, such as proteins to better understand the biological consequences and nanotoxicity of this class of material. Conclusively, we can say that 2D nanomaterials provide a compatible surface for protein adsorption, where proteins adsorb through various interactions, and the outcome of such interactions are different for different types of proteins as well as nanosheets, which ultimately decide the fate of these nanomaterials in the context of biomedical applications.

## References

[ref1] Novoselov K. S.; Geim A. K.; Morozov S. V.; Jiang D.-e.; Zhang Y.; Dubonos S. V.; Grigorieva I. V.; Firsov A. A. Electric Field Effect In Atomically Thin Carbon Films. Science 2004, 306 (5696), 666–669. 10.1126/science.1102896. 15499015

[ref2] Zhu C.; Du D.; Lin Y. Graphene-Like 2D Nanomaterial-Based Biointerfaces for Biosensing Applications. Biosens. Bioelectrons 2017, 89, 43–55. 10.1016/j.bios.2016.06.045. PMC12998984 27373809

[ref3] Sun X.; Liu Z.; Welsher K.; Robinson J. T.; Goodwin A.; Zaric S.; Dai H. Nano-Graphene Oxide for Cellular Imaging and Drug Delivery. Nano Research 2008, 1 (3), 203–212. 10.1007/s12274-008-8021-8. 20216934 PMC2834318

[ref4] Wang K.; Ruan J.; Song H.; Zhang J.; Wo Y.; Guo S.; Cui D. Biocompatibility of Graphene Oxide. Nanoscale Res. Lett. 2011, 6, 8 10.1007/s11671-010-9751-6. 27502632 PMC3212228

[ref5] Zhang B.; Wei P.; Zhou Z.; Wei T. Interactions of Graphene with Mammalian Cells: Molecular Mechanisms and Biomedical Insights. Adv. Drug Delivery Rev. 2016, 105, 145–162. 10.1016/j.addr.2016.08.009. 27569910

[ref6] Liu Z.; Robinson J. T.; Sun X.; Dai H. PEGylated Nanographene Oxide for Delivery of Water-Insoluble Cancer Drugs. J. Am. Chem. Soc. 2008, 130 (33), 10876–10877. 10.1021/ja803688x. 18661992 PMC2597374

[ref7] Zhang L.; Xia J.; Zhao Q.; Liu L.; Zhang Z. Functional Graphene Oxide as a Nanocarrier for Controlled Loading and Targeted Delivery of Mixed Anticancer Drugs. Small 2010, 6 (4), 537–544. 10.1002/smll.200901680. 20033930

[ref8] Weaver C. L.; LaRosa J. M.; Luo X.; Cui X. T. Electrically Controlled Drug Delivery from Graphene Oxide Nanocomposite Films. ACS Nano 2014, 8 (2), 1834–1843. 10.1021/nn406223e. 24428340 PMC4004293

[ref9] Chitgupi U.; Qin Y.; Lovell J. F. Targeted Nanomaterials for Phototherapy. Nanotheranostics 2017, 1 (1), 38 10.7150/ntno.17694. 29071178 PMC5646723

[ref10] Cheng L.; Wang C.; Feng L.; Yang K.; Liu Z. Functional Nanomaterials for Phototherapies of Cancer. Chem. Rev. 2014, 114 (21), 10869–10939. 10.1021/cr400532z. 25260098

[ref11] Tan C.; Cao X.; Wu X.-J.; He Q.; Yang J.; Zhang X.; Chen J.; Zhao W.; Han S.; Nam G.-H.; et al. Recent Advances In Ultrathin Two-Dimensional Nanomaterials. Chem. Rev. 2017, 117 (9), 6225–6331. 10.1021/acs.chemrev.6b00558. 28306244

[ref12] Lynch I.; Dawson K. A. Protein-Nanoparticle Interactions. Nano Today 2008, 3 (1–2), 40–47. 10.1016/S1748-0132(08)70014-8.

[ref13] Nel A. E.; Mädler L.; Velegol D.; Xia T.; Hoek E. M.; Somasundaran P.; Klaessig F.; Castranova V.; Thompson M. Understanding Biophysicochemical Interactions at the Nano–Bio Interface. Nat. Mater. 2009, 8 (7), 543–557. 10.1038/nmat2442. 19525947

[ref14] Chithrani B. D.; Chan W. C. Elucidating the Mechanism of Cellular Uptake and Removal of Protein-Coated Gold Nanoparticles of Different Sizes and Shapes. Nano Lett. 2007, 7 (6), 1542–1550. 10.1021/nl070363y. 17465586

[ref15] Lesniak A.; Campbell A.; Monopoli M. P.; Lynch I.; Salvati A.; Dawson K. A. Serum Heat Inactivation Affects Protein Corona Composition and Nanoparticle Uptake. Biomaterials 2010, 31 (36), 9511–9518. 10.1016/j.biomaterials.2010.09.049. 21059466

[ref16] Donahue N. D.; Acar H.; Wilhelm S. Concepts of Nanoparticle Cellular Uptake, Intracellular Trafficking, and Kinetics in Nanomedicine. Adv. Drug Delivery Rev. 2019, 143, 68–96. 10.1016/j.addr.2019.04.008. 31022434

[ref17] Russier J.; Treossi E.; Scarsi A.; Perrozzi F.; Dumortier H.; Ottaviano L.; Meneghetti M.; Palermo V.; Bianco A. Evidencing the Mask Effect of Graphene Oxide: A Comparative Study on Primary Human and Murine Phagocytic Cells. Nanoscale 2013, 5 (22), 11234–11247. 10.1039/c3nr03543c. 24084792

[ref18] Moore C.; Movia D.; Smith R. J.; Hanlon D.; Lebre F.; Lavelle E. C.; Byrne H. J.; Coleman J. N.; Volkov Y.; McIntyre J. Industrial Grade 2D Molybdenum Disulphide (MoS2): An In Vitro Exploration of the Impact on Cellular Uptake, Cytotoxicity, and Inflammation. 2D Materials 2017, 4 (2), 025065 10.1088/2053-1583/aa673f.

[ref19] Li Y.; Yuan H.; von Dem Bussche A.; Creighton M.; Hurt R. H.; Kane A. B.; Gao H. Graphene Microsheets Enter Cells Through Spontaneous Membrane Penetration at Edge Asperities and Corner Sites. Proc. Natl. Acad. Sci. U. S. A. 2013, 110 (30), 12295–12300. 10.1073/pnas.1222276110. 23840061 PMC3725082

[ref20] Kostarelos K.; Novoselov K. S. Exploring the Interface of Graphene and Biology. Science 2014, 344 (6181), 261–263. 10.1126/science.1246736. 24744363

[ref21] Ruiz O. N.; Fernando K. S.; Wang B.; Brown N. A.; Luo P. G.; McNamara N. D.; Vangsness M.; Sun Y.-P.; Bunker C. E. Graphene Oxide: A Nonspecific Enhancer of Cellular Growth. ACS Nano 2011, 5 (10), 8100–8107. 10.1021/nn202699t. 21932790

[ref22] Lim G. P.; Soon C. F.; Ma N. L.; Morsin M.; Nayan N.; Ahmad M. K.; Tee K. S. Cytotoxicity of MXene-Based Nanomaterials for Biomedical Applications: A Mini Review. Environ. Res. 2021, 201, 111592 10.1016/j.envres.2021.111592. 34175291

[ref23] Santos J.; Moschetta M.; Rodrigues J.; Alpuim P.; Capasso A. Interactions Between 2D Materials and Living Matter: A Review on Graphene and Hexagonal Boron Nitride Coatings. Frontiers in Bioengineering & Biotechnology 2021, 9, 612669 10.3389/fbioe.2021.612669. 33585432 PMC7873463

[ref24] Roy S.; Deo K.; Singh K. A.; Lee H. P.; Jaiswal A.; Gaharwar A. K. Nano-Bio Interactions of 2D Molybdenum Disulfide. Adv. Drug Delivery Rev. 2022, 187, 114361 10.1016/j.addr.2022.114361. PMC12861116 35636569

[ref25] Tao W.; Kong N.; Ji X.; Zhang Y.; Sharma A.; Ouyang J.; Qi B.; Wang J.; Xie N.; Kang C.; et al. Emerging Two-Dimensional Monoelemental Materials (Xenes) for Biomedical Applications. Chem. Soc. Rev. 2019, 48 (11), 2891–2912. 10.1039/C8CS00823J. 31120049

[ref26] Perkins F. K.; Friedman A. L.; Cobas E.; Campbell P.; Jernigan G.; Jonker B. T. Chemical Vapor Sensing with Monolayer MoS2. Nano Lett. 2013, 13 (2), 668–673. 10.1021/nl3043079. 23339527

[ref27] Sarkar D.; Liu W.; Xie X.; Anselmo A. C.; Mitragotri S.; Banerjee K. MoS2 Field-Effect Transistor for Next-Generation Label-Free Biosensors. ACS Nano 2014, 8 (4), 3992–4003. 10.1021/nn5009148. 24588742

[ref28] Zhu X.; Ji X.; Kong N.; Chen Y.; Mahmoudi M.; Xu X.; Ding L.; Tao W.; Cai T.; Li Y.; et al. Intracellular Mechanistic Understanding of 2D MoS2 Nanosheets for Anti-Exocytosis-Enhanced Synergistic Cancer Therapy. ACS Nano 2018, 12 (3), 2922–2938. 10.1021/acsnano.8b00516. 29406760 PMC6097229

[ref29] Fan H.; Zhao D.; Li Y.; Zhou J. Lysozyme Orientation and Conformation on MoS2 Surface: Insights from Molecular Simulations. Biointerphases 2017, 12 (2), 02D416 10.1116/1.4984803. PMC5457294 28576080

[ref30] Roy S.; Jaiswal A. Graphene-Based Nanomaterials for Theranostic Applications. Reports in Advances of Physical Sciences 2017, 1 (04), 1750011 10.1142/S2424942417500116.

[ref31] Roy S.; Mondal A.; Yadav V.; Sarkar A.; Banerjee R.; Sanpui P.; Jaiswal A. Mechanistic Insight into the Antibacterial Activity of Chitosan Exfoliated MoS2 Nanosheets: Membrane Damage, Metabolic Inactivation, and Oxidative Stress. ACS Appl. Bio Mater. 2019, 2 (7), 2738–2755. 10.1021/acsabm.9b00124. 35030809

[ref32] Roy S.; Sarkar A.; Jaiswal A. Poly (allylamine Hydrochloride)-Functionalized Reduced Graphene Oxide for Synergistic Chemo-Photothermal Therapy. Nanomedicine 2019, 14 (3), 255–274. 10.2217/nnm-2018-0320. 30676277

[ref33] Yadav V.; Roy S.; Singh P.; Khan Z.; Jaiswal A. 2D MoS2-Based Nanomaterials for Therapeutic, Bioimaging, and Biosensing Applications. Small 2019, 15 (1), 1803706 10.1002/smll.201803706. 30565842

[ref34] Kumar P.; Roy S.; Sarkar A.; Jaiswal A. Reusable MoS2-Modified Antibacterial Fabrics With Photothermal Disinfection Properties for Repurposing of Personal Protective Masks. ACS Appl. Mater. Interfaces 2021, 13 (11), 12912–12927. 10.1021/acsami.1c00083. 33715350

[ref35] Rosli N. F.; Mayorga-Martinez C. C.; Latiff N. M.; Rohaizad N.; Sofer Z. k.; Fisher A. C.; Pumera M. Layered Ptte2Matches Electrocatalytic Performance of Pt/C for Oxygen Reduction Reaction with Significantly Lower Toxicity. ACS Sustainable Chem. Eng. 2018, 6 (6), 7432–7441. 10.1021/acssuschemeng.7b04920.

[ref36] Cai Z.; Liu B.; Zou X.; Cheng H.-M. Chemical Vapor Deposition Growth and Applications of Two-Dimensional Materials and Their Heterostructures. Chem. Rev. 2018, 118 (13), 6091–6133. 10.1021/acs.chemrev.7b00536. 29384374

[ref37] Demirel M. C.; Vural M.; Terrones M. Composites of Proteins and 2D Nanomaterials. Adv. Funct. Mater. 2018, 28 (27), 1704990 10.1002/adfm.201704990.

[ref38] Bernardi M.; Palummo M.; Grossman J. C. Extraordinary Sunlight Absorption and One Nanometer Thick Photovoltaics Using Two-Dimensional Monolayer Materials. Nano Lett. 2013, 13 (8), 3664–3670. 10.1021/nl401544y. 23750910

[ref39] Zhang W.; Chuu C.-P.; Huang J.-K.; Chen C.-H.; Tsai M.-L.; Chang Y.-H.; Liang C.-T.; Chen Y.-Z.; Chueh Y.-L.; He J.-H.; et al. Ultrahigh-Gain Photodetectors Based on Atomically Thin Graphene-MoS2 Heterostructures. Sci. Rep. 2014, 4, 3826 10.1038/srep03826. 24451916 PMC3899643

[ref40] Lopez-Sanchez O.; Lembke D.; Kayci M.; Radenovic A.; Kis A. Ultrasensitive Photodetectors Based on Monolayer MoS2. Nat. Nanotechnol. 2013, 8 (7), 497–501. 10.1038/nnano.2013.100. 23748194

[ref41] Yang X.; Cheng C.; Wang Y.; Qiu L.; Li D. Liquid-Mediated Dense Integration of Graphene Materials for Compact Capacitive Energy Storage. Science 2013, 341 (6145), 534–537. 10.1126/science.1239089. 23908233

[ref42] Tao Y.; Xie X.; Lv W.; Tang D.-M.; Kong D.; Huang Z.; Nishihara H.; Ishii T.; Li B.; Golberg D.; et al. Towards Ultrahigh Volumetric Capacitance: Graphene Derived Highly Dense but Porous Carbons for Supercapacitors. Sci. Rep. 2013, 3, 2975 10.1038/srep02975. 24131954 PMC3797987

[ref43] Armano A.; Agnello S. Two-Dimensional Carbon: A Review of Synthesis Methods, and Electronic, Optical, and Vibrational Properties of Single-Layer Graphene. C 2019, 5 (4), 67 10.3390/c5040067.

[ref44] Murali A.; Lokhande G.; Deo K. A.; Brokesh A.; Gaharwar A. K. Emerging 2D Nanomaterials for Biomedical Applications. Mater. Today 2021, 50, 276–302. 10.1016/j.mattod.2021.04.020. PMC8713997 34970073

[ref45] Soldano C.; Mahmood A.; Dujardin E. Production, Properties and Potential of Graphene. Carbon 2010, 48 (8), 2127–2150. 10.1016/j.carbon.2010.01.058.

[ref46] Li D.; Shao Z.-G.; Hao Q.; Zhao H. Intrinsic Carrier Mobility of a Single-Layer Graphene Covalently Bonded With Single-Walled Carbon Nanotubes. Journal of applied physics 2014, 115 (23), 233701 10.1063/1.4883759.

[ref47] Lee C.; Wei X.; Kysar J. W.; Hone J. Measurement of the Elastic Properties and Intrinsic Strength of Monolayer Graphene. Science 2008, 321 (5887), 385–388. 10.1126/science.1157996. 18635798

[ref48] Nair R. R.; Blake P.; Grigorenko A. N.; Novoselov K. S.; Booth T. J.; Stauber T.; Peres N. M.; Geim A. K. Fine Structure Constant Defines Visual Transparency of Graphene. Science 2008, 320 (5881), 1308–1308. 10.1126/science.1156965. 18388259

[ref49] Liu F.; Ming P.; Li J. Ab Initio Calculation of Ideal Strength and Phonon Instability of Graphene Under Tension. Phys. Rev. B 2007, 76 (6), 064120 10.1103/PhysRevB.76.064120.

[ref50] Zhang Y.; Wu C.; Guo S.; Zhang J. Interactions of Graphene and Graphene Oxide With Proteins and Peptides. Nanotechnol. Rev. 2013, 2 (1), 27–45. 10.1515/ntrev-2012-0078.

[ref51] Geetha Bai R.; Muthoosamy K.; Manickam S.; Hilal-Alnaqbi A. Graphene-Based 3D Scaffolds in Tissue Engineering: Fabrication, Applications, and Future Scope in Liver Tissue Engineering. Int. J. Nanomed. 2019, 14, 5753 10.2147/IJN.S192779. PMC6662516 31413573

[ref52] Cham sa-ard W.; Fawcett D.; Fung C. C.; Chapman P.; Rattan S.; Poinern G. E. J. Synthesis, Characterisation and Thermo-Physical Properties of Highly Stable Graphene Oxide-Based Aqueous Nanofluids for Potential Low-Temperature Direct Absorption Solar Applications. Sci. Rep. 2021, 11 (1), 1–13. 10.1038/s41598-021-94406-y. 34400658 PMC8367989

[ref53] Poulin P.; Jalili R.; Neri W.; Nallet F.; Divoux T.; Colin A.; Aboutalebi S. H.; Wallace G.; Zakri C. Superflexibility of Graphene Oxide. Proc. Natl. Acad. Sci. U. S. A. 2016, 113 (40), 11088–11093. 10.1073/pnas.1605121113. 27647890 PMC5056031

[ref54] Radisavljevic B.; Radenovic A.; Brivio J.; Giacometti V.; Kis A. Single-layer MoS2 Transistors. Nat. Nanotechnol. 2011, 6 (3), 147–150. 10.1038/nnano.2010.279. 21278752

[ref55] Zhang Y.; Xiu W.; Gan S.; Shan J.; Ren S.; Yuwen L.; Weng L.; Teng Z.; Wang L. Antibody-Functionalized MoS2 Nanosheets for Targeted Photothermal Therapy of *Staphylococcus aureus* Focal Infection. Front. Bioeng. Biotechnol. 2019, 7, 218 10.3389/fbioe.2019.00218. 31552242 PMC6746923

[ref56] Lin Y.; Connell J. W. Advances in 2D Boron Nitride Nanostructures: Nanosheets, Nanoribbons, Nanomeshes, and Hybrids With Graphene. Nanoscale 2012, 4 (22), 6908–6939. 10.1039/c2nr32201c. 23023445

[ref57] Zhang B.; Wu Q.; Yu H.; Bulin C.; Sun H.; Li R.; Ge X.; Xing R. High-Efficient Liquid Exfoliation of Boron Nitride Nanosheets Using Aqueous Solution of Alkanolamine. Nanoscale Res. Lett. 2017, 12 (1), 596 10.1186/s11671-017-2366-4. 29150793 PMC5691823

[ref58] Wang N.; Yang G.; Wang H.; Yan C.; Sun R.; Wong C.-P. A Universal Method for Large-Yield and High-Concentration Exfoliation of Two-Dimensional Hexagonal Boron Nitride Nanosheets. Mater. Today 2019, 27, 33–42. 10.1016/j.mattod.2018.10.039.

[ref59] Vishnoi P.; Pramoda K.; Rao C. 2D Elemental Nanomaterials beyond Graphene. ChemNanoMat 2019, 5 (9), 1062–1091. 10.1002/cnma.201900176.

[ref60] Ren X.; Li Z.; Huang Z.; Sang D.; Qiao H.; Qi X.; Li J.; Zhong J.; Zhang H. Environmentally Robust Black Phosphorus Nanosheets in Solution: Application for Self-Powered Photodetector. Adv. Funct. Mater. 2017, 27 (18), 1606834 10.1002/adfm.201606834.

[ref61] Suryawanshi S. R.; More M. A.; Late D. J. Laser Exfoliation of 2D Black Phosphorus Nanosheets and Their Application as a Field Emitter. RSC Adv. 2016, 6 (113), 112103–112108. 10.1039/C6RA24526A.

[ref62] Mondal K.; Ghosh P. Exfoliation of Ti2C and Ti3C2MXenes from Bulk Trigonal Phases of Titanium Carbide: A Theoretical Prediction. Solid State Commun. 2019, 299, 113657 10.1016/j.ssc.2019.113657.

[ref63] Eom W.; Shin H.; Ambade R. B.; Lee S. H.; Lee K. H.; Kang D. J.; Han T. H. Large-Scale Wet-Spinning of Highly Electroconductive MXene Fibers. Nat. Commun. 2020, 11 (1), 2825 10.1038/s41467-020-16671-1. 32499504 PMC7272396

[ref64] Dikin D. A.; Stankovich S.; Zimney E. J.; Piner R. D.; Dommett G. H.; Evmenenko G.; Nguyen S. T.; Ruoff R. S. Preparation and Characterization of Graphene Oxide Paper. Nature 2007, 448 (7152), 457–460. 10.1038/nature06016. 17653188

[ref65] Lee D. Y.; Khatun Z.; Lee J.-H.; Lee Y.-k.; In I. Blood Compatible Graphene/Heparin Conjugate Through Noncovalent Chemistry. Biomacromolecules 2011, 12 (2), 336–341. 10.1021/bm101031a. 21218769

[ref66] Hassan M.; Walter M.; Moseler M. Interactions of Polymers with Reduced Graphene Oxide: van der Waals Binding Energies of Benzene on Graphene with Defects. Phys. Chem. Chem. Phys. 2014, 16 (1), 33–37. 10.1039/C3CP53922A. 24226810

[ref67] Shen J.; Shi M.; Yan B.; Ma H.; Li N.; Hu Y.; Ye M. Covalent Attaching Protein to Graphene Oxide via Diimide-Activated Amidation. Colloids Surf., B 2010, 81 (2), 434–438. 10.1016/j.colsurfb.2010.07.035. 20728319

[ref68] Kou L.; He H.; Gao C. Click Chemistry Approach to Functionalize Two-Dimensional Macromolecules of Graphene Oxide Nanosheets. Nano-Micro Lett. 2010, 2 (3), 177–183. 10.1007/BF03353638.

[ref69] Simsikova M.; Sikola T. Interaction of Graphene Oxide with Proteins and Applications of their Conjugates. J. Nanomed. Res. 2017, 5 (2), 00109 10.15406/jnmr.2017.05.00109.

[ref70] Balasubramanyam S. Nanoengineering of Two-Dimensional WS2 by Atomic Layer Deposition. Ph.D. Thesis, Technische Universiteit Eindhoven, 2020.

[ref71] Shinde P. V.; Singh M. K. Synthesis, Characterization, and Properties of Graphene Analogs of 2D Material. In Fundamentals and Sensing Applications of 2D Materials; Elsevier, 2019; pp 91–143.

[ref72] Eftekhari A. Tungsten Dichalcogenides (WS2, WSe2, and WTe2): Materials Chemistry and Applications. J. Mater. Chem. A 2017, 5 (35), 18299–18325. 10.1039/C7TA04268J.

[ref73] Agarwal V.; Varghese N.; Dasgupta S.; Sood A.; Chatterjee K. Engineering a 3D MoS2 Foam using Keratin Exfoliated Nanosheets. Chemical Engineering Journal 2019, 374, 254–262. 10.1016/j.cej.2019.05.185.

[ref74] Jaiswal M. K.; Singh K. A.; Lokhande G.; Gaharwar A. K. Superhydrophobic States of 2D Nanomaterials Controlled by Atomic Defects can Modulate Cell Adhesion. Chem. Commun. 2019, 55 (60), 8772–8775. 10.1039/C9CC00547A. PMC7004258 31172998

[ref75] Tan C.; Zhang H. Two-dimensional Transition Metal Dichalcogenide Nanosheet-based Composites. Chem. Soc. Rev. 2015, 44 (9), 2713–2731. 10.1039/C4CS00182F. 25292209

[ref76] Chhowalla M.; Shin H. S.; Eda G.; Li L.-J.; Loh K. P.; Zhang H. The Chemistry of Two-Dimensional Layered Transition Metal Dichalcogenide Nanosheets. Nat. Chem. 2013, 5 (4), 263–275. 10.1038/nchem.1589. 23511414

[ref77] Xiao M.; Wei S.; Chen J.; Tian J.; Brooks C. L. Iii; Marsh E. N. G.; Chen Z. Molecular Mechanisms of Interactions between Monolayered Transition Metal Dichalcogenides and Biological Molecules. J. Am. Chem. Soc. 2019, 141 (25), 9980–9988. 10.1021/jacs.9b03641. 31199639

[ref78] Wang H.; Yu L.; Lee Y.-H.; Shi Y.; Hsu A.; Chin M. L.; Li L.-J.; Dubey M.; Kong J.; Palacios T. Integrated Circuits Based on Bilayer MoS2 Transistors. Nano Lett. 2012, 12 (9), 4674–4680. 10.1021/nl302015v. 22862813

[ref79] Enyashin A.; Ivanovskii A. Graphene-like BN Allotropes: Structural and Electronic Properties from DFTB Calculations. Chem. Phys. Lett. 2011, 509 (4–6), 143–147. 10.1016/j.cplett.2011.04.081.

[ref80] Sharker S. M. Hexagonal Boron Nitrides (White Graphene): A Promising Method for Cancer Drug Delivery. Int. J. Nanomed. 2019, 14, 9983 10.2147/IJN.S205095. PMC6927571 31908454

[ref81] Anees P.; Valsakumar M.; Panigrahi B. Effect of Strong Phonon–Phonon Coupling on the Temperature Dependent Structural Stability and Frequency Shift of 2D Hexagonal Boron Nitride. Phys. Chem. Chem. Phys. 2016, 18 (4), 2672–2681. 10.1039/C5CP06111C. 26705543

[ref82] Kumar R.; Rajasekaran G.; Parashar A. Optimised Cut-off Function for Tersoff-like Potentials for a BN Nanosheet: A Molecular Dynamics Study. Nanotechnology 2016, 27 (8), 085706 10.1088/0957-4484/27/8/085706. 26820110

[ref83] Zhou H.; Zhu J.; Liu Z.; Yan Z.; Fan X.; Lin J.; Wang G.; Yan Q.; Yu T.; Ajayan P. M.; et al. High Thermal Conductivity of Suspended Few-Layer Hexagonal Boron Nitride Sheets. Nano Res. 2014, 7 (8), 1232–1240. 10.1007/s12274-014-0486-z.

[ref84] Dean C. R.; Young A. F.; Meric I.; Lee C.; Wang L.; Sorgenfrei S.; Watanabe K.; Taniguchi T.; Kim P.; Shepard K. L.; et al. Boron Nitride Substrates for High-Quality Graphene Electronics. Nat. Nanotechnol. 2010, 5 (10), 722–726. 10.1038/nnano.2010.172. 20729834

[ref85] Alwarappan S.; Boyapalle S.; Kumar A.; Li C.-Z.; Mohapatra S. Comparative Study of Single-, Few-, and Multilayered Graphene Toward Enzyme Conjugation and Electrochemical Response. J. Phys. Chem. C 2012, 116 (11), 6556–6559. 10.1021/jp211201b.

[ref86] Li L. H.; Chen Y. Atomically Thin Boron Nitride: Unique Properties and Applications. Adv. Funct. Mater. 2016, 26 (16), 2594–2608. 10.1002/adfm.201504606.

[ref87] Li L. H.; Santos E. J.; Xing T.; Cappelluti E.; Roldán R.; Chen Y.; Watanabe K.; Taniguchi T. Dielectric Screening in Atomically Thin Boron Nitride Nanosheets. Nano Lett. 2015, 15 (1), 218–223. 10.1021/nl503411a. 25457561

[ref88] Zhang K.; Feng Y.; Wang F.; Yang Z.; Wang J. Two Dimensional Hexagonal Boron Nitride (2D-HBn): Synthesis, Properties and Applications. J. Mater. Chem. C 2017, 5 (46), 11992–12022. 10.1039/C7TC04300G.

[ref89] Chen L.; Xu H.-F.; He S.-J.; Du Y.-H.; Yu N.-J.; Du X.-Z.; Lin J.; Nazarenko S. Thermal Conductivity Performance of Polypropylene Composites Filled With Polydopamine-Functionalized Hexagonal Boron Nitride. PloS One 2017, 12 (1), e0170523 10.1371/journal.pone.0170523. 28107466 PMC5249180

[ref90] Zhang J.; Li S.-s.; Ji W.-x.; Zhang C.-w.; Li P.; Zhang S.-f.; Wang P.-j.; Yan S.-s. Two-dimensional GaGeTe Film: A Promising Graphene-like Material With Tunable Band Structure and High Carrier Mobility. J. Mater. Chem. C 2017, 5 (34), 8847–8853. 10.1039/C7TC03001K.

[ref91] Molle A.; Goldberger J.; Houssa M.; Xu Y.; Zhang S.-C.; Akinwande D. Buckled Two-dimensional Xene Sheets. Nat. Mater. 2017, 16 (2), 163–169. 10.1038/nmat4802. 28092688

[ref92] Cahangirov S.; Topsakal M.; Aktürk E.; Şahin H.; Ciraci S. Two-and one-dimensional Honeycomb Structures of Silicon and Germanium. Phys. Rev. Lett. 2009, 102 (23), 236804 10.1103/PhysRevLett.102.236804. 19658958

[ref93] Balendhran S.; Walia S.; Nili H.; Sriram S.; Bhaskaran M. Elemental Analogues of Graphene: Silicene, Germanene, Stanene, and Phosphorene. Small 2015, 11 (6), 640–652. 10.1002/smll.201402041. 25380184

[ref94] Geim A. K.; Novoselov K. S. The Rise of Graphene. In Nanoscience and Technology: A Collection of Reviews from Nature Journals; World Scientific, 2010; pp 11–19.

[ref95] Naguib M.; Mochalin V. N.; Barsoum M. W.; Gogotsi Y. 25th anniversary article: MXenes: A New Family of Two-dimensional Materials. Adv. Mater. 2014, 26 (7), 992–1005. 10.1002/adma.201304138. 24357390

[ref96] Shahzad F.; Alhabeb M.; Hatter C. B.; Anasori B.; Man Hong S.; Koo C. M.; Gogotsi Y. Electromagnetic Interference Shielding with 2D Transition Metal Carbides (MXenes). Science 2016, 353 (6304), 1137–1140. 10.1126/science.aag2421. 27609888

[ref97] Liu H.; Duan C.; Yang C.; Shen W.; Wang F.; Zhu Z. A Novel Nitrite Biosensor based on the Direct Electrochemistry of Hemoglobin Immobilized on MXene-Ti_3_C_2_. Sens. Actuators, B 2015, 218, 60–66. 10.1016/j.snb.2015.04.090.

[ref98] Naguib M.; Come J.; Dyatkin B.; Presser V.; Taberna P.-L.; Simon P.; Barsoum M. W.; Gogotsi Y. MXene: A Promising Transition Metal Carbide Anode for Lithium-ion Batteries. Electrochem. Commun. 2012, 16 (1), 61–64. 10.1016/j.elecom.2012.01.002.

[ref99] Wu L.; Lu X.; Dhanjai; Wu Z.-S.; Dong Y.; Wang X.; Zheng S.; Chen J. 2D Transition Metal Carbide MXene as a Robust Biosensing Platform for Enzyme Immobilization and Ultrasensitive Detection of Phenol. Biosens. Bioelectron. 2018, 107, 69–75. 10.1016/j.bios.2018.02.021. 29448223

[ref100] Huang K.; Li Z.; Lin J.; Han G.; Huang P. Two-dimensional Transition Metal Carbides and Nitrides (MXenes) for Biomedical Applications. Chem. Soc. Rev. 2018, 47 (14), 5109–5124. 10.1039/C7CS00838D. 29667670

[ref101] Wang F.; Wang Z.; Wang Q.; Wang F.; Yin L.; Xu K.; Huang Y.; He J. Synthesis, Properties and Applications of 2D Non-graphene Materials. Nanotechnology 2015, 26 (29), 292001 10.1088/0957-4484/26/29/292001. 26134271

[ref102] Lei J.-C.; Zhang X.; Zhou Z. Recent Advances in MXene: Preparation, Properties, and Applications. Frontiers of Physics 2015, 10 (3), 276–286. 10.1007/s11467-015-0493-x.

[ref103] Naguib M.; Kurtoglu M.; Presser V.; Lu J.; Niu J.; Heon M.; Hultman L.; Gogotsi Y.; Barsoum M. W. Two-dimensional Nanocrystals Produced by Exfoliation of Ti_3_AlC_2_. Adv. Mater. 2011, 23 (37), 4248–4253. 10.1002/adma.201102306. 21861270

[ref104] Yoon S.; In I. Solubilization of Reduced Graphene in Water Through Noncovalent Interaction With Dendrimers. Chem. Lett. 2010, 39 (11), 1160–1161. 10.1246/cl.2010.1160.

[ref105] Geim A. K.; Grigorieva I. V. van der Waals Heterostructures. Nature 2013, 499 (7459), 419–425. 10.1038/nature12385. 23887427

[ref106] Pollard T.; Earnshaw W.; Lippincott-Schwartz J.; Johnson G. Chapter 4-Biophysical Principles. Cell Biology, 3rd ed.; Elsevier, 2017; pp 53–62.

[ref107] Sanchez V. C.; Jachak A.; Hurt R. H.; Kane A. B. Biological Interactions of Graphene-Family Nanomaterials: An Interdisciplinary Review. Chem. Res. Toxicol. 2012, 25 (1), 15–34. 10.1021/tx200339h. 21954945 PMC3259226

[ref108] Lee J. D. Concise Inorganic Chemistry; John Wiley & Sons, 2008.

[ref109] Park Y.-J.; Park S. Y.; In I. Preparation of Water Soluble Graphene Using Polyethylene Glycol: Comparison of Covalent Approach and Noncovalent Approach. Journal of Industrial and Engineering Chemistry 2011, 17 (2), 298–303. 10.1016/j.jiec.2011.02.027.

[ref110] Liu G.; Ye H.; Li A.; Zhu C.; Jiang H.; Liu Y.; Han K.; Zhou Y. Graphene Oxide for High-Efficiency Separation Membranes: Role of Electrostatic Interactions. Carbon 2016, 110, 56–61. 10.1016/j.carbon.2016.09.005.

[ref111] Sabio J.; Seoanez C.; Fratini S.; Guinea F.; Neto A. C.; Sols F. Electrostatic Interactions between Graphene Layers and their Environment. Phys. Rev. B 2008, 77 (19), 195409 10.1103/PhysRevB.77.195409.

[ref112] Podeszwa R. Interactions of Graphene Sheets Deduced from Properties of Polycyclic Aromatic Hydrocarbons. J. Chem. Phys. 2010, 132 (4), 044704 10.1063/1.3300064. 20113056

[ref113] Cong J.; Chen Y.; Luo J.; Liu X. Fabrication of Graphene/Polyaniline Composite Multilayer Films by Electrostatic Layer-By-Layer Assembly. J. Solid State Chem. 2014, 218, 171–177. 10.1016/j.jssc.2014.06.037.

[ref114] Gupta A.; Arunachalam V.; Vasudevan S. Water Dispersible, Positively and Negatively Charged MoS2 Nanosheets: Surface Chemistry and the Role of Surfactant Binding. J. Phys. Chem. Lett. 2015, 6 (4), 739–744. 10.1021/acs.jpclett.5b00158. 26262496

[ref115] Guo J.; Yao X.; Ning L.; Wang Q.; Liu H. The Adsorption Mechanism and Induced Conformational Changes of Three Typical Proteins with Different Secondary Structural Features on Graphene. RSC Adv. 2014, 4 (20), 9953–9962. 10.1039/c3ra45876h.

[ref116] Liu B.; Salgado S.; Maheshwari V.; Liu J. DNA Adsorbed on Graphene and Graphene Oxide: Fundamental Interactions, Desorption and Applications. Curr. Opin. Colloid Interface Sci. 2016, 26, 41–49. 10.1016/j.cocis.2016.09.001.

[ref117] Viswanathan S.; Narayanan T. N.; Aran K.; Fink K. D.; Paredes J.; Ajayan P. M.; Filipek S.; Miszta P.; Tekin H. C.; Inci F.; et al. Graphene–protein Field Effect Biosensors: Glucose Sensing. Mater. Today 2015, 18 (9), 513–522. 10.1016/j.mattod.2015.04.003.

[ref118] Gu Z.; Song W.; Chen S. H.; Li B.; Li W.; Zhou R. Defect-assisted Protein HP35 Denaturation on Graphene. Nanoscale 2019, 11 (41), 19362–19369. 10.1039/C9NR01143A. 31099814

[ref119] Novoselov K. S.; Mishchenko A.; Carvalho A.; Castro Neto A. H. 2D Materials and van der Waals Heterostructures. Science 2016, 353 (6298), aac9439 10.1126/science.aac9439. 27471306

[ref120] Lu Z.; Dunn M. L. van der Waals Adhesion of Graphene Membranes. J. Appl. Phys. 2010, 107 (4), 044301 10.1063/1.3270425.

[ref121] Zhao Y.; Hu Z. Graphene in Ionic Liquids: Collective van der Waals Interaction and Hindrance of Self-Assembly Pathway. J. Phys. Chem. B 2013, 117 (36), 10540–10547. 10.1021/jp405660d. 23957744

[ref122] Liu L.; Zhang R.; Liu Y.; Tan W.; Zhu G. Insight into Hydrogen Bonds and Characterization of Interlayer Spacing of Hydrated Graphene Oxide. J. Mol. Model. 2018, 24 (6), 137 10.1007/s00894-018-3679-7. 29808444

[ref123] Li D.; Zhang W.; Yu X.; Wang Z.; Su Z.; Wei G. When Biomolecules Meet Graphene: From Molecular Level Interactions to Material Design and Applications. Nanoscale 2016, 8 (47), 19491–19509. 10.1039/C6NR07249F. 27878179

[ref124] Russo C. J.; Passmore L. A. Controlling Protein Adsorption on Graphene for Cryo-EM Using Low-Energy Hydrogen Plasmas. Nat. Methods 2014, 11 (6), 649–652. 10.1038/nmeth.2931. 24747813 PMC4141966

[ref125] Kim J.; Cote L. J.; Kim F.; Yuan W.; Shull K. R.; Huang J. Graphene Oxide Sheets at Interfaces. J. Am. Chem. Soc. 2010, 132 (23), 8180–8186. 10.1021/ja102777p. 20527938

[ref126] Mahmoudi M.; Kalhor H. R.; Laurent S.; Lynch I. Protein Fibrillation and Nanoparticle Interactions: Opportunities and Challenges. Nanoscale 2013, 5 (7), 2570–2588. 10.1039/c3nr33193h. 23463168

[ref127] Zhang W.; Huynh T.; Xiu P.; Zhou B.; Ye C.; Luan B.; Zhou R. Revealing the Importance of Surface Morphology of Nanomaterials to Biological Responses: Adsorption of the Villin Headpiece onto Graphene and Phosphorene. Carbon 2015, 94, 895–902. 10.1016/j.carbon.2015.07.075.

[ref128] Huang Y.; Qiao J.; He K.; Bliznakov S.; Sutter E.; Chen X.; Luo D.; Meng F.; Su D.; Decker J.; et al. Interaction of Black Phosphorus with Oxygen and Water. Chem. Mater. 2016, 28 (22), 8330–8339. 10.1021/acs.chemmater.6b03592.

[ref129] Hernandez Y.; Nicolosi V.; Lotya M.; Blighe F. M.; Sun Z.; De S.; McGovern I.; Holland B.; Byrne M.; Gun’Ko Y. K.; et al. High-Yield Production of Graphene by Liquid-Phase Exfoliation of Graphite. Nat. Nanotechnol. 2008, 3 (9), 563–568. 10.1038/nnano.2008.215. 18772919

[ref130] Stankovich S.; Piner R. D.; Chen X.; Wu N.; Nguyen S. T.; Ruoff R. S. Stable Aqueous Dispersions of Graphitic Nanoplatelets via the Reduction of Exfoliated Graphite Oxide in the Presence of Poly (Sodium 4-Styrenesulfonate). J. Mater. Chem. 2006, 16 (2), 155–158. 10.1039/B512799H.

[ref131] Xu Y.; Bai H.; Lu G.; Li C.; Shi G. Flexible Graphene Films via the Filtration of Water-Soluble Noncovalent Functionalized Graphene Sheets. J. Am. Chem. Soc. 2008, 130 (18), 5856–5857. 10.1021/ja800745y. 18399634

[ref132] Yang H.; Zhang Q.; Shan C.; Li F.; Han D.; Niu L. Stable, Conductive Supramolecular Composite of Graphene Sheets with Conjugated Polyelectrolyte. Langmuir 2010, 26 (9), 6708–6712. 10.1021/la100365z. 20229996

[ref133] Koenig S. P.; Boddeti N. G.; Dunn M. L.; Bunch J. S. Ultrastrong Adhesion of Graphene Membranes. Nat. Nanotechnol. 2011, 6 (9), 543 10.1038/nnano.2011.123. 21841794

[ref134] Zhang H.; Zhang T.; Wang Y. Mechanistic Understanding and Binding Analysis of Two-Dimensional MoS2 Nanosheets with Human Serum Albumin by the Biochemical and Biophysical Approach. Spectrochimica Acta Part A: Molecular and Biomolecular Spectroscopy 2019, 211, 18–25. 10.1016/j.saa.2018.11.055. 30502580

[ref135] Jokar S.; Pourjavadi A.; Adeli M. Albumin–Graphene Oxide Conjugates; Carriers for Anticancer Drugs. RSC Adv. 2014, 4 (62), 33001–33006. 10.1039/C4RA05752J.

[ref136] Nan Z.; Hao C.; Ye X.; Feng Y.; Sun R. Interaction of Graphene Oxide with Bovine Serum Albumin: A Fluorescence Quenching Study. Spectrochimica Acta Part A: Molecular and Biomolecular Spectroscopy 2019, 210, 348–354. 10.1016/j.saa.2018.11.028. 30476875

[ref137] Zhang J.; Zhang F.; Yang H.; Huang X.; Liu H.; Zhang J.; Guo S. Graphene Oxide as a Matrix for Enzyme Immobilization. Langmuir 2010, 26 (9), 6083–6085. 10.1021/la904014z. 20297789

[ref138] Huang A.; Li W.; Shi S.; Yao T. Quantitative Fluorescence Quenching on Antibody-Conjugated Graphene Oxide as a Platform for Protein Sensing. Sci. Rep. 2017, 7 (1), 40772 10.1038/srep40772. 28084438 PMC5233999

[ref139] Wei X.-Q.; Hao L.-Y.; Shao X.-R.; Zhang Q.; Jia X.-Q.; Zhang Z.-R.; Lin Y.-F.; Peng Q. Insight into the Interaction of Graphene Oxide with Serum Proteins and the Impact of the Degree of Reduction and Concentration. ACS Appl. Mater. Interfaces 2015, 7 (24), 13367–13374. 10.1021/acsami.5b01874. 26029973

[ref140] Liu J.; Fu S.; Yuan B.; Li Y.; Deng Z. Toward a Universal “Adhesive Nanosheet” for the Assembly of Multiple Nanoparticles based on a Protein-Induced Reduction/Decoration of Graphene Oxide. J. Am. Chem. Soc. 2010, 132 (21), 7279–7281. 10.1021/ja100938r. 20462190

[ref141] Duan G.; Kang S.-g.; Tian X.; Garate J. A.; Zhao L.; Ge C.; Zhou R. Protein Corona Mitigates the Cytotoxicity of Graphene Oxide by Reducing its Physical Interaction with Cell Membrane. Nanoscale 2015, 7 (37), 15214–15224. 10.1039/C5NR01839K. 26315610

[ref142] Chong Y.; Ge C.; Yang Z.; Garate J. A.; Gu Z.; Weber J. K.; Liu J.; Zhou R. Reduced Cytotoxicity of Graphene Nanosheets Mediated by Blood–protein Coating. ACS Nano 2015, 9 (6), 5713–5724. 10.1021/nn5066606. 26040772

[ref143] Katoch J.; Kim S. N.; Kuang Z.; Farmer B. L.; Naik R. R.; Tatulian S. A.; Ishigami M. Structure of a Peptide Adsorbed on Graphene and Graphite. Nano Lett. 2012, 12 (5), 2342–2346. 10.1021/nl300286k. 22471315

[ref144] Hu W.; Peng C.; Lv M.; Li X.; Zhang Y.; Chen N.; Fan C.; Huang Q. Protein Corona-Mediated Mitigation of Cytotoxicity of Graphene Oxide. ACS Nano 2011, 5 (5), 3693–3700. 10.1021/nn200021j. 21500856

[ref145] Baimanov D.; Wu J.; Chu R.; Cai R.; Wang B.; Cao M.; Tao Y.; Liu J.; Guo M.; Wang J.; et al. Immunological Responses Induced by Blood Protein Coronas on Two-dimensional MoS2 Nanosheets. ACS Nano 2020, 14 (5), 5529–5542. 10.1021/acsnano.9b09744. 32283010

[ref146] Lu F.; Zhang S.; Gao H.; Jia H.; Zheng L. Protein-decorated Reduced Oxide Graphene Composite and its Application to SERS. ACS Appl. Mater. Interfaces 2012, 4 (6), 3278–3284. 10.1021/am300634n. 22692825

[ref147] Kotchey G. P.; Allen B. L.; Vedala H.; Yanamala N.; Kapralov A. A.; Tyurina Y. Y.; Klein-Seetharaman J.; Kagan V. E.; Star A. The Enzymatic Oxidation of Graphene oxide. ACS Nano 2011, 5 (3), 2098–2108. 10.1021/nn103265h. 21344859 PMC3062704

[ref148] Kenry K.; Loh K. P.; Lim C. T. Molecular Interactions of Graphene Oxide with Human Blood Plasma Proteins. Nanoscale 2016, 8 (17), 9425–9441. 10.1039/C6NR01697A. 27094022

[ref149] Han M.; Zhu L.; Mo J.; Wei W.; Yuan B.; Zhao J.; Cao C. Protein Corona and Immune Responses of Borophene: A Comparison of Nanosheet–Plasma Interface with Graphene and Phosphorene. ACS Appl. Bio Mater. 2020, 3, 4220 10.1021/acsabm.0c00306. 35025423

[ref150] Mu Q.; Su G.; Li L.; Gilbertson B. O.; Yu L. H.; Zhang Q.; Sun Y.-P.; Yan B. Size-dependent cell uptake of protein-coated graphene oxide nanosheets. ACS Appl. Mater. Interfaces 2012, 4 (4), 2259–2266. 10.1021/am300253c. 22409495

[ref151] Huang C.; Bai H.; Li C.; Shi G. A Graphene Oxide/Hemoglobin Composite Hydrogel for Enzymatic Catalysis in Organic Solvents. Chem. Commun. 2011, 47 (17), 4962–4964. 10.1039/c1cc10412h. 21431118

[ref152] Rozmysłowska-Wojciechowska A.; Wojciechowski T.; Ziemkowska W.; Chlubny L.; Olszyna A.; Jastrzębska A. Surface Interactions Between 2D Ti3C2/Ti2C MXenes and Lysozyme. Appl. Surf. Sci. 2019, 473, 409–418. 10.1016/j.apsusc.2018.12.081.

[ref153] Malik S. A.; Mohanta Z.; Srivastava C.; Atreya H. S. Modulation of Protein–Graphene Oxide Interactions with Varying Degrees of Oxidation. Nanoscale Adv. 2020, 2 (5), 1904–1912. 10.1039/C9NA00807A. 36132498 PMC9419239

[ref154] Li S.; Aphale A. N.; Macwan I. G.; Patra P. K.; Gonzalez W. G.; Miksovska J.; Leblanc R. M. Graphene Oxide as a Quencher for Fluorescent Assay of Amino Acids, Peptides, and Proteins. ACS Appl. Mater. Interfaces 2012, 4 (12), 7069–7075. 10.1021/am302704a. 23173615

[ref155] Wang Y.; Zhu Z.; Zhang H.; Chen J.; Tang B.; Cao J. Investigation on the Conformational Structure of Hemoglobin on Graphene Oxide. Mater. Chem. Phys. 2016, 182, 272–279. 10.1016/j.matchemphys.2016.07.032.

[ref156] Kukkar M.; Sharma A.; Kumar P.; Kim K.-H.; Deep A. Application of MoS2Modified Screen-Printed Electrodes for Highly Sensitive Detection of Bovine Serum Albumin. Anal. Chim. Acta 2016, 939, 101–107. 10.1016/j.aca.2016.08.010. 27639148

[ref157] Zhang H.; Han Q.; Yin X.; Wang Y. Insights into the Binding Mechanism of Two-Dimensional Black Phosphorus Nanosheets–protein Associations. Spectrochimica Acta Part A: Molecular and Biomolecular Spectroscopy 2020, 227, 117662 10.1016/j.saa.2019.117662. 31654845

[ref158] Patel A. S.; Mishra P.; Kanaujia P. K.; Husain S. S.; Vijaya Prakash G.; Chakraborti A. Investigating Resonance Energy Transfer from Protein Molecules to van der Waals Nanosheets. RSC Adv. 2017, 7 (42), 26250–26255. 10.1039/C7RA02376F.

[ref159] De M.; Chou S. S.; Dravid V. P. Graphene Oxide as an Enzyme Inhibitor: Modulation of Activity of α-Chymotrypsin. J. Am. Chem. Soc. 2011, 133 (44), 17524–17527. 10.1021/ja208427j. 21954932 PMC3241955

[ref160] Yao K.; Tan P.; Luo Y.; Feng L.; Xu L.; Liu Z.; Li Y.; Peng R. Graphene Oxide Selectively Enhances Thermostability of Trypsin. ACS Appl. Mater. Interfaces 2015, 7 (22), 12270–12277. 10.1021/acsami.5b03118. 25985836

[ref161] Li H.; Fierens K.; Zhang Z.; Vanparijs N.; Schuijs M. J.; Van Steendam K.; Feiner Gracia N. l.; De Rycke R.; De Beer T.; De Beuckelaer A.; et al. Spontaneous Protein Adsorption on Graphene Oxide Nanosheets Allowing Efficient Intracellular Vaccine Protein Delivery. ACS Appl. Mater. Interfaces 2016, 8 (2), 1147–1155. 10.1021/acsami.5b08963. 26694764

[ref162] Gu Z.; Yang Z.; Kang S.-g.; Yang J. R.; Luo J.; Zhou R. Robust Denaturation of Villin Headpiece by MoS2 Nanosheet: Potential Molecular Origin of the Nanotoxicity. Sci. Rep. 2016, 6 (1), 28252 10.1038/srep28252. 27312409 PMC4911589

[ref163] Feng M.; Kang H.; Yang Z.; Luan B.; Zhou R. Potential Disruption of Protein–protein Interactions by Graphene Oxide. J. Chem. Phys. 2016, 144 (22), 225102 10.1063/1.4953562. 27306022

[ref164] Luan B.; Huynh T.; Zhao L.; Zhou R. Potential Toxicity of Graphene to Cell Functions via Disrupting Protein–Protein Interactions. ACS Nano 2015, 9 (1), 663–669. 10.1021/nn506011j. 25494677

[ref165] Sun X.; Feng Z.; Hou T.; Li Y. Mechanism of Graphene Oxide as an Enzyme Inhibitor from Molecular Dynamics Simulations. ACS Appl. Mater. Interfaces 2014, 6 (10), 7153–7163. 10.1021/am500167c. 24801143

[ref166] Atabay M.; Jahanbin Sardroodi J.; Rastkar Ebrahimzadeh A. Adsorption and Immobilisation of Human Insulin on Graphene Monoxide, Silicon Carbide and Boron Nitride Nanosheets Investigated by Molecular Dynamics Simulation. Mol. Simul. 2017, 43 (4), 298–311. 10.1080/08927022.2016.1270452.

[ref167] Xiao M.; Wei S.; Li Y.; Jasensky J.; Chen J.; Brooks C. L.; Chen Z. Molecular Interactions Between Single Layered MoS2 and Biological Molecules. Chem. Sci. 2018, 9 (7), 1769–1773. 10.1039/C7SC04884J. 29675220 PMC5885976

[ref168] Bayan R.; Karak N. Polymer Nanocomposites Based on Two-Dimensional Nanomaterials. In Two-Dimensional Nanostructures for Biomedical Technology; Elsevier, 2020; pp 249–279.

[ref169] Jensen E. Types of Imaging, Part 3: Atomic Force Microscopy. Anatomical Record 2013, 296 (2), 179–183. 10.1002/ar.22605. 23074163

[ref170] Cohen S. R.; Bitler A. Use of AFM in Bio-related Systems. Curr. Opin. Colloid Interface Sci. 2008, 13 (5), 316–325. 10.1016/j.cocis.2008.02.002.

[ref171] Gaczynska M.; Osmulski P. A. AFM of Biological Complexes: What Can We Learn?. Curr. Opin. Colloid Interface Sci. 2008, 13 (5), 351–367. 10.1016/j.cocis.2008.01.004. 19802337 PMC2630216

[ref172] Sitterberg J.; Özcetin A.; Ehrhardt C.; Bakowsky U. Utilising Atomic Force Microscopy for the Characterisation of Nanoscale Drug Delivery Systems. Eur. J. Pharm. Biopharm. 2010, 74 (1), 2–13. 10.1016/j.ejpb.2009.09.005. 19755155

[ref173] Erni R.; Rossell M. D.; Kisielowski C.; Dahmen U. Atomic-Resolution Imaging with a Sub-50-pm Electron Probe. Phys. Rev. Lett. 2009, 102 (9), 096101 10.1103/PhysRevLett.102.096101. 19392535

[ref174] Trivedi V.; Chaudhary N. Bioanalytical Techniques and Bioinformatics; Indian Institute of Technology Guwahati, 2014.

[ref175] Akhtar K.; Khan S. A.; Khan S. B.; Asiri A. M. Scanning Electron Microscopy: Principle and Applications in Nanomaterials Characterization. In Handbook of Materials Characterization; Springer, 2018; pp 113–145.

[ref176] Ma D.; Xie C.; Wang T.; Mei L.; Zhang X.; Guo Z.; Yin W. Liquid-Phase Exfoliation and Functionalization of MoS2 Nanosheets for Effective Antibacterial Application. ChemBioChem. 2020, 21 (16), 2373–2380. 10.1002/cbic.202000195. 32227558

[ref177] Arzenšek D.; Kuzman D.; Podgornik R. Colloidal Interactions between Monoclonal Antibodies in Aqueous Solutions. J. Colloid Interface Sci. 2012, 384 (1), 207–216. 10.1016/j.jcis.2012.06.055. 22840854

[ref178] Tüzün U. u.; Farhadpour F. A. Dynamic Particle Size Analysis with Light Scattering Technique. Part. Part. Syst. Charact. 1986, 3 (4), 151–157. 10.1002/ppsc.19860030403.

[ref179] Sapsford K. E.; Tyner K. M.; Dair B. J.; Deschamps J. R.; Medintz I. L. Analyzing Nanomaterial Bioconjugates: A Review of Current and Emerging Purification and Characterization Techniques. Anal. Chem. 2011, 83 (12), 4453–4488. 10.1021/ac200853a. 21545140

[ref180] Mittal P.; Vardhan H.; Ajmal G.; Bonde G. V.; Kapoor R.; Mittal A.; Mishra B. Formulation, Optimization, Hemocompatibility and Pharmacokinetic Evaluation of PLGA Nanoparticles Containing Paclitaxel. Drug Dev. Ind. Pharm. 2019, 45 (3), 365–378. 10.1080/03639045.2018.1542706. 30394795

[ref181] Kumar A.; Dixit C. K. Methods for Characterization of Nanoparticles. In Advances in Nanomedicine for the Delivery of Therapeutic Nucleic Acids; Elsevier: 2017; pp 43–58.

[ref182] Luo N.; Ni D.; Yue H.; Wei W.; Ma G. Surface-engineered graphene navigate divergent biological outcomes toward macrophages. ACS Appl. Mater. Interfaces 2015, 7 (9), 5239–5247. 10.1021/am5084607. 25692327

[ref183] Mahato K.; Purohit B.; Bhardwaj K.; Jaiswal A.; Chandra P. Novel Electrochemical Biosensor for Serotonin Detection Based on Gold Nanorattles Decorated Reduced Graphene Oxide in Biological Fluids and In Vitro Model. Biosens. Bioelectron. 2019, 142, 111502 10.1016/j.bios.2019.111502. 31326860

[ref184] Provost J. J. Principles and techniques of biochemistry and molecular biology: Wilson, Keith, and Walker, John. Biochemistry and Molecular Biology Education 2005, 33 (5), 379–380. 10.1002/bmb.2005.49403305379.

[ref185] Wilson K.; Walker J. Principles and Techniques of Biochemistry and Molecular Biology; Cambridge University Press, 2010.

[ref186] Herschel J. F. W. IV. ’Aμóρϕωτa, no. I.--On a case of superficial colour presented by a homogeneous liquid internally colourless. Philos. Trans. R. Soc. London 1845, (135), 143–145. 10.1098/rstl.1845.0004.

[ref187] Röcker C.; Pötzl M.; Zhang F.; Parak W. J.; Nienhaus G. U. A Quantitative Fluorescence Study of Protein Monolayer Formation on Colloidal Nanoparticles. Nat. Nanotechnol. 2009, 4 (9), 577–580. 10.1038/nnano.2009.195. 19734930

[ref188] Chiu Y.-L.; Chen S.-A.; Chen J.-H.; Chen K.-J.; Chen H.-L.; Sung H.-W. A Dual-Emission Forster Resonance Energy Transfer Nanoprobe for Sensing/Imaging pH Changes in the Biological Environment. ACS Nano 2010, 4 (12), 7467–7474. 10.1021/nn102644u. 21082810

[ref189] Govorov A.; Martínez P. L. H.; Demir H. V. Understanding and Modeling Förster-type Resonance Energy Transfer (FRET): Introduction to FRET; Springer, 2016; Vol. 1.

[ref190] Geldert A.; Kenry K.; Zhang X.; Zhang H.; Lim C. T. Enhancing the Sensing Specificity of a MoS2 Nanosheet-based FRET Aptasensor using a Surface Blocking Strategy. Analyst 2017, 142 (14), 2570–2577. 10.1039/C7AN00640C. 28569315

[ref191] Seo J.-W. T.; Green A. A.; Antaris A. L.; Hersam M. C. High-Concentration Aqueous Dispersions of Graphene using Nonionic, Biocompatible Block Copolymers. J. Phys. Chem. Lett. 2011, 2 (9), 1004–1008. 10.1021/jz2003556.

[ref192] Hong B. J.; Compton O. C.; An Z.; Eryazici I.; Nguyen S. T. Successful Stabilization of Graphene Oxide in Electrolyte Solutions: Enhancement of Biofunctionalization and Cellular Uptake. ACS Nano 2012, 6 (1), 63–73. 10.1021/nn202355p. 22017285 PMC3642249

[ref193] Zhang M.; Yin B.-C.; Wang X.-F.; Ye B.-C. Interaction of Peptides with Graphene Oxide and its Application for Real-Time Monitoring of Protease Activity. Chem. Commun. 2011, 47 (8), 2399–2401. 10.1039/C0CC04887A. 21305066

[ref194] Introduction to Fourier Transform Infrared Spectrometry; Thermo Nicolet Corporation, 2001.

[ref195] Wagner M. S.; McArthur S. L.; Shen M.; Horbett T. A.; Castner D. G. Limits of Detection for Time of Flight Secondary Ion Mass Spectrometry (Tof-SIMS) and X-Ray Photoelectron Spectroscopy (XPS): Detection of Low Amounts of Adsorbed Protein. Journal of Biomaterials Science, Polymer Edition 2002, 13 (4), 407–428. 10.1163/156856202320253938. 12160301

[ref196] Briggs D. Surface Analysis of Polymers by XPS and Static SIMS; Cambridg University Press, 1998.

[ref197] Paynter R.; Ratner B.; Horbett T.; Thomas H. XPS Studies on the Organization of Adsorbed Protein Films on Fluoropolymers. J. Colloid Interface Sci. 1984, 101 (1), 233–245. 10.1016/0021-9797(84)90023-7.

[ref198] Greenfield N. J. Circular Dichroism (CD) Analyses of Protein–protein Interactions. In Protein–Protein Interactions; Springer, 2015; pp 239–265. 10.1007/978-1-4939-2425-7_15 25859954

[ref199] Kumar S.; Parekh S. H. Linking Graphene-based Material Physicochemical Properties with Molecular Adsorption, Structure and Cell Fate. Commun. Chem. 2020, 3 (1), 8 10.1038/s42004-019-0254-9. 36703309 PMC9814659

[ref200] Lee W. C.; Lim C. H. Y.; Shi H.; Tang L. A.; Wang Y.; Lim C. T.; Loh K. P. Origin of Enhanced Stem Cell Growth and Differentiation on Graphene and Graphene Oxide. ACS Nano 2011, 5 (9), 7334–7341. 10.1021/nn202190c. 21793541

[ref201] Freire E. I.; Mayorga O. L.; Straume M. Isothermal Titration. Anal. Chem. 1990, 62 (18), 950A–959. 10.1021/ac00217a002.

[ref202] Allen M. P. Introduction to Molecular Dynamics Simulation. In Computational Soft Matter: From Synthetic Polymers to Proteins, Lecture Notes; Attig N., Binder K., Grubmuller H., Kremer K., Eds.; NIC Series, Vol. 23; John von Neumann Institute for Computing, Julich, 2004.

[ref203] Karplus M.; McCammon J. A. Molecular Dynamics Simulations of Biomolecules. Nat. Struct. Biol. 2002, 9 (9), 646–652. 10.1038/nsb0902-646. 12198485

[ref204] Vatanparast M.; Shariatinia Z. Hexagonal Boron Nitride Nanosheet as Novel Drug Delivery System for Anticancer Drugs: Insights from DFT Calculations and Molecular Dynamics Simulations. J. Mol. Graphics Modell. 2019, 89, 50–59. 10.1016/j.jmgm.2019.02.012. 30870649

[ref205] Mücksch C.; Urbassek H. M. Molecular Dynamics Simulation of Free and Forced BSA Adsorption on a Hydrophobic Graphite Surface. Langmuir 2011, 27 (21), 12938–12943. 10.1021/la201972f. 21877733

[ref206] Bisht D.; Rath S.; Roy S.; Jaiswal A. MoS2 Nanosheets Effectively Binds to the Receptor Binding Domain of SARS-CoV-2 Spike Protein and Destabilizes the Spike-Human ACE2 Receptor Interactions. Soft Matter 2022, 18, 8961 10.1039/D2SM01181F. 36382499

[ref207] Kudelski A. Analytical Applications of Raman Spectroscopy. Talanta 2008, 76 (1), 1–8. 10.1016/j.talanta.2008.02.042. 18585231

[ref208] Jin S.; Li K.; Li J. A General Bio-inspired, Novel Interface Engineering Strategy Toward Strong yet Tough Protein based Composites. Appl. Surf. Sci. 2018, 447, 452–462. 10.1016/j.apsusc.2018.03.073.

[ref209] Zhang Y.; Zhang J.; Huang X.; Zhou X.; Wu H.; Guo S. Assembly of Graphene oxide–Enzyme Conjugates through Hydrophobic Interaction. Small 2012, 8 (1), 154–159. 10.1002/smll.201101695. 22038754

[ref210] Ding Z.; Ma H.; Chen Y. Interaction of Graphene Oxide with Human Serum Albumin and its Mechanism. RSC Adv. 2014, 4 (98), 55290–55295. 10.1039/C4RA09613D.

[ref211] Ma B. K.; Li M.; Cheong L. Z.; Weng X. C.; Shen C.; Huang Q. Enzyme-MXene Nanosheets: Fabrication and Application in Electrochemical Detection of H_2_O_2_. J. Inorg. Mater. 2020, 35, 131–138. 10.15541/jim20190139.

[ref212] Yang Z.; Ge C.; Liu J.; Chong Y.; Gu Z.; Jimenez-Cruz C. A.; Chai Z.; Zhou R. Destruction of Amyloid Fibrils by Graphene through Penetration and Extraction of Peptides. Nanoscale 2015, 7 (44), 18725–18737. 10.1039/C5NR01172H. 26503908

[ref213] Li M.; Zhao A.; Dong K.; Li W.; Ren J.; Qu X. Chemically Exfoliated WS_2_ Nanosheets Efficiently Inhibit Amyloid β-peptide Aggregation and can be used for Photothermal Treatment of Alzheimer’s Disease. Nano Res. 2015, 8, 3216–3227. 10.1007/s12274-015-0821-z.

[ref214] Yan Z.-S.; Li X.-L.; Ma Y.-Q.; Ding H.-M. Effect of the Graphene Nanosheet on Functions of the Spike Protein in Open and Closed States: Comparison between SARS-CoV-2 Wild Type and the Omicron Variant. Langmuir 2022, 38 (45), 13972–13982. 10.1021/acs.langmuir.2c02316. 36318181

[ref215] Wu R.; Ou X.; Zhang L.; Wang F.; Liu L. Interfacial Interactions within Amyloid Protein Corona Based on 2D MoS2 Nanosheets. ChemBioChem. 2022, 23 (2), e202100581 10.1002/cbic.202100581. 34708897

[ref216] Wu D.; Guo A.; Guo Z.; Xie L.; Wei Q.; Du B. Simultaneous Electrochemical Detection of Cervical Cancer Markers using Reduced Graphene Oxide-Tetraethylene Pentamine as Electrode Materials and Distinguishable Redox Probes as Labels. Biosens. Bioelectron. 2014, 54, 634–639. 10.1016/j.bios.2013.11.042. 24333936

[ref217] Singh V. K.; Kumar S.; Pandey S. K.; Srivastava S.; Mishra M.; Gupta G.; Malhotra B.; Tiwari R.; Srivastava A. Fabrication of Sensitive Bioelectrode based on Atomically Thin CVD Grown Graphene for Cancer Biomarker Detection. Biosens. Bioelectron. 2018, 105, 173–181. 10.1016/j.bios.2018.01.014. 29412942

[ref218] Jin B.; Wang P.; Mao H.; Hu B.; Zhang H.; Cheng Z.; Wu Z.; Bian X.; Jia C.; Jing F.; et al. Multi-Nanomaterial Electrochemical Biosensor based on Label-free Graphene for Detecting Cancer Biomarkers. Biosens. Bioelectron. 2014, 55, 464–469. 10.1016/j.bios.2013.12.025. 24462797

[ref219] Wang M.; Li J.; Chen J.; Zhang Y.; Jia Y.; Yang H.; Kong J. Ultrasensitive Electrochemical Immunosensor via RAFT Polymerization Signal Amplification for the Detection of Lung Cancer Biomarker. J. Electroanal. Chem. 2021, 882, 114971 10.1016/j.jelechem.2020.114971.

[ref220] Cao X. Ultra-sensitive Electrochemical DNA Biosensor based on Signal Amplification using Gold Nanoparticles Modified with Molybdenum Disulfide, Graphene and Horseradish Peroxidase. Microchim. Acta 2014, 181, 1133–1141. 10.1007/s00604-014-1301-y.

[ref221] Huang K.-J.; Wang L.; Li J.; Liu Y.-M. Electrochemical Sensing based on Layered MoS_2_–graphene Composites. Sens. Actuators, B 2013, 178, 671–677. 10.1016/j.snb.2013.01.028.

[ref222] Wu S.; Zeng Z.; He Q.; Wang Z.; Wang S. J.; Du Y.; Yin Z.; Sun X.; Chen W.; Zhang H. Electrochemically Reduced Single-layer MoS2 Nanosheets: Characterization, Properties, and Sensing Applications. Small 2012, 8 (14), 2264–2270. 10.1002/smll.201200044. 22532527

[ref223] Su S.; Sun H.; Xu F.; Yuwen L.; Wang L. Highly Sensitive and Selective Determination of Dopamine in the Presence of Ascorbic Acid using Gold Nanoparticles-Decorated MoS_2_ Nanosheets Modified Electrode. Electroanalysis 2013, 25 (11), 2523–2529. 10.1002/elan.201300332.

[ref224] Zhao L.; Cheng M.; Liu G.; Lu H.; Gao Y.; Yan X.; Liu F.; Sun P.; Lu G. A Fluorescent Biosensor based on Molybdenum Disulfide Nanosheets and Protein Aptamer for Sensitive Detection of Carcinoembryonic Antigen. Sens. Actuators, B 2018, 273, 185–190. 10.1016/j.snb.2018.06.004.

[ref225] Kong R.-M.; Ding L.; Wang Z.; You J.; Qu F. A Novel Aptamer-functionalized MoS_2_ Nanosheet Fluorescent Biosensor for Sensitive Detection of Prostate Specific Antigen. Anal. Bioanal. Chem. 2015, 407, 369–377. 10.1007/s00216-014-8267-9. 25366976

[ref226] Wang L.; Wang Y.; Wong J. I.; Palacios T.; Kong J.; Yang H. Y. Functionalized MoS_2_ Nanosheet-based Field-effect Biosensor for Label-free Sensitive Detection of Cancer Marker Proteins in Solution. Small 2014, 10 (6), 1101–1105. 10.1002/smll.201302081. 24474708

[ref227] Wu Q.; Li N.; Wang Y.; Xu Y.; Wu J.; Jia G.; Ji F.; Fang X.; Chen F.; Cui X. Ultrasensitive and Selective Determination of Carcinoembryonic Antigen using Multifunctional Ultrathin Amino-Functionalized Ti_3_C_2_-MXene Nanosheets. Anal. Chem. 2020, 92 (4), 3354–3360. 10.1021/acs.analchem.9b05372. 32011882

[ref228] Peng J.; Lai Y.; Chen Y.; Xu J.; Sun L.; Weng J. Sensitive Detection of Carcinoembryonic Antigen using Stability-limited Few-layer Black Phosphorus as an Electron Donor and a Reservoir. Small 2017, 13 (15), 1603589 10.1002/smll.201603589. 28112857

[ref229] Zhu X.; Xu Y.; Cheng Z.; Wang Y.; Lu Z.; Zhang G. First Principles Study of Atmospheric Pollutants Adsorption on Non-Defect and Monatomic Defect Graphene. Diamond Relat. Mater. 2021, 112, 108252 10.1016/j.diamond.2021.108252.

[ref230] Cao X.; Zhao J.; Wang Z.; Xing B. New Insight into the Photo-transformation Mechanisms of Graphene Oxide Under UV-A, UV-B and UV-C Lights. J. Hazard. Mater. 2021, 403, 123683 10.1016/j.jhazmat.2020.123683. 32846254

[ref231] Yang H.; Wu X.; Ma Q.; Yilihamu A.; Yang S.; Zhang Q.; Feng S.; Yang S.-T. Fungal Transformation of Graphene by White Rot Fungus *Phanerochaete chrysosporium*. Chemosphere 2019, 216, 9–18. 10.1016/j.chemosphere.2018.10.115. 30359921

[ref232] Wang X.; Liu L.; Liang D.; Liu Y.; Zhao Q.; Huang P.; Li X.; Fan W. Accumulation, Transformation and Subcellular Distribution of Arsenite Associated with Five Carbon Nanomaterials in Freshwater Zebrafish Specific-Tissues. J. Hazard. Mater. 2021, 415, 125579 10.1016/j.jhazmat.2021.125579. 33721782

[ref233] Cao X.; Ma C.; Zhao J.; Musante C.; White J. C.; Wang Z.; Xing B. Interaction of Graphene Oxide with Co-existing Arsenite and Arsenate: Adsorption, Transformation and Combined Toxicity. Environ. Int. 2019, 131, 104992 10.1016/j.envint.2019.104992. 31288181

[ref234] de Medeiros A. M. Z.; Côa F.; Alves O. L.; Martinez D. S. T.; Barbieri E. Metabolic Effects in the Freshwater Fish *Geophagus iporangensis* in Response to Single and Combined Exposure to Graphene Oxide and Trace Elements. Chemosphere 2020, 243, 125316 10.1016/j.chemosphere.2019.125316. 31733537

[ref235] Lee T.-W.; Chen C.-C.; Chen C. Chemical Stability and Transformation of Molybdenum disulfide Nanosheets in Environmental Media. Environ. Sci. Technol. 2019, 53 (11), 6282–6291. 10.1021/acs.est.9b00318. 31062596

[ref236] Wang Z.; von dem Bussche A.; Qiu Y.; Valentin T. M.; Gion K.; Kane A. B.; Hurt R. H. Chemical Dissolution Pathways of MoS2 Nanosheets in Biological and Environmental Media. Environ. Sci. Technol. 2016, 50 (13), 7208–7217. 10.1021/acs.est.6b01881. 27267956 PMC5217159

[ref237] Yamamoto M.; Einstein T. L.; Fuhrer M. S.; Cullen W. G. Anisotropic Etching of Atomically Thin MoS2. J. Phys. Chem. C 2013, 117 (48), 25643–25649. 10.1021/jp410893e.

[ref238] Liu Y.; Tan C.; Chou H.; Nayak A.; Wu D.; Ghosh R.; Chang H.-Y.; Hao Y.; Wang X.; Kim J.-S.; et al. Thermal Oxidation of WSe2 Nanosheets Adhered on Sio2/Si Substrates. Nano Lett. 2015, 15 (8), 4979–4984. 10.1021/acs.nanolett.5b02069. 26171759

[ref239] Gan D.; Huang Q.; Dou J.; Huang H.; Chen J.; Liu M.; Wen Y.; Yang Z.; Zhang X.; Wei Y. Bioinspired Functionalization of MXenes (Ti3C2TX) with Amino Acids for Efficient Removal of Heavy Metal Ions. Appl. Surf. Sci. 2020, 504, 144603 10.1016/j.apsusc.2019.144603.

[ref240] Bury D.; Jakubczak M.; Kumar R.; Ścieżyńska D.; Bogacki J.; Marcinowski P.; Jastrzębska A. M. Cleaning the Environment with MXenes. MRS Bull. 2023, 48, 271–282. 10.1557/s43577-023-00507-6.

[ref241] Peng Q.; Guo J.; Zhang Q.; Xiang J.; Liu B.; Zhou A.; Liu R.; Tian Y. Unique Lead Adsorption Behavior of Activated Hydroxyl Group in Two-Dimensional Titanium Carbide. J. Am. Chem. Soc. 2014, 136 (11), 4113–4116. 10.1021/ja500506k. 24588686

[ref242] Vasyukova I. A.; Zakharova O. V.; Kuznetsov D. V.; Gusev A. A. Synthesis, Toxicity Assessment, Environmental and Biomedical Applications of Mxenes: A Review. Nanomaterials 2022, 12 (11), 1797 10.3390/nano12111797. 35683652 PMC9182201

[ref243] Tu Z.; Guday G.; Adeli M.; Haag R. Multivalent Interactions between 2D Nanomaterials and Biointerfaces. Adv. Mater. 2018, 30 (33), 1706709 10.1002/adma.201706709. 29900600

[ref244] Depan D.; Misra R. The Interplay between Nanostructured Carbon-grafted Chitosan Scaffolds and Protein Adsorption on the Cellular Response of Osteoblasts: Structure–Function Property Relationship. Acta Biomater. 2013, 9 (4), 6084–6094. 10.1016/j.actbio.2012.12.019. 23261921

